# Advancing fair and explainable machine learning for neuroimaging dementia pattern classification in multi-racial and multi-ethnic populations

**DOI:** 10.1038/s41467-026-74515-w

**Published:** 2026-06-26

**Authors:** Ngoc-Huynh Ho, Sokratis Charisis, Nicolas Honnorat, Sachintha Ransara Brandigampala, Di Wang, Susan R. Heckbert, Peter T. Fox, David Martinez, David H. Wang, Timothy M. Hughes, Derek B. Archer, Timothy J. Hohman, Sudha Seshadri, Christos Davatzikos, Mohamad Habes

**Affiliations:** 1 Glenn Biggs Institute for Neurodegenerative Disorders, Neuroimage Analytics Laboratory and Biggs Institute Neuroimaging Core, University of Texas Health Science Center at San Antonio, San Antonio, TX USA; 2 Department of Epidemiology, University of Washington, Seattle, WA USA; 3 Research Imaging Institute, University of Texas Health Science Center at San Antonio, San Antonio, TX USA; 4 Gerontology and Geriatric Medicine, Wake Forest University School of Medicine, Winston-Salem, NC USA; 5 Vanderbilt Memory and Alzheimer’s Center, Vanderbilt University Medical Center, Nashville, TN USA; 6 Department of Radiology, University of Pennsylvania, Philadelphia, PA USA

**Keywords:** Alzheimer's disease, Learning algorithms, Computational models

## Abstract

Dementia, a degenerative disease affecting millions globally, is projected to triple by 2050. Early and precise diagnosis is essential for effective treatment and improved quality of life. However, current diagnostic approaches often show inconsistent performance across multi-racial and multi-ethnic groups, raising concerns about fairness and clinical reliability. This study investigates performance discrepancies in dementia classification among 6584 Non-Hispanic White, 1263 Non-Hispanic African American, and 713 Hispanic White populations. We observed significant cross-group bias, particularly when models trained on one group are tested on another. To address this, we evaluated RegAlign, a few-shot domain adaptation objective that combines source-side focal learning, target-side class-weighted supervision, and class-conditional alignment to improve adaptation to underrepresented populations. Our results show that this approach substantially reduces inter-group performance gaps, especially between Non-Hispanic White and Hispanic populations. Here, we show the importance of fairness-aware learning strategies and diverse training data for improving the accuracy and equity of MRI-based dementia classification.

## Abstract (web summary)

This study shows that MRI-based dementia classifiers can perform unevenly across racial and ethnic groups, and that fairness-aware adaptation can reduce diagnostic performance gaps.

## Introduction

Dementia is a clinical condition characterized by a gradual decline in cognitive function that affects memory, reasoning, communication, and the ability to perform daily tasks. This decline is typically the result of brain cell damage from conditions such as Alzheimer’s disease (AD)^1–5^. Early diagnosis can play a crucial role in customizing treatment plans and managing the disease more effectively^6–8^. In recent years, artificial intelligence (AI) has shown promising potential in improving the accuracy of dementia diagnoses by applying advanced machine learning (ML) techniques to medical imaging data, including magnetic resonance imaging (MRI). These models are capable of detecting subtle, often subclinical, structural brain changes, enabling earlier and more reliable diagnoses than traditional clinical approaches^9–12^.

Nevertheless, AI-driven diagnostic systems may not generalize equally across racial and ethnic groups.^13–18^. In dementia classification, such differences may arise from unequal representation in training data, population-level variation in brain structure and disease expression, and broader clinical and social determinants of health.^19–21^. These factors can lead to underdiagnosis or overdiagnosis^22,23^, and may amplify existing healthcare disparities. A prior epidemiologic study^24^ reported significant variations in dementia incidence across six population groups over 14 years. Similarly, Gilsanz et al.^25^ explored dementia rates in individuals aged 90 and above in a broadly representative cohort, offering insight into how risk varies across diverse backgrounds.

Prior work has also shown that racial and ethnic differences in Alzheimer’s disease risk, biomarker associations, and disease presentation may influence both predictive modeling and interpretation. For example, although the *APOE*-*ε*4 allele is a recognized risk factor among individuals of White descent^15,26,27^, African American and Hispanic populations have been reported to exhibit elevated Alzheimer’s risk regardless of *APOE* genotype^28,29^. In addition, ancestry-related genetic and epigenetic variation, shaped by social and environmental exposures, may influence brain morphology and dementia pathology across populations^17,26–28^. For machine learning models, such variation can alter the meaning of imaging-derived features, introduce hidden confounding, and reduce the transferability of attribution patterns across groups^18,19^. Consistent with this concern, prior work has noted that differences in brain morphology, socioeconomic conditions, and access to care may contribute to these disparities^27^, and models trained predominantly on White American populations often show reduced accuracy in other groups^30^. More broadly, race-neutral healthcare algorithms can still perpetuate diagnostic imbalance^31^; for example, higher false-positive rates have been reported for Hispanic and Asian patients in colorectal cancer prediction models that did not account for ethnicity^32^.

In this work, we explore diagnostic discrepancies in dementia classification by analyzing MRI-derived features from White American, African American, and Hispanic populations. ML models were applied to data from 9,007 participants (summarized in Table 1) to identify discrepancies in diagnostic outcomes. To address these issues, we evaluated several discrepancy mitigation strategies, including data harmonization, correlation removal, kernel mean matching, semi-supervised domain adaptation, and the proposed RegAlign. Unlike the comparator methods, RegAlign was designed for few-shot labeled target adaptation in clinically imbalanced settings by jointly combining source-side focal learning, target-side class-weighted supervision, and class-conditional alignment. These approaches were evaluated for their ability to improve diagnostic accuracy while reducing inter-group performance disparities. We also used SHAP- and ALE-guided interpretation analyses to examine the regional brain patterns associated with inter-group performance discrepancies. This work highlights the importance of heterogeneity in model training and discrepancy mitigation strategies in developing fair, representative, and reliable AI applications for dementia diagnosis and care across diverse population groups.

**Table 1:** Demographic and clinical characteristics by race/ethnicity

Variable	Overall (*n* = 9007)	NHW(*n* = 6584)	NHA (*n* = 1263)	HISP (*n* = 713)	Other (*n* = 447)	Test Statistic (p)
NACC/SCAN						
Diagnosis (CN/DA)	4834/1842	3487/1490	839/147	311/142	197/63	*χ*^2^(3) = 97.77, *p* = 4.70*e*^−21^*V* = 0.121, 95% CI [0.103, 0.142]
Age (years)						
CN	70.5 ± 10.0	70.5 ± 10.5	70.6 ± 8.0	70.5 ± 9.9	68.8 ± 8.4	*F*(3, 4830) = 1.89, *p* = 0.13*η*^2^ = 0.001, 95% CI [0.000, 0.004]
DA	73.0 ± 9.9	72.7 ± 9.8	75.3 ± 9.1	72.9 ± 11.8	74.9 ± 9.3	*F*(3, 1838) = 4.07, *p* = 6.84*e*^−3^*η*^2^ = 0.007, 95% CI [0.002, 0.016]
Female, %	62%	58%	75%	67%	67%	*χ*^2^(3) = 106.81, *p* = 5.32*e*^−23^*V* = 0.126, 95% CI [0.106, 0.149]
Education (years)						
CN	16.2 ± 4.0	16.6 ± 3.1	15.4 ± 3.9	14.0 ± 6.5	16.2 ± 9.1	*F*(3, 4830) = 55.52, *p* = 2.81*e*^−35^*η*^2^ = 0.033, 95% CI [0.017, 0.063]
DA	15.3 ± 5.2	15.7 ± 3.6	15.2 ± 10.3	11.8 ± 5.1	14.5 ± 12.1	*F*(3, 1838) = 25.75, *p* = 2.85^−16^*η*^2^ = 0.040, 95% CI [0.023, 0.101]
MMSE						
CN	28.7 ± 1.6	29.0 ± 1.3	27.9 ± 2.1	27.9 ± 1.9	28.1 ± 2.0	*F*(3, 1045) = 36.28, *p* = 2.64*e*^−22^*η*^2^ = 0.094, 95% CI [0.061, 0.140]
DA	20.4 ± 5.9	21.0 ± 5.6	18.2 ± 6.7	18.3 ± 6.6	18.4 ± 5.4	*F*(3, 882) = 11.33, *p* = 2.69*e*^−7^*η*^2^ = 0.037, 95% CI [0.018, 0.070]
ADNI/STAC						
Diagnosis (CN/DA)	1389/942	860/747	231/46	185/75	113/74	*χ*^2^(3) = 104.29, *p* = 1.86*e*^−22^*V* = 0.212, 95% CI [0.180, 0.248]
Age (years)						
CN	70.5 ± 7.4	71.8 ± 6.8	67.6 ± 7.3	69.1 ± 8.2	68.9 ± 7.6	*F*(3, 1385) = 25.86, *p* = 2.81*e*^−16^*η*^2^ = 0.053, 95% CI [0.034, 0.080]
DA	74.5 ± 7.6	74.6 ± 7.6	70.8 ± 7.1	74.5 ± 8.4	75.9 ± 6.5	*F*(3, 938) = 4.61, *p* = 3.29*e*^−3^*η*^2^ = 0.015, 95% CI [0.005, 0.035]
Female, %	56%	51%	73%	67%	56%	*χ*^2^(3) = 61.71, *p* = 2.53*e*^−13^1*V* = 0.163, 95% CI [0.126, 0.202]
Education (years)						
CN	16.4 ± 3.4	16.6 ± 2.5	15.9 ± 2.6	15.8 ± 6.9	17.0 ± 2.2	*F*(3, 1384) = 5.53, *p*9.03*e*^−4^*η*^2^ = 0.012, 95% CI [0.003, 0.052]
DA	15.4 ± 3.0	15.7 ± 2.7	14.2 ± 3.1	13.4 ± 3.5	15.0 ± 4.1	*F*(3, 933) = 16.37, *p*2.25*e*^−10^*η*^2^ = 0.050, 95% CI [0.027, 0.090]
MMSE						
CN	29.0 ± 1.3	29.1 ± 1.2	28.7 ± 1.3	29.0 ± 1.3	28.8 ± 1.3	*F*(3, 1216) = 4.42, *p* = 4.22*e*^−3^*η*^2^ = 0.011, 95% CI [0.003, 0.029]
DA	20.8 ± 5.1	20.9 ± 5.2	21.2 ± 4.0	21.0 ± 4.7	20.2 ± 5.3	*F*(3, 822) = 0.46, *p* = 0.71*η*^2^ = 0.002, 95% CI [0.000, 0.015]

No adjustment was applied for multiple comparisons.

*NHW* non-Hispanic White, *NHA* non-Hispanic African, *HISP* Hispanic, *CN* cognitively normal, *DA* Dementia of Alzheimer’s type, *MMSE* Mini-Mental State Examination.

## Results

### Performance discrepancies across multi-racial and multi-ethnic groups

In this section, we first evaluated whether performance discrepancies persisted after controlling for major confounders. To do so, we constructed a strictly group-balanced subset from the NACC and SCAN cohorts, matched by diagnosis, age, and sex across NHW, NHA, and HISP participants. This design allowed us to isolate race/ethnicity-associated differences in model behavior while minimizing the effects of sample imbalance, demographic mismatch, and site-related variability.

*Baseline Evidence of Inter-group Performance Discrepancy*. To evaluate the presence of performance discrepancies across racial and ethnic populations in dementia classification while minimizing potential confounding effects, we constructed a strictly group-balanced subset stratified by diagnosis and race/ethnicity from NACC and SCAN cohorts. As shown in Table 2, the cohort consisted of 426 CN and 426 DA participants, with equal representation from NHW, NHA, and HISP groups (142 CN and 142 DA per race/ethnicity). The sample size per group was determined by the number of available Hispanic DA subjects, ensuring identical diagnostic and racial and ethnic composition across all groups.

**Table 2:** Group-balanced set stratified by diagnostic group and race/ethnicity

Variable	Overall	NHW	NHA	HISP	Test
Diagnosis (CN/DA)	426/426	142/142	142/142	142/142	*χ*^2^(2) = 0.00, *p* = 1*V* = 0.00, 95% CI [0.01, 0.10]
AGE (years)					
CN	72.95 ± 6.63	72.92 ± 6.98	73.39 ± 5.68	72.53 ± 7.15	*F*(2, 423) = 0.59, *p* = 0.55*η*^2^ = 0.00, 95% CI [0.00, 0.03]
DA	73.57 ± 10.96	72.88 ± 11.78	74.95 ± 9.05	72.88 ± 11.76	*F*(2, 423) = 1.68, *p* = 0.19*η*^2^ = 0.01, 95% CI [0.00, 0.03]
t-test *pd* 95% CI	*t*(700) = − 1.00 0.32 − 0.07[ − 0.21, 0.07]	*t*(229) = 0.03 0.97 0.00 [ − 0.23, 0.24]	*t*(237) = − 1.74 0.08 − 0.21[ − 0.45, 0.02]	*t*(233) = − 0.30 0.76 − 0.04[ − 0.28, 0.20]	
Female, %					
CN	64.08%	64.08%	64.08%	64.08%	*χ*^2^(2) = 0.00, *p* = 1*V* = 0.00, 95% CI [0.01, 0.13]
DA	64.08%	64.08%	64.08%	64.08%	*χ*^2^(2) = 0.00, *p* = 1*V* = 0.00, 95% CI [0.01, 0.13]
Fisher’s exact *p* Odds ratio 95% CI	1 1 [0.76, 1.32]	1 1 [0.62, 1.62]	1 1 [0.62, 1.62]	1 1 [0.62, 1.62]	

*t*-tests and Fisher’s exact tests are two-sided. No adjustment was applied for multiple comparisons.

*NHW* non-Hispanic White, *NHA* non-Hispanic African, *HISP* Hispanic, *CN* cognitively normal, *DA* Dementia of Alzheimer’s type.

Participants were matched by age and gender both across racial and ethnic groups and between CN and DA within each group. No statistically significant differences were observed in age between racial and ethnic groups for either CN (ANOVA, *p* = 0.55) or DA (ANOVA, *p* = 0.19), nor between CN and DA within each group (all *t*-tests, *p*≥0.08). Gender distributions were identical across racial and ethnic and diagnostic groups (all *χ*^2^ and Fisher’s exact tests, *p* = 1), confirming demographic equivalence across all comparisons. The remaining NACC and SCAN partitions, together with the full ADNI and STAC cohorts, were used as an external validation set.

All neuroimaging features were harmonized using Combat-GAM to reduce site- and demographic-related variability while preserving biologically relevant variation. Harmonization was performed by aligning CN distributions across racial and ethnic groups and subsequently applying the learned parameters to DA participants. A comparison of baseline performance before and after harmonization is reported in Supplementary Fig. 2. All results presented below are based on harmonized data.

**Fig. 1:**
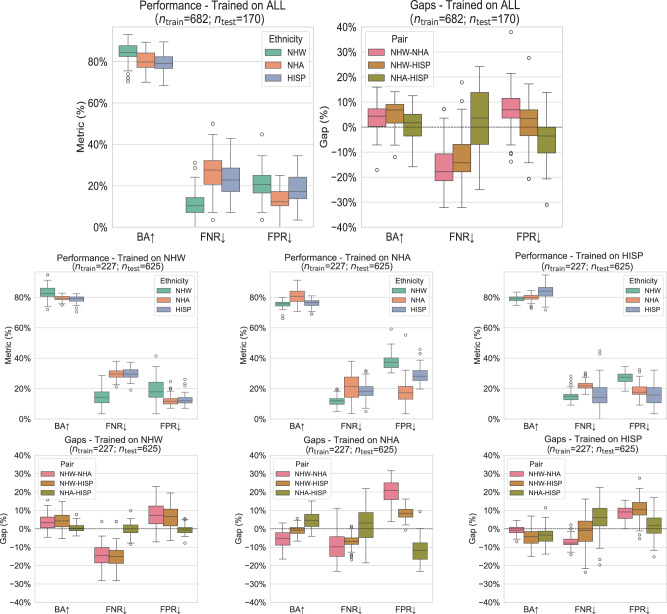
Baseline performance on group-balanced subsets for assessing disparities across multi-racial and multi-ethnic populations. The first row shows models trained on ALL groups, where left panel shows race/ethnicity-stratified performance metrics, while right panel shows pairwise gaps between groups, where zero dotted line indicates perfect agreement (no discrepancy). The second row shows performance of models trained on NHW (non-Hispanic White), NHA (non-Hispanic African) and HISP (Hispanic) populations only; and the third row shows results of pairwise gaps. For each box plot, the centre line denotes the median, the lower and upper box bounds denote the 25th and 75th percentiles, respectively, and whiskers extend to the most extreme non-outlier values within 1.5 times the interquartile range from the box bounds. Points beyond the whiskers, where shown, are outliers. The unit of study for the underlying evaluation data is one participant MRI scan with a diagnostic label (CN or DA) and race/ethnicity label. Number of samples *n*_*train*_ and *n*_*test*_ are counted per run. BA: balanced accuracy; FNR: false negative rate; FPR: false positive rate; *↑*: higher is better; *↓*: lower is better. Source data are provided as Source Data 1, including minima, maxima, and outliers.

Figure 1 summarizes baseline model performance and pairwise racial/ethnic discrepancies on the harmonized, group-balanced subset across four training scenarios. When trained on all groups, the model achieved its highest BA for NHW participants (84.51%). Ethnicity-specific training resulted in the highest BA consistently occurring within the same racial/ethnic group as the training population: 83.08% for NHW when trained on NHW only, 80.84% for NHA when trained on NHA only, and 83.71% for HISP when trained on HISP only. Across all training scenarios, NHW participants consistently showed the lowest FNR relative to NHA and HISP participants across training scenarios.

Pairwise gap analyses further quantified discrepancies between racial and ethnic groups. When trained exclusively on NHW participants, the model exhibited large FNR gaps for both NHW–NHA (14.65%) and NHW–HISP (14.91%) pairs. Training on NHA participants resulted in an increased FNR gap for the NHW–NHA pair (9.10%) and a comparatively higher FPR gap for the same pair (20.26%). When trained on HISP participants, an increased FPR gap was observed for the NHW–HISP pair (10.89%). Furthermore, even when trained on all ethnic groups jointly, FNR gaps remained evident for NHW–NHA (16.23%) and NHW–HISP (12.96%) comparisons. Similar inter-group discrepancies were also observed with alternative classifiers, including support vector machine (SVM) and multilayer perceptron (MLP), as shown in Supplementary Fig. 3.

Supplementary Table 3 report pairwise statistical tests of performance gaps across racial and ethnic groups under the group-balanced evaluation setting, including mean gaps, 95% confidence intervals, and two-sided *t*-tests for BA, FNR, and FPR. Across all training configurations, many pairwise comparisons exhibit confidence intervals that do not intersect zero and highly significant *p*-values (often *p* < 10^−8^), indicating systematic differences in both sensitivity and specificity across groups. Detailed results are presented in Supplementary Data 3 and Supplementary Data 4.

In general, these results demonstrate that measurable performance discrepancies across considered groups persist even after strict group balancing, demographic matching, and harmonization. The consistency of these gaps across training scenarios indicates that race/ethnicity-associated differences in model behavior remain under controlled conditions.

**Fig. 2:**
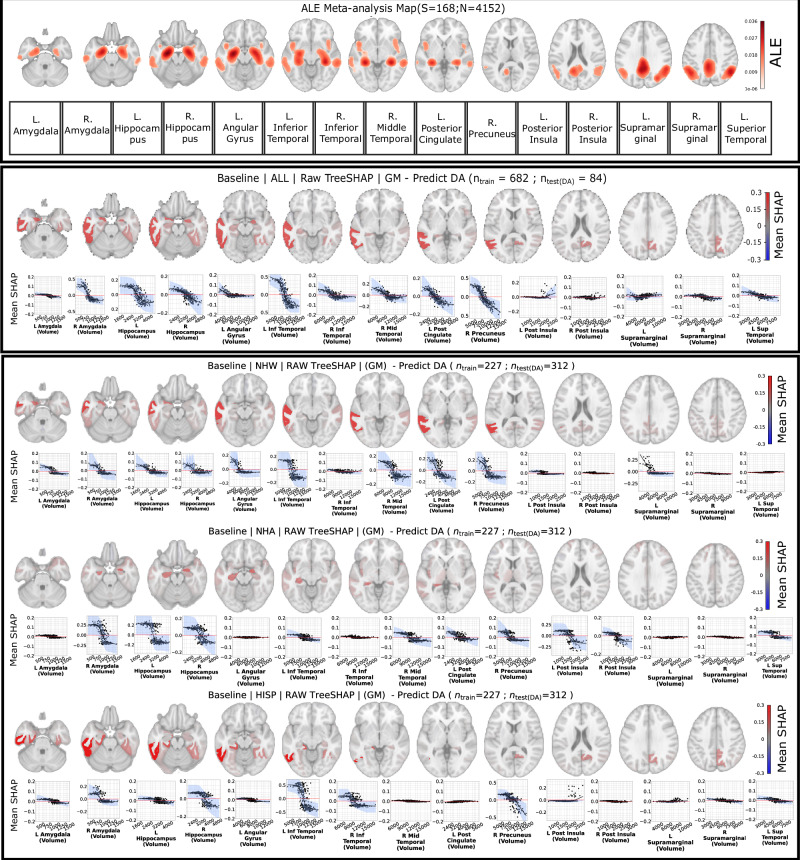
SHAP-based brain maps and partial dependence visualizations for the baseline dementia classifier under ALE-guided meta-analysis. This figure summarizes population-specific attribution patterns in the baseline models and highlights differences in learned regional contributions across training populations. The top panel shows the ALE meta-analysis map. The second row presents average SHAP-based brain maps over 100 runs and partial dependence plots for fifteen key regions from the baseline model trained on all populations combined. The third, fourth, and fifth rows show the corresponding results from baseline models trained separately on NHW, NHA, and HISP subjects, respectively. For each partial dependence plot, shaded error bands denote 95% confidence intervals around the binned mean SHAP values, calculated as mean +/- 1.96 standard errors within each regional volume bin. Here, *S*: the number of Voxel-based morphometry studies for meta-analysis, *N*: the number of subjects, ALE: activation likelihood estimation; *n*_train_: the number of training subjects, *n*_test (DA)_: the number of test DA (Dementia of Alzheimer’s type) subjects per run; NHW: non-Hispanic White, NHA: non-Hispanic African, HISP: Hispanic; GM: gray matter, L: left, R: right. Source data are provided as Source Data 7.

*Discrepancy Interpretation via TreeSHAP*. To interpret population-specific performance discrepancies observed under group-balanced evaluation, we analyzed feature-level attribution patterns using TreeSHAP combined with region-wise partial dependence analysis, as shown in Fig. 2. To focus on robust and decisive effects, we restricted interpretation to brain regions whose SHAP-based 95% confidence intervals did not intersect the zero attribution line, thereby excluding features with unstable or non-informative contributions.

The first panel highlights fifteen brain regions consistently identified through the ALE-based meta-analysis, serving as an independent, biologically grounded reference set. The ALE meta-analysis revealed the highest convergence in the left parahippocampal gyrus (Cluster 3), which showed the maximum ALE value (0.0360) and *z*-score (10.17), with peak coordinates at ( − 26,  − 12,  − 18). Additional prominent peaks included the left cingulate gyrus (ALE = 0.0329, *z* = 9.41; coordinates:  − 4,  − 50, 32), the right inferior parietal lobule (ALE = 0.0292, *z* = 8.47; coordinates: 50,  − 58, 42), and the right parahippocampal gyrus (ALE = 0.0291, *z* = 8.44; coordinates: 26,  − 10,  − 18). Further significant convergence was observed in the right parahippocampal gyrus at a more posterior location (ALE = 0.0291, *z* = 8.43; coordinates: 34,  − 34,  − 6), the left temporal sub-gyral region (ALE = 0.0282, *z* = 8.21; coordinates:  − 30,  − 34,  − 4), the left angular gyrus (ALE = 0.0252, *z* = 7.39; coordinates:  − 44,  − 60, 40), and the left inferior parietal lobule (ALE = 0.0244, *z* = 7.17; coordinates:  − 48,  − 54, 42). Together, these regions predominantly localize to medial temporal and parietal association cortices, consistent with established neurodegenerative patterns observed in dementia.

The second panel presents SHAP attribution maps for the model trained on all ethnic groups jointly. In this setting, salient contributions were observed in the left inferior temporal cortex, left middle temporal cortex, right amygdala, left entorhinal cortex, right precuneus, right fusiform gyrus, left hippocampus, and right frontal operculum. These regions largely overlap with canonical AD-related temporal and limbic association cortices.

The third panel contrasts attribution patterns for models trained exclusively within individual ethnic groups. When trained on NHW participants, the model emphasized bilateral middle temporal regions, left entorhinal cortex, left inferior temporal cortex, bilateral precuneus, left parahippocampal gyrus, and right hippocampus. In contrast, the NHA-trained model showed prominent attributions in the left posterior insula, left and right hippocampus, right amygdala, left middle temporal cortex, right temporal pole, and bilateral entorhinal cortex. The HISP-trained model highlighted the left inferior temporal cortex, left parahippocampal gyrus, bilateral fusiform gyrus, right precuneus, left entorhinal cortex, right amygdala, and right hippocampus. While partial overlap exists across groups, the relative prominence and spatial distribution of contributing regions varied substantially by training population.

Guided by the ALE-derived regions, we further examined fifteen partial dependence plots to assess how regional volumes modulated model predictions across three racial and ethnic groups. In particular, NHA participants exhibited stronger associations between SHAP scores and both right amygdala and left hippocampal volumes compared with NHW and HISP participants. Meanwhile, NHW and HISP groups demonstrated clear predictive dependence on left parahippocampal gyrus volume, whereas this relationship was largely absent in the NHA group. Supplementary Fig. 11 highlights population-specific attribution patterns across all MUSE-derived brain regions. These differential response patterns are consistent with the persistence of race/ethnicity-associated performance differences even after harmonization and strict group balancing. Together, these findings support the hypothesis that population-specific biological variation influences both disease presentation and the predictive patterns learned by the model.

### Performance on discrepancy mitigation techniques

**Fig. 3:**
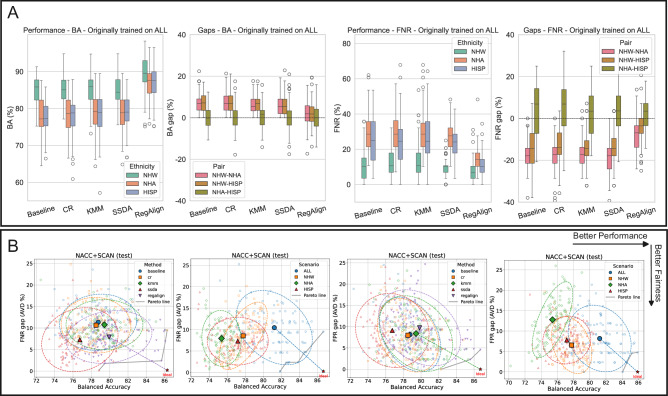
Comparison of discrepancy mitigation methods relative to baseline and trade-off between performance and fairness. **A** Comparison of ALL-trained model performance on group-balanced sets for evaluating disparity mitigation across racial and ethnic groups. Box plots summarize *n* = 100 independent model-training/evaluation replicates per plotted box, where each replicate is one complete model run evaluated on a held-out group-balanced subject subset of 170 subjects. These are computational/model-training replicates, not biological or technical measurement replicates. For each box plot, the centre line denotes the median, the lower and upper box bounds denote the 25th and 75th percentiles, respectively, and whiskers extend to the most extreme non-outlier values within 1.5 times the interquartile range from the box bounds. Points beyond the whiskers, where shown, are outliers. The unit of study for the underlying evaluation data is one participant MRI scan with a diagnostic label (CN or DA) and race/ethnicity label. BA: balanced accuracy; FNR: false negative rate; FPR: false positive rate; CR: Correlation Remover; KMM: Kernel Mean Matching; SSDA: Semi-Supervised Domain Adaptation. Source data are provided as Source Data 2a, including minima, maxima, and outliers. **B** Pareto-front analysis of the trade-off between performance and fairness in group-balancing test set (NACC+SCAN). Scatter plots show BA (%) versus average discrepancy (AvD, %), with lower AvD indicating smaller inter-group disparity. The red star marks the ideal point (high BA, low gap), and the gray curve indicates the Pareto (non-dominated) frontier. Each point corresponds to four-run bin. Large filled markers denote centroids, and dashed ellipses summarize the distribution of runs. Source data are provided as Source Data 2b.

This section evaluates model performance on dementia classification under different training group compositions detailed in the Supplementary Experimental Settings, and assesses methods for mitigating intergroup discrepancies in predictive accuracy and error rates. Figure 3A compares mean performance (left) and mean pairwise racial/ethnic gaps (right) across Baseline, CR, KMM, SSDA, and RegAlign on the group-balanced subset for ALL training settings, focusing on BA and FNR; all values are mean ± standard deviation (SD) across 100 runs.

When originally trained on ALL, mean BA for NHW was similar across methods, with CR yielding the highest mean NHW BA: 85.19% ± 3.48%, *p* = 0.546. In contrast, RegAlign achieved the highest mean BA for NHA and HISP: NHA 86.40% ± 4.40%, *p* < 0.001; HISP 87.79% ± 4.23%, *p* < 0.001. The corresponding Baseline BA means were NHA 77.94% ± 4.85% and HISP 77.68% ± 4.35%. For FNR, SSDA produced the lowest mean NHW FNR: 9.09% ± 5.15%, *p* = 0.014. RegAlign reduced mean FNR for NHA and HISP to 14.72% ± 7.10%, *p* < 0.001, and 11.13% ± 6.15%, *p* < 0.001, respectively, compared with Baseline NHA 30.00% ± 10.69%; HISP 25.88% ± 12.97%).

For pairwise gaps, RegAlign reduced BA gaps relative to Baseline for NHW–NHA (Baseline 6.93% ± 4.31% vs. RegAlign 1.40% ± 5.75%, *p* < 0.001) and NHW–HISP (Baseline 7.19% ± 4.14% vs. RegAlign 2.79% ± 5.35%, *p* < 0.001). Moreover, RegAlign reduced FNR gaps for NHW–NHA (Baseline 18.46% ± 7.38% vs. RegAlign 4.40% ± 7.09%, *p* < 0.001) and NHW–HISP (Baseline 14.34% ± 9.00% vs. RegAlign 0.81% ± 6.73%, *p* < 0.001).

For NHW-only training, RegAlign achieved the highest mean BA for all evaluated ethnic groups: NHW 84.67% ± 4.53%, *p* = 0.010; NHA 81.12% ± 1.89%, *p* < 0.001; HISP 82.20% ± 1.92%, *p* < 0.001. By comparison, the corresponding Baseline BA means were NHW 83.12% ± 3.86%; NHA 77.56% ± 3.22%; HISP 76.75% ± 3.76%. RegAlign also yielded the lowest mean FNR across ethnic groups: NHW 10.55% ± 6.99%; NHA 20.19% ± 5.33%; HISP 17.61% ± 5.03%, with all *p* < 0.001. The corresponding Baseline FNR means were NHW 17.88% ± 11.14%; NHA 34.13% ± 10.66%; HISP 35.72% ± 12.58%.

Furthermore, RegAlign reduced BA gaps relative to Baseline for NHW–NHA (Baseline 5.57% ± 3.43% vs. RegAlign 3.55% ± 5.05%, *p* = 0.001) and NHW–HISP (Baseline 6.37% ± 4.10% vs. RegAlign 2.47% ± 4.69%, *p* < 0.001). Similarly, RegAlign reduced FNR gaps relative to Baseline for NHW–NHA (Baseline 16.24% ± 3.89% vs. RegAlign 9.64% ± 6.09%, *p* < 0.001) and NHW–HISP (Baseline 17.84% ± 5.11% vs. RegAlign 7.06% ± 5.30%, *p* < 0.001).

Under NHA-only training, RegAlign improved mean BA and reduced mean FNR relative to Baseline across all evaluated groups (BA for NHA: 79.74% ± 3.95% to 85.22% ± 5.12%; FNR for NHA: 28.34% ± 9.92% to 13.40% ± 7.04%), with *p* < 0.001, while FNR gap magnitudes decreased for NHW–NHA (from 8.85% ± 9.25% to 2.74% ± 6.30%, *p* < 0.001) and NHW–HISP (from 9.06% ± 4.77% to 3.79% ± 2.61%, *p* < 0.001). For HISP-only training, RegAlign increased mean BA and reduced mean FNR across all races/ethnicities (BA for HISP: 83.49% ± 3.52% to 86.10% ± 4.08%; FNR for HISP: 17.63% ± 9.99% to 9.51% ± 5.57%), with *p* < 0.001, and reduced gap magnitudes for NHW–NHA BA (from 0.47% ± 2.19% to 0.31% ± 1.97%, *p* = 0.009) and NHW–NHA FNR (from 7.38% ± 2.80% to 4.79% ± 3.06%, *p* < 0.001). In contrast, NHW–HISP BA gaps were largely unchanged (from  − 5.27% ± 3.41% to  − 5.45% ± 4.18%, *p* = 0.742), while mean FPR increased for HISP (15.39% ± 11.13% to 18.29% ± 8.72%). Complete results for all experiments, including race/ethnicity-stratified performance and pairwise gap analyses, are reported in the Supplementary Performance on Discrepancy Mitigation Techniques.

Performance and discrepancy gaps of external validation can be found in Supplementary Performance on External Datasets as well as Supplementary Data 5 and Supplementary Data 6.

### Trade-off between accuracy and fairness

Figure 3 B summarizes the trade-off between classification performance and fairness by jointly plotting balanced accuracy (BA%; higher is better) against disparity measured as the gap (AVD%; lower is better) for both error types (FNR gap and FPR gap). Each method (model-based comparison) or training scenario (scenario-based comparison) is represented by a distribution of runs, summarized by a centroid marker and a confidence ellipse; the red star denotes the ideal point (high BA, low gap), and the gray Pareto line indicates the non-dominated frontier in this objective space. Across both the NACC+SCAN test set and the external evaluation (remaining NACC+SCAN and whole ADNI+STAC, as shown in Supplementary Fig. 6-8), the proposed RegAlign occupies the optimal region of the Pareto space relative to Baseline, CR, KMM, and SSDA, achieving the closest trade-off to the ideal point when considering BA jointly with either FNR gap or FPR gap.

Scenario-based comparisons further demonstrate that the optimal trade-off depends on the error type and evaluation setting. On the NACC+SCAN test set, training on ALL populations yields the most optimal trade-off for BA versus FPR gap, whereas training on HISP yields the most favorable trade-off for BA versus FNR gap, as shown in Fig. 3B. On the external evaluation, training on ALL populations provides the most optimal trade-off for BA versus FNR gap, while training on NHW provides the most favorable trade-off for BA versus FPR gap, as shown in Supplementary Fig. 8. In general, these Pareto analyses identify RegAlign as the most Pareto-efficient method across datasets when balancing BA against both types of discrepancies, FNR and FPR gaps.

## Discussion

This study examined fairness and interpretability in MRI-based dementia classification across multi-racial and multi-ethnic populations. Using multi-cohort data (6676 NACC/SCAN subjects with external validation in 2331 ADNI/STAC subjects), we show that race/ethnicity-associated performance differences are not explained solely by sample imbalance or site effects, showing that inter-group performance disparities persist even after strict group balancing, demographic matching, and Combat-GAM harmonization (Results: Performance Discrepancies across Multi-Racial and Multi-Ethnic Groups). Across training scenarios, models tended to perform best within the population used for training, while cross-population application produced systematic shifts in sensitivity and specificity, underscoring the risk of deploying single-population models in heterogeneous clinical settings.

To mitigate these disparities, we benchmarked correlation removal, kernel mean matching, semi-supervised domain adaptation, and the proposed RegAlign. RegAlign is a regularized few-shot adaptation objective designed for clinically imbalanced settings. Its contribution is not simply that it aligns source and target data, but that it jointly addresses three challenges common in underrepresented populations: source-domain imbalance, sparse labeled target samples, and cross-group prediction mismatch. In this respect, RegAlign differs from feature-level correction methods such as correlation remover, reweighting-based approaches such as kernel mean matching, and entropy-based semi-supervised adaptation on unlabeled target data. Empirically, it provided the most consistent improvement in the balance between predictive performance and inter-group parity across training configurations (Results: Trade-off Between Accuracy and Fairness).

Interpretability analyses further contextualized the observed performance differences. TreeSHAP attribution maps and ALE-guided comparisons indicated both shared and population-specific patterns of regional importance, with differential dependence relationships across racial and ethnic groups in canonical AD-related regions (e.g., medial temporal and limbic structures). The persistence of these attribution differences under harmonized, group-balanced evaluation suggests that models may leverage partially distinct structural signatures across populations, which can lead to differences in error rates across groups and reduce how well explanations generalize from one population to another.

Several limitations should be considered. First, minoritized groups remain smaller than NHW in the full dataset, and scarcity can affect both model stability and attribution robustness despite harmonization and repeated resampling. Second, we focused on structural MRI and MUSE-derived ROI features; hence, extending to multimodal biomarkers, longitudinal trajectories, and additional social/clinical covariates may refine both fairness characterization and mechanistic interpretation. Finally, fairness was primarily quantified via pairwise error-rate gaps; therefore, future work should incorporate complementary criteria (e.g., calibration, decision-curve analyses, subgroup prevalence shifts) and evaluate downstream clinical impact. Overall, our results indicate that fairness-aware learning objectives such as RegAlign, combined with diverse training data and interpretable analyses, are important for developing fair and generalizable dementia classification models across multi racial and multi-ethnic populations.

## Methods

This study was conducted in accordance with all relevant ethical regulations. The use of de-identified data from the participating cohorts was approved by the relevant institutional review boards and data-use committees at the contributing institutions. The present secondary analysis was performed using de-identified data obtained under approved data-use agreements. Informed consent was obtained from participants by the original cohort studies according to their respective protocols. No participant compensation was provided as part of the present secondary analysis.

**Fig. 4:**
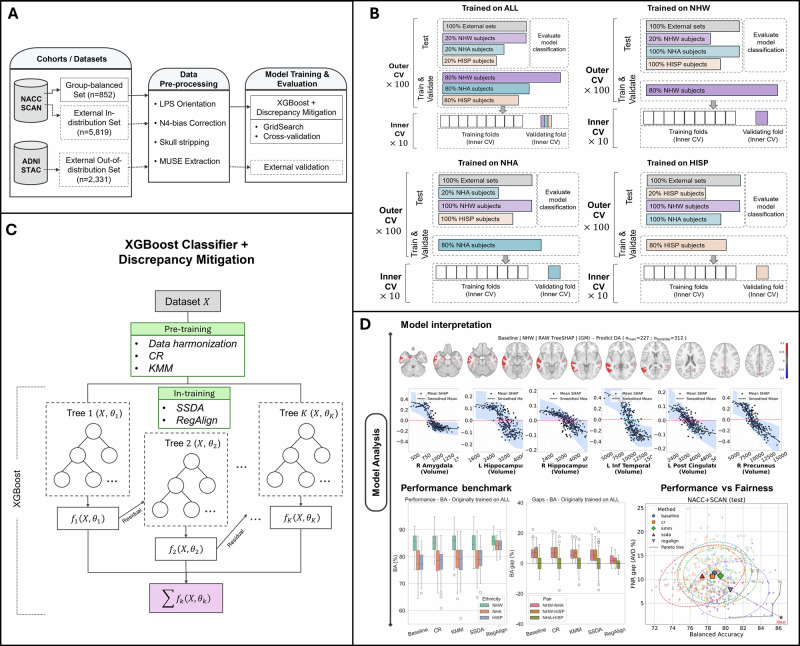
Overview of the proposed machine learning framework for fair dementia classification. **A** The study uses MRI data from multiple cohorts with diverse racial and ethnic representation, including the NACC/SCAN cohorts (group-balanced and out-of-sample sets) and ADNI/STAC (external set). The pipeline integrates preprocessing, domain-aware training with a customized loss (RegAlign), and interpretable evaluation using SHAP-based brain region importance. **B** Scenario-specific strategy for training/test splitting. **C** Visualization of the XGBoost classifier combined with the customized loss function, RegAlign. **D** Evaluation includes performance metrics across racial and ethnic subgroups, fairness assessments via Pareto analysis, and visualization of feature contributions across racial and ethnic groups.

The proposed framework consists of three main components. (a) First, T1-weighted MRI scans were preprocessed through reorientation, bias correction, skull stripping, and MUSE^33^-based (Multi-Atlas Region Segmentation using Ensembles) region extraction. Second, dementia classification was performed using XGBoost combined with one of several discrepancy mitigation strategies. Third, model performance, inter-group discrepancy, and feature attribution patterns were evaluated across multiple training scenarios and external datasets. The detailed training procedures are provided in the Supplementary Experimental Settings section. A schematic overview is shown in Fig. 4. All analyses were performed using Python 3.11. The main software packages used in this study included NumPy v1.26.4 and pandas v2.3.1 for data processing, scikit-learn v1.7.1 for machine-learning utilities and model evaluation, XGBoost v3.0.0 for gradient-boosted tree classification, nibabel v5.3.2 and nilearn v0.13.1 for neuroimaging data handling, and matplotlib v3.10.0 and seaborn v0.13.2 for visualization.

### Study populations

Participants were obtained from multiple Alzheimer’s disease research cohorts to improve generalizability and enable external validation. The primary dataset was derived from the National Alzheimer’s Coordinating Center (NACC; https://www.alz.washington.edu/), which aggregates standardized clinical and neuroimaging data from Alzheimer’s Disease Centers (ADCs) across the United States ^34^. We additionally included participants from the SCAN cohort (https://scan.naccdata.org/). For external validation, we incorporated two independent cohorts: the Alzheimer’s Disease Neuroimaging Initiative (ADNI; https://adni.loni.usc.edu/) and the South Texas Alzheimer’s Disease Research Center (STAC). These cohorts differ in recruitment strategies, imaging protocols, and population composition, thereby enabling evaluation of model robustness under real-world distributional shifts.

Participant age and sex were available from the source cohort metadata and are summarized in Table 1. Sex was recorded in the source datasets using cohort-provided variables, which were derived from participant-reported or cohort-recorded information according to each cohort’s original data collection protocol. Sex was included as a demographic variable and was used for descriptive characterization and group matching where applicable. Sex-disaggregated demographic summaries are provided in Table 1 and Table 2.

Race and ethnicity were included in this study because the primary objective was to evaluate inter-group differences in model performance and fairness across socially defined population groups, rather than to treat race/ethnicity as biological proxies. In the source cohorts, race and ethnicity were recorded as separate variables using participant-reported or cohort-recorded classification fields. Race categories included White, African American/Black, Asian American, Native Hawaiian, Alaska Native, American Indian, and unknown, and ethnicity was recorded as Hispanic or non-Hispanic. For the present analysis, these variables were combined to define three primary groups: Non-Hispanic White (NHW), Non-Hispanic African American (NHA), and Hispanic White (HISP). Due to limited sample sizes in the remaining categories, participants identified as Asian American, Native Hawaiian, Alaska Native, American Indian, or unknown were grouped into an aggregated *Other* category. In total, the dataset comprised 9007 participants, including 6584 NHW (4347 cognitively normal [CN], 2237 Dementia of Alzheimer’s type [DA]), 1263 NHA (1070 CN, 193 DA), and 713 HISP (496 CN, 217 DA) individuals, and the aggregated *Other* category included 310 CN and 137 DA participants. Table 1 provides detailed demographic and clinical characteristics for all cohorts, including age, sex, education, and Mini-Mental State Examination (MMSE) scores, stratified by diagnosis and racial and ethnic group.

### Image processing

The study utilized raw T1-weighted MRI data in DICOM format from the NACC, SCAN, ADNI, and STAC cohorts to facilitate an in-depth investigation of brain regions of interest (ROIs). We first reoriented the images according to the LPS (Left-Posterior-Superior) convention to standardize their spatial orientation. N4-bias field correction^35^ was employed to reduce intensity inhomogeneity across the images. Next, we performed skull stripping and brain segmentation using Multi-Atlas Region Segmentation using Ensembles (MUSE)^33,36^. MUSE generated 144 regional volumetric features used as model inputs for dementia classification. These features were subsequently used for dementia classification, supporting cross-group comparisons of brain atrophy patterns among different racial and ethnic populations^37^.

### ML classifier and discrepancy mitigation methods

In this study, we used XGBoost (XGB), a gradient-boosting technique recognized for its ability to manage imbalanced datasets^38–40^, as a baseline classifier for dementia classification. We trained the model to distinguish between CN and DA classes utilizing MUSE-based brain ROI extraction from MRI scans. These features capture structural variations across 144 anatomical regions and serve as a biologically informed input representation for classification. XGBoost was selected because it performs well on structured, potentially imbalanced data, captures non-linear interactions among brain regions, and supports feature-level interpretation.

*Conventional Approaches for Discrepancy Mitigation*. To address inter-group performance discrepancies in dementia classification, we implemented four bias mitigation strategies, including correlation remover, kernel mean matching, semi-supervised domain adaptation, and the proposed RegAlign. Data harmonization was performed via Combat-GAM^41–43^, a technique that compensates for site-specific and demographic factors (such as age, gender, race/ethnicity, and diagnosis) to mitigate batch effects while preserving biologically meaningful variation across multi-site MRI data^44,45^.

*Correlation Remover (CR)*^46–48^ algorithm uses a linear transformation to remove correlations between sensitive features (e.g., race/ethnicity) and non-sensitive features (e.g., MRI-derived brain regions). CR ensures, therefore, that the sensitive features do not influence the non-sensitive ones. Formally, let the dataset **X** consist of two subsets: sensitive features **S** and non-sensitive features **Z**. Let *m*_*s*_ and *m*_*z*_ denote the number of sensitive and non-sensitive features, respectively. The mean of the sensitive features is defined as $\overline{{{\bf{S}}}}={[{\overline{s}}_{1},\ldots,{\overline{s}}_{{m}_{s}}]}^{\top }$, where ${\overline{s}}_{j}$ is the mean of the *j*-th sensitive feature. For each non-sensitive feature ${{{\bf{z}}}}_{j}\in {{\mathbb{R}}}^{n}$, where *j* = 1, …, *m*_*z*_, an optimal weight vector ${{{\bf{w}}}}_{j}^{*}\in {{\mathbb{R}}}^{{m}_{s}}$ is calculated by minimizing the least squares objective:

1 $${\min }_{{{\bf{w}}}}\parallel {{{\bf{z}}}}_{j}-({{\bf{S}}}-{{{\bf{1}}}}_{n}\times {\overline{{{\bf{S}}}}}^{\top }){{\bf{w}}}{\parallel }_{2}^{2}.$$

Here, **1**_*n*_ is a vector of ones in ${{\mathbb{R}}}^{n}$. In this formulation, ${{{\bf{w}}}}_{j}^{*}$ represents the solution to a linear regression problem where the non-sensitive feature **z**_*j*_ is projected onto the sensitive features, which are mean-centered. By solving this problem for all non-sensitive features, the weight matrix ${{{\bf{W}}}}^{*}=[{{{\bf{w}}}}_{1}^{*},\ldots,{{{\bf{w}}}}_{{m}_{z}}^{*}]$ is derived. Using this weight matrix, the adjusted non-sensitive features Z^*^ is computed as:

2 $${{{\bf{Z}}}}^{*}={{\bf{Z}}}-({{\bf{S}}}-{{{\bf{1}}}}_{n}\times {\overline{{{\bf{S}}}}}^{\top }){{{\bf{W}}}}^{*}.$$

Finally, the sensitive features **S** are removed from the dataset **X**, and Z^*^ replaces the original non-sensitive features. A hyperparameter *α* (where *α* = 0.05) is introduced to allow flexibility in the degree of filtering applied, defined as:

3 $${{{\bf{X}}}}_{{{\rm{final}}}}=\alpha {{{\bf{Z}}}}^{*}+(1-\alpha ){{\bf{X}}}$$

Besides, the domain adaptation technique^49^ was applied using *Kernel Mean Matching (KMM)*^50,51^ to further mitigate bias. KMM is designed to address the problem of distribution mismatch between source and target domains in machine learning tasks. It reweights the source domain samples to more closely align with the target domain’s distribution in a reproducing kernel Hilbert space (RKHS)^52^, ensuring that the model performs well across different racial and ethnic groups. Let *ϕ*(*x*) be a feature mapping to a RKHS ${{\mathcal{H}}}$. The objective is to find weights *β* that minimize:

4 $${\min }_{\beta }{\left|\left| \frac{1}{n}\mathop{\sum }\limits_{i=1}^{n}{\beta }_{i}\phi ({x}_{i})-\frac{1}{m}\mathop{\sum }\limits_{j=1}^{m}\phi (x{\prime} )\right|\right| }_{{{\mathcal{H}}}}^{2},$$

while ensuring the following conditions are met:

5 $${\beta }_{i}\in [0,B]\,\,{{\rm{and}}}\,\,\left| \frac{1}{n}\mathop{\sum }\limits_{i=1}^{n}{\beta }_{i}-1\right| \le \epsilon,$$

where *x*_*i*_ and $x{\prime}$ are source and target domain samples, *n* and *m* are the number of source and target samples, *B* is the upper bound on the weights and was set to 512, and *ϵ* controls the discrepancy between the mean weights and 1, and is computed as $\epsilon=(\sqrt{n}-1)/\sqrt{n}$. The default radial basis function (RBF) kernel is utilized. The estimated sample weights were normalized to sum to 1 and then incorporated into classifier training. In this way, higher-weighted source samples contributed more strongly to the optimization objective, whereas lower-weighted samples had less influence.

The *semi-supervised domain adaptation (SSDA)* baseline combines supervised learning on the source domain with entropy minimization^53^ and class-conditional alignment on unlabeled target data. Let ${{{\mathcal{D}}}}_{S}={\{({x}_{i}^{S},{y}_{i}^{S})\}}_{i=1}^{n}$ be the labeled source set and ${{{\mathcal{D}}}}_{T}={\{{x}_{j}^{T}\}}_{j=1}^{m}$ be the unlabeled target set, where ${x}_{i}^{S}$ and ${x}_{j}^{T}$ are input samples, ${y}_{i}^{S}\in \{0,1\}$ is the source label, and ${\widehat{p}}_{i}^{S}={f}_{\theta }({x}_{i}^{S})$, ${\widehat{p}}_{j}^{T}={f}_{\theta }({x}_{j}^{T})$ are model-predicted probabilities under parameters *θ*.

(1) Source supervised classification loss (with focal weighting):

6 $${{{\mathcal{L}}}}_{{{\rm{cls}}}}^{S}=\frac{1}{n}\mathop{\sum }\limits_{i=1}^{n}{\left(1-{p}_{t,i}^{S}\right)}^{{\gamma }_{S}}\,{{\rm{BCE}}}\left({\widehat{p}}_{i}^{S},{y}_{i}^{S}\right),\,{p}_{t,i}^{S}=\left\{\begin{array}{ll}{\widehat{p}}_{i}^{S},& {y}_{i}^{S}=1,\\ 1-{\widehat{p}}_{i}^{S},& {y}_{i}^{S}=0,\end{array}\right.$$

7 $${{\rm{BCE}}}\left({\widehat{p}}_{i}^{S},{y}_{i}^{S}\right)=-\left[{y}_{i}^{S}\log {\widehat{p}}_{i}^{S}+\left(1-{y}_{i}^{S}\right)\log \left(1-{\widehat{p}}_{i}^{S}\right)\right].$$

Here, *γ*_*S*_≥0 is the source focal parameter. When *γ*_*S*_ = 0, this term reduces to standard BCE.

(2) Target entropy minimization loss

8 $${{{\mathcal{L}}}}_{{{\rm{ent}}}}^{T}=-\frac{1}{m}\mathop{\sum }\limits_{j=1}^{m}\left[{\widehat{p}}_{j}^{T}\log {\widehat{p}}_{j}^{T}+\left(1-{\widehat{p}}_{j}^{T}\right)\log \left(1-{\widehat{p}}_{j}^{T}\right)\right].$$

Here, *m* is the number of target samples; ${\widehat{p}}_{j}^{T}$ is probability of the predicted class-1 for sample *j*.

(3) Class-conditional alignment with pseudo-labels Pseudo-labels ${\widetilde{y}}_{j}^{T}\in \{0,1\}$ for target samples are obtained from target predictions (e.g., K-means on prediction scores). We define class index sets ${S}_{c}=\{i\,| \,{y}_{i}^{S}=c\}$ and ${T}_{c}=\{j\,| \,{\widetilde{y}}_{j}^{T}=c\}$ for *c* ∈ {0, 1}. Class-conditional means are

9 $${\mu }_{S}^{(c)}=\frac{1}{| {S}_{c}| }\mathop{\sum }\limits_{i\in {S}_{c}}{\widehat{p}}_{i}^{S},\,{\mu }_{T}^{(c)}=\frac{1}{| {T}_{c}| }\mathop{\sum }\limits_{j\in {T}_{c}}{\widehat{p}}_{j}^{T},$$

where ∣*S*_*c*_∣ and ∣*T*_*c*_∣ denote the number of samples in class *c* for source and pseudo-labeled target, respectively. The alignment loss is

10 $${{{\mathcal{L}}}}_{{{\rm{align}}}}^{S\leftrightarrow T}=\mathop{\sum }\limits_{c=0}^{1}{\left({\mu }_{T}^{(c)}-{\mu }_{S}^{(c)}\right)}^{2}.$$

(4) SSDA final objective is

11 $${{{\mathcal{L}}}}_{{{\rm{SSDA}}}}={{{\mathcal{L}}}}_{{{\rm{cls}}}}^{S}+\tau \,{{{\mathcal{L}}}}_{{{\rm{ent}}}}^{T}+\lambda \,{{{\mathcal{L}}}}_{{{\rm{align}}}}^{S\leftrightarrow T}.$$

Here, *τ*≥0 weights the target entropy term and *λ*≥0 weights the alignment term.

*Proposed RegAlign*. In typical domain adaptation settings, training on a source population using standard loss functions (e.g., cross-entropy or focal loss) often leads to reduced performance on underrepresented target populations, particularly when only a few labeled samples from the target domain are available. These issues are exacerbated when the source domain (e.g., African American or Hispanic cohorts) exhibits class imbalance, which causes models to overfit to the majority class (often normal cognition, NC) and underperform on the minority class (dementia). To address these challenges, we propose a custom loss function, termed *RegAlign*, which integrates three key components: (1) focal weighting to emphasize hard-to-classify samples within the source domain, (2) class-weighted binary cross-entropy to minimize false negatives in the few-shot target domain, and (3) a regularized alignment term to match class-conditional prediction distributions between source and target domains. By combining these elements, RegAlign performs few-shot domain adaptation more effectively and ensures balanced optimization across source and target datasets. Assume labeled few-shot target data ${{{\mathcal{D}}}}_{T}^{\ell }={\{({x}_{j}^{T},{y}_{j}^{T})\}}_{j=1}^{m}$ with ${y}_{j}^{T}\in \{0,1\}$.

(1) Source focal classification loss

12 $${{{\mathcal{L}}}}_{{{\rm{cls}}}}^{S}=\frac{1}{n}\mathop{\sum }\limits_{i=1}^{n}{\left(1-{p}_{t,i}^{S}\right)}^{{\gamma }_{S}}\,{{\rm{BCE}}}\left({\widehat{p}}_{i}^{S},{y}_{i}^{S}\right),\,{p}_{t,i}^{S}=\left\{\begin{array}{ll}{\widehat{p}}_{i}^{S},& {y}_{i}^{S}=1,\\ 1-{\widehat{p}}_{i}^{S},& {y}_{i}^{S}=0,\end{array}\right.$$

where ${{\rm{BCE}}}(\widehat{p},y)=-[y\log \widehat{p}+(1-y)\log (1-\widehat{p})]$, ${p}_{t,i}^{S}$ is the probability assigned to the true class for source sample *i*, and *γ*_*S*_≥0 is the source focal focusing parameter.

(2) Target class-weighted (and optional focal) classification loss

13 $${{{\mathcal{L}}}}_{{{\rm{cls}}}}^{T}=\frac{1}{m}\mathop{\sum }\limits_{j=1}^{m}{w}_{j}\,{\left(1-{p}_{t,j}^{T}\right)}^{{\gamma }_{T}}\,{{\rm{BCE}}}\left({\widehat{p}}_{j}^{T},{y}_{j}^{T}\right),\,{w}_{j}=\left\{\begin{array}{ll}\alpha,& {y}_{j}^{T}=1,\\ 1,& {y}_{j}^{T}=0,\end{array}\right.$$

with

14 $${p}_{t,j}^{T}=\left\{\begin{array}{ll}{\widehat{p}}_{j}^{T},& {y}_{j}^{T}=1,\\ 1-{\widehat{p}}_{j}^{T},& {y}_{j}^{T}=0.\end{array}\right.$$

Here, *α* > 1 upweights positive target samples to reduce false negatives, and *γ*_*T*_≥0 is the target focal parameter; when *γ*_*T*_ = 0, this reduces to class-weighted BCE.

(3) Class-conditional alignment Using labeled source and labeled target class sets ${S}_{c}=\{i\,| \,{y}_{i}^{S}=c\}$ and ${T}_{c}=\{j\,| \,{y}_{j}^{T}=c\}$,

15 $${\mu }_{S}^{(c)}=\frac{1}{| {S}_{c}| }\mathop{\sum }\limits_{i\in {S}_{c}}{\widehat{p}}_{i}^{S},\,{\mu }_{T}^{(c)}=\frac{1}{| {T}_{c}| }\mathop{\sum }\limits_{j\in {T}_{c}}{\widehat{p}}_{j}^{T},\,{{{\mathcal{L}}}}_{{{\rm{align}}}}^{S\leftrightarrow T}=\mathop{\sum }\limits_{c=0}^{1}{\left({\mu }_{T}^{(c)}-{\mu }_{S}^{(c)}\right)}^{2}.$$

(4) RegAlign final objective is

16 $${{{\mathcal{L}}}}_{{{\rm{RegAlign}}}}={{{\mathcal{L}}}}_{{{\rm{cls}}}}^{S}+\tau \,{{{\mathcal{L}}}}_{{{\rm{cls}}}}^{T}+\lambda \,{{{\mathcal{L}}}}_{{{\rm{align}}}}^{S\leftrightarrow T}.$$

Here, *τ*≥0 balances target supervision against source supervision, and *λ*≥0 controls the strength of source–target class-conditional alignment.

RegAlign differs from the comparator methods in both supervision setting and optimization target. Correlation remover operates at the feature level by reducing linear dependence on sensitive attributes, while kernel mean matching reweights source samples to better match the target marginal distribution. The SSDA baseline instead relies on entropy minimization and pseudo-label-based alignment on unlabeled target data. In contrast, RegAlign is designed for few-shot labeled target adaptation and explicitly combines source-side focal learning, target-side class-weighted supervision, and class-conditional alignment within a single objective. This formulation is intended to improve adaptation to underrepresented populations in clinically imbalanced settings.

### Model interpretation via tree-based SHAP

We interpreted the XGBoost classifier using SHapley Additive exPlanations (SHAP)^54,55^ computed with the TreeExplainer framework (https://shap.readthedocs.io/en/latest/generated/shap.TreeExplainer.html). SHAP values were derived from raw logit for each trained model configuration described in the Experimental Settings section to quantify the contribution of individual brain regions to dementia classification.

We also extended our prior ALE-based meta-analysis framework^37^ using GingerALE (v3.0.2)^56^ in MNI space with a whole-brain mask and cluster-level family-wise error control. The input dataset comprised 1609 foci from 168 experiments and 4152 total subjects. Statistical inference followed cluster-level family-wise control with a cluster-forming threshold of uncorrected *p* < 0.001, cluster-level significance threshold *p* < 0.05, and 100 permutation samples. The resulting thresholded ALE map identified six significant clusters (minimum cluster size  = 9616 mm^3^; supra-threshold volume  = 157,360 mm^3^). Peak convergence was observed bilaterally in medial temporal/limbic regions (including parahippocampal, hippocampal, and amygdalar territories), posterior cingulate-precuneus, and lateral temporo-parietal association cortices, consistent with distributed neurodegeneration patterns across AD/MCI-related cohorts. The thresholded ALE map was then binarized and used as an external anatomical reference for cross-population SHAP comparisons.

### Evaluation metrics

Dementia classification performance was assessed using balanced accuracy (BA) and two fairness measures. BA, the average of sensitivity or true positive rate (TPR) and specificity or true negative rate (TNR), captures variances between normally cognitive and demented classes, offering a more comprehensive evaluation than standard accuracy. This metric proved effective in handling the unbalanced datasets observed in this study.

To assess the fairness of the models across three racial and ethnic groups, we examined disparities in the false positive rate (FPR) and false negative rate (FNR) between the groups, similar to previous studies^18,21^. FPR has important implications in clinical trials, such as differential access to appropriate care or misallocation of medical resources^21^. This misallocation not only exposes healthy individuals to potential side effects but also reallocates essential resources away from patients who genuinely need intervention. Similarly, FNR has critical clinical implications, as it can lead to underdiagnosis, delaying access to timely interventions and appropriate care for individuals who may have dementia. In this study, FPR and FNR discrepancies were calculated by comparing each pair of groups: NHW vs. NHA, NHW vs. HISP, and NHA vs. HISP. The differences from these three comparisons were then averaged to calculate a single metric per group, referred to as the average discrepancy (AvD). Additionally, GridSearchCV is used for hyperparameter tuning with 100-run of Monte Carlo cross-validation to achieve optimal model performance across multiple training and testing splits. This hyper-parameter tuning is described in Supplementary Table 1.

### Reporting summary

Further information on research design is available in the Nature Portfolio Reporting Summary linked to this article.

## Supplementary information

Supplementary Information

Description of Additional Supplementary Files

Supplementary Data 1

Supplementary Data 2

Supplementary Data 3

Supplementary Data 4

Supplementary Data 5

Supplementary Data 6

Reporting Summary

Transparent Peer Review file

## Source data

Source Data

## Data Availability

The minimum dataset used in this study was obtained from four third-party clinical research resources: the National Alzheimer’s Coordinating Center (NACC), the Standardized Centralized Alzheimer’s & Related Dementias Neuroimaging initiative (SCAN), the Alzheimer’s Disease Neuroimaging Initiative (ADNI), and the South Texas Alzheimer’s Disease Research Center (STAC). Because these are human-participant datasets, access is restricted to protect participant privacy and is governed by each resource’s data-use procedures and agreements. NACC clinical and related research data are available to qualified researchers worldwide through the NACC Data Request Process (https://www.naccdata.org/data-request-process). Investigators must submit a data request describing the proposed project and accept the NACC Data Use Agreement. It will acknowledge requests within three business days and may request clarification if needed. Approved datasets are provided through secure download mechanisms maintained by NACC. SCAN harmonized neuroimaging-derived data, including summary, quality-control, and analysis variables, and defaced SCAN images are available through NACC’s data request system (https://scan.naccdata.org/; see also NACC Data Request Process at https://www.naccdata.org/data-request-process). Access is restricted because the resource contains human neuroimaging data and is subject to de-identification, quality-control, and data-use requirements. All submitted image data may be immediately available because additional processing steps, including defacing and QC, may be required. ADNI data are available to qualified investigators through the Laboratory of Neuro Imaging (LONI) Image and Data Archive (IDA) after acceptance of the ADNI Data Use Agreement and submission of an online application describing the investigator’s institutional affiliation and proposed data use (https://adni.loni.usc.edu/data-samples/adni-data/). Applications are generally reviewed within approximately two weeks. Approved users receive login credentials to access and download the requested data through IDA. To access STAC data, investigators and research groups may request patients, control subjects, biospecimens, and data, including through its Population Neuroscience request process (https://stxadrc.org/for-scientists/). Access is therefore restricted and managed directly by STAC. Researchers seeking access should submit a request through the STAC scientist portal. The authors do not have permission to redistribute these third-party datasets. Researchers must obtain access directly from the corresponding data custodians under the conditions described above. Source data are provided with this paper.

## Code availability

The code supporting this study is available at: https://github.com/UTHSCSA-NAL/explainable_and_fair_ML_for_ADRD.
