# Supplementary material for: Advancing fair and explainable machine learning for neuroimaging dementia pattern classification in multi-racial and multi-ethnic populations
Source: Nat Commun. 2026 Jun 26;17:8026. doi: 10.1038/s41467-026-74515-w (PMC13454470; doi:10.1038/s41467-026-74515-w)
Supplement: Supplementary file 1 [file 41467_2026_74515_MOESM10_ESM.pdf]

nature portfolio

Peer Review File

Advancing Fair and Explainable Machine Learning for Neuroimaging Dementia Pattern Classification in Multi-Racial and Multi-Ethnic Populations

Corresponding Author: Dr Mohamad Habes

This file contains all reviewer reports in order by version, followed by all author rebuttals in order by version.

Version 0:

Reviewer comments:

Reviewer #1

(Remarks to the Author)
The manuscript presents a compelling study at the intersection of neuroimaging, machine learning, and health equity. Its core contribution lies in the development of RegAlign, a fairness-aware, few-shot learning approach for improving dementia classification across racially and ethnically diverse populations. The proposed method combines focal weighting, class-sensitive loss, and class-conditional alignment to mitigate diagnostic discrepancies observed in traditional ML pipelines.

The results are noteworthy. RegAlign outperforms several baseline and mitigation strategies in both balanced accuracy and fairness metrics (false positive/negative rate discrepancies). The use of interpretable machine learning via SHAP, in conjunction with ALE meta-analysis, further reinforces the connection between model behavior and known biomarkers of Alzheimer's disease, such as hippocampal atrophy.

From a field impact perspective, the work is relevant to ongoing discussions in medical AI, particularly in bias reduction and equitable healthcare. It addresses well-documented limitations in prior studies that disproportionately reflect outcomes from overrepresented populations. While the integration of known methods into the RegAlign loss is somewhat incremental, the empirical evidence supporting its performance makes it a meaningful contribution to applied neuroinformatics.

That said, several limitations temper the strength of the conclusions. The study is based exclusively on the NACC dataset and includes only three racial/ethnic groups, limiting the scope of generalizability. The absence of external validation—on an independent dataset or in a clinical deployment scenario—precludes broader claims about real-world robustness. Moreover, while SHAP results are informative in majority cohorts, interpretability is notably unstable in underrepresented groups, which undercuts one of the manuscript's primary assertions.

Methodologically, the framework is technically sound but lacks transparency in key areas. The RegAlign loss introduces multiple hyperparameters, yet the tuning process is under-specified and no sensitivity analysis is provided. This raises concerns about reproducibility and overfitting, particularly in the smaller cohorts. Additionally, while the data processing and model design are described at a high level, more detailed implementation steps (e.g., data leakage prevention, SHAP background configuration) would improve reproducibility.

In summary, the manuscript advances an important conversation on fairness in dementia diagnosis through thoughtful integration of machine learning and domain adaptation techniques. However, the scope of generalizability, interpretability consistency in minority cohorts, and methodological transparency must be addressed more fully to support the broad claims made. With revisions that strengthen these aspects, the paper could offer both practical utility and conceptual value across neuroscience, medical imaging, and ethical AI fields.

(Remarks on code availability)
I reviewed the structure and contents of the GitHub repository but did not attempt to install or run the code to reproduce the results. The repository appears to be reasonably organized and includes key components relevant to the manuscript. A README file is provided, offering a basic overview of the project and instructions for usage. While the codebase seems to be a usable starting point for the community, a more detailed step-by-step guide, including environment setup, example data,

and instructions for reproducing the main experiments, would strengthen its utility and reproducibility.

Reviewer #2

(Remarks to the Author)
Summary: This manuscript presents a framework for fair and explainable dementia classification using structural MRI data drawn from White American (NHW), African American (NHA) and Hispanic (HISP) participants in the NACC cohort. Authors propose RegAlign, a composite loss combining focal weighting, class-weighted binary cross-entropy, and a distribution-alignment term. Authors claim RegAlign has the potentiality to account for ethnical diversity in dementia disease. For testing this, RegAlign is compared against five bias-mitigation baselines (Combat-GAM, Correlation Remover, KMM, SSDA, XGBoost) using balanced accuracy, error rates, and cross-ethnic discrepancy. A GPU-accelerated SHAP approximation and ALE-based meta-analysis are used for interpretation. While the approach targets a critical need for equitable AI in neuroimaging, the manuscript would benefit from clearer methodological descriptions and additional experiments to validate RegAlign's advantages.

Major Comments
1. Clarify Domain-Adaptation Setup
The roles of "source" and "target" domains in KMM and SSDA are not fully specified, especially in single-group vs. ALL training. Code inspection suggests KMM was applied coherently only in the ALL setting, while in the other proposing setting it performs a self realignment. As a suggestions, authors are invited, for each experiment, to precisely describe which samples are treated as source/target, how target samples (if any) are used, and provide a summary table of splits and sample counts to ensure reproducibility.

2. Isolate RegAlign Components
RegAlign merges three loss terms, but their individual effects are not quantified. Authors should perform ablations (e.g. focal loss alone, alignment term alone, pairwise combinations) and report performance differences (with significance tests over cross-validation folds). This will reveal whether the composite loss yields synergistic gains or if a single term drives most improvements.

3. Evaluate Realistic Few-Shot Adaptation
Reviewers argue that authors are not really testing domain-adaptation. In the two proposed cases (ALL/SINGLE), the adaptation techniques are either applied with strong leakage of ethnic groups (in the ALL case), or no adaptation is performed. In the case of RegAlign test, technique adopted risk to be effective due to class-imbalance handling techniques rather than proper domain adaptation. A more realistic protocol is to train on two groups and test on an external group, or rather testing on one group and using a little sample size with domain adaptation applied on this small subset for later validation on the remainder. Comparing RegAlign, KMM, and SSDA under the same few-shot budget will better demonstrate generalization benefits.

4. Address SHAP Reference Baseline
Using the feature mean as the SHAP reference can bias attributions, especially for skewed MRI features. Acknowledge this limitation explicitly and, if possible, compare with an alternative (e.g. full TreeSHAP/LIME) on a subset to validate attribution consistency.

Final Recommendation The work addresses an important equity challenge and introduces a promising loss formulation. However, clarifications on domain-adaptation protocols, component ablations, realistic few-shot evaluations, and possibly interpretability limitations are needed to strengthen the manuscript's validity and reproducibility. Upon revision, this study would make a valuable contribution to fair AI in healthcare but significant changes as described above are advised.

(Remarks on code availability)
See major comments.

Reviewer #3

(Remarks to the Author)
Overall, the manuscript is good. The authors present an explainable machine learning for neuroimaging dementia pattern classification in multiethnic population. In this manuscript, authors present a framework that combines XGBoost classifiers with domain alignment strategies, culminating in the introduction of a custom loss function RegAlign for few-shot domain adaptation. Using multi-ethnic MRI datasets from the NACC, the study evaluates the performance of various discrepancy mitigation methods, and applies interpretable machine learning tools such as SHAP, ALE to model decisions in biological plausibility. In general, I find the manuscript relevant. However, the authors have to address the following points to enhance the clarity and overall quality of the manuscript.
The points for revision are as follows:
1. In the introduction part: briefly elaborate on how epigenetic variability introduces unique methodological and interpretive challenges for machine learning prediction.
2. SHAP dependence plots from underrepresented groups show erratic trends. This might reflect overfitting rather than population-specific biomarkers, please check this.
3. Statistical significance testing is missing for discrepancy improvements across groups.
4. The efficacy of RegAlign under truly limited sample conditions is unclear, as the NHA and HISP cohorts remain relatively

small (n=337 and n=249, respectively) and Some methods may not have been tuned with equal care as RegAlign, potentially biasing comparisons.
5. Please make sure consistency in terminology and Briefly described the limitations and future work.

(Remarks on code availability)
Yes, I have reviewed the code associated with the paper. Overall, the code appears to be a usable resource for the research community.

Reviewer #4

(Remarks to the Author)
I co-reviewed this manuscript with one of the reviewers who provided the listed reports. This is part of the Nature Communications initiative to facilitate training in peer review and to provide appropriate recognition for Early Career Researchers who co-review manuscripts.

(Remarks on code availability)

Version 1:

Reviewer comments:

Reviewer #1

(Remarks to the Author)
This manuscript presents a machine-learning framework for fair and explainable dementia classification using neuroimaging data across multi-ethnic populations. The study systematically investigates performance disparities in dementia prediction models and introduces RegAlign, a discrepancy-mitigation framework that integrates focal loss, class-weighted target supervision, and class-conditional alignment to improve cross-population generalization. The results demonstrate that standard models exhibit measurable performance gaps across ethnic groups even after demographic balancing and harmonization, while the proposed RegAlign method significantly improves balanced accuracy and reduces false-negative disparities in underrepresented populations.
The work is timely and relevant to the broader fields of medical AI, neuroimaging analytics, and fairness in machine learning. The authors provide strong empirical evidence across multiple cohorts and validate their approach with robust cross-validation procedures and external validation datasets. Importantly, the interpretability analysis using TreeSHAP highlights biologically meaningful brain regions associated with dementia while also revealing population-specific patterns of model attribution. These findings contribute to the ongoing discussion about algorithmic fairness in clinical decision support systems and underscore the importance of heterogeneous training data in medical AI development.
Overall, the methodology appears sound and the conclusions are well supported by the results. The revisions have strengthened the manuscript by expanding the dataset, clarifying the domain-adaptation framework, improving hyperparameter tuning transparency, and addressing reproducibility considerations. The study meets expected standards for methodological rigor and provides sufficient detail for reproducibility. In my view, the work makes a valuable contribution to the literature on equitable AI in healthcare and is suitable for publication.

(Remarks on code availability)
The authors provide a publicly accessible code repository containing the main components required to reproduce the experiments described in the manuscript. The repository appears reasonably well organized and includes the implementation of the proposed RegAlign method as well as the baseline models used in the study. A README file is provided with instructions describing the overall workflow and guidance for running the training and evaluation procedures. The authors have further improved the repository following reviewer feedback by adding a more detailed installation guide, example commands for running the experiments, and a small demonstration dataset that allows users to test the pipeline. These additions enhance the usability of the code and support reproducibility of the main analyses. While future versions of the repository could include additional scripts for reproducing all manuscript figures automatically, the current codebase represents a useful and practical resource for the research community.

Reviewer #2

(Remarks to the Author)
Dear Authors,

Thank you for the thorough revision of the manuscript and the detailed responses to the reviewers’ comments. I appreciate the effort you invested in addressing the concerns raised during the previous review round.

In particular, the addition of external validation datasets and the expanded analyses substantially strengthen the manuscript. The inclusion of multiple cohorts and the evaluation across in-distribution and out-of-distribution datasets improve the credibility of the conclusions and address the earlier concerns regarding generalizability. The clarification of methodological details and the expanded discussion also improve the overall readability and transparency of the work.
Overall, I find that my previous comments have been adequately addressed and the manuscript has improved considerably.

My only remaining minor suggestion concerns the figures. Some of them are relatively small and difficult to read in their current form. I would encourage the authors to improve figure readability in the final version (e.g., by increasing font sizes, simplifying panels, or enlarging key elements).

Other than this minor point, I have no further comments or suggestions.

Best regards.

(Remarks on code availability)

Reviewer #3

(Remarks to the Author)
Thank you, authors, for addressing previous comments. The manuscript is strong overall, but a few points still need clarification. There are clear typos and AI generated artifacts e.g., “Perfect point…” and typos “TThe” (page144 and 141) that interrupt the academic tone and should be removed. Some sections remain overly verbose and inconsistent. Finally, the novelty of RegAlign is still not clearly differentiated from existing domain adaptation methods. Addressing these points will improve clarity and strengthen the manuscript’s contribution.

(Remarks on code availability)

Reviewer #4

(Remarks to the Author)
I co-reviewed this manuscript with one of the reviewers who provided the listed reports. This is part of the Nature Communications initiative to facilitate training in peer review and to provide appropriate recognition for Early Career Researchers who co-review manuscripts.

(Remarks on code availability)

Open Access This Peer Review File is licensed under a Creative Commons Attribution 4.0 International License, which permits use, sharing, adaptation, distribution and reproduction in any medium or format, as long as you give appropriate credit to the original author(s) and the source, provide a link to the Creative Commons license, and indicate if changes were made.
In cases where reviewers are anonymous, credit should be given to 'Anonymous Referee' and the source.
The images or other third party material in this Peer Review File are included in the article’s Creative Commons license, unless indicated otherwise in a credit line to the material. If material is not included in the article’s Creative Commons

license and your intended use is not permitted by statutory regulation or exceeds the permitted use, you will need to obtain permission directly from the copyright holder.
To view a copy of this license, visit https://creativecommons.org/licenses/by/4.0/

We are grateful for the insightful and constructive feedback from the reviewers and editors, which has substantially improved the quality and clarity of our work. We have incorporated all reviewers' suggestions. Changes are highlighted in red in the revised manuscript. Please see below, in blue, for a point-by-point response to the reviewers' comments. All page numbers refer to the revised manuscript file with tracked changes.

Reviewer #1

Reviewer#1 Point#1: The study is based exclusively on the NACC dataset and includes only three racial/ethnic groups, limiting the scope of generalizability. The absence of external validation—on an independent dataset or in a clinical deployment scenario—precludes broader claims about real-world robustness.

- **Response to Point#1**: Thank you for highlighting the important issue of generalizability.

  Our work was originally conceived as a fairness-focused, multi-ethnic dementia prediction study, and NACC cohort was chosen because it provides relatively balanced control and dementia samples across major U.S. racial/ethnic groups such as Non-Hispanic White (NHW), Non-Hispanic African (NHA), and Hispanic (HISP). In the revision, we collected large multiple Alzheimer's disease research cohorts with imaging and race/ethnicity information that are accessible to us, including extended NACC, SCAN, ADNI, STAC, HABS-HD, and UK Biobank (UKBB).

  The National Alzheimer's Coordinating Center (NACC; https://www.alz.washington.edu/) aggregates standardized clinical and neuroimaging data from Alzheimer's Disease Centers (ADCs) across the U.S. The Standardized Centralized Alzheimer's & Related Dementias Neuroimaging (SCAN; https://scan.naccdata.org/) cohort is a National Institute on Aging–funded collaboration across the U.S. Alzheimer's Disease Research Centers (ADRC) program. The Alzheimer's Disease Neuroimaging Initiative (ADNI; https://adni.loni.usc.edu/) is a large, longitudinal, multi-center observational study conducted at more than 60 sites in the United States and Canada. The South Texas Alzheimer's Research Center (STAC) operates as a clinical research cohort designed to reflect real-world recruitment and care pathways in a predominantly Hispanic region. We also evaluated UKBB and HABS-HD. However, UKBB presents complex race/ethnicity definitions, and very limited AD case counts relative to sample size. HABS-HD similarly has very few dementia cases. Therefore, we did not include UKBB and HABS-HD in the present analysis.

  Finally, we have now extended the study to a multi-cohort setting by including SCAN cohort as part of the training and internal validation, and we incorporated the independent ADNI and STAC cohorts for external validation. To ensure that individuals from smaller racial/ethnic categories were not omitted, individuals identifying as Asian, Native Hawaiian, Alaska Native, American Indian, or unknown ethnicity were combined into an "Other" group. This new group was exclusively used for model validation.

As indicated in the revised **Figure 1 panel A**, our experiments now involve three data sets:

- A **group-balanced set** derived from NACC and SCAN, where class labels and ethnic composition (NHW, NHA, HISP) are strictly matched for age and sex to minimize confounding.
- An **external in-distribution set**, containing all the NACC and SCAN individuals not included in the group-balanced set, which includes all the individuals in the NACC and SCAN cohorts in the "Other" group.
- An **external out-of-distribution set** containing all ADNI and STAC individuals, where we re-examine model behavior across previously seen ethnic groups (NHW, NHA, HISP) and the unseen "Other" group.

Our expanded experiments demonstrate that the fairness improvements introduced by our proposed method are not restricted to the group-balanced set. As detailed in section of **Supplementary Performance on External Datasets** (see **pages S10–S11**), our method not only maintains strong performance on external cohorts but also reduces performance gaps across ethnic groups, and fairness gains are observed for both the originally modeled groups (NHW, NHA, HISP) and the unseen "Other" group. Particularly, on the out-of-distribution ADNI+STAC cohort, RegAlign consistently improves balanced accuracy across all ethnic groups, most notably in NHA and HISP groups, while simultaneously reducing both false negative and false positive rates, indicating more reliable dementia detection under different recruitment and imaging protocols. Furthermore, the cross-ethnic performance gaps reduced on ADNI+STAC, showing that improvements are not driven solely by the majority cohort. On the external in-distribution NACC+SCAN set, RegAlign again improves performance for NHW and further increases performance for the unseen "Other" group, demonstrating that the method's fairness gains generalize beyond the training distribution and extend to smaller or unmodelled racial/ethnic categories without sacrificing overall accuracy.

- **Action Taken to Point #1**:
  - Expanded the study to include SCAN as an additional cohort for training and validation.
  - Added ADNI and STAC as external validation cohorts, held out entirely from model training. Updated Supplementary Information, section of **Performance on External Datasets** (see **page S10-S11**) and **Supplementary Fig. 6-7** (see **page S17-S18**) to derive performance on external sets as well as "Other" ethnicity group.
  - Revised the **Method - Study Populations section (**see **pages 3-4)** to describe cohort sources, roles, and inclusion criteria.
  - Updated **Table 1 (**see **page 20)** to reflect the expanded dataset.
  - The final dataset now includes 9,007 participants:
    - White: 6,584 (4,347 CN / 2,237 AD)
    - Black: 1,263 (1,070 CN / 193 AD)
    - Hispanic: 713 (496 CN / 217 AD)
    - Other: 447 (310 CN / 137 AD)

<u>Reviewer#1 Point#2</u>: Moreover, while SHAP results are informative in majority cohorts, interpretability is notably unstable in underrepresented groups, which undercuts one of the manuscript's primary assertions.

- **Response to Point#2**: Thank you for this comment.

We would like to clarify that our primary goal is not to claim uniform interpretability across populations, but rather to systematically characterize how model behavior differs across ethnic groups under carefully controlled conditions. In the revision, we now explicitly frame the interpretability analysis as part of a broader, structured assessment of performance discrepancies across NHW, NHA, and HISP populations, using group-balanced evaluation, demographic matching, and feature harmonization to reduce confounding by sample size, age, gender, or site.

On this controlled dataset, we recomputed SHAP values using TreeSHAP, which yields exact, model-consistent attributions for the XGBoost classifier. The resulting SHAP-based brain maps show that core AD-related regions are consistently involved across all three ethnic groups, indicating that models capture a common neuroanatomical substrate of dementia when confounding factors are minimized. At the same time, both the brain maps and the partial dependence plots reveal systematic, population-specific differences in the magnitude of regional effects at the individual ROI level. We view these differences as informative rather than as instability. These differences could suggest that:

- Racial/ethnic groups may differ in the relative burden of Alzheimer's, which can shift the spatial pattern of atrophy,
- Clinical pathways and timing of diagnosis can vary by ethnicity,
- Life-course exposures and social determinants of health correlate with ethnicity.

Overall, the SHAP-based brain maps on the group-balanced set highlight a common set of AD-related regions across all groups, while the partial dependence visualizations highlight ethnicity-specific modulation of these effects. These findings directly support our central hypothesis that population-specific factors, linked to ethnicity and associated life-course exposures, shape both how dementia manifests in the brain and how predictive models learn to use regional information.

- **Action Taken to Popint#2**:
  - We added text in the Results to clarify that we systematically investigate performance discrepancies across ethnic groups using a strictly group-balanced subset stratified by diagnosis and ethnicity from NACC and SCAN (426 CN / 426 DA; 142 CN and 142 DA per ethnicity), with age and gender matching across groups (**Table 2,** see **page 21**).
  - Recomputed SHAP values using **TreeSHAP** (section of **Model Interpretation via Tree-based SHAP,** see **page 7**), which provides exact and model-consistent attributions for XGBoost classifier.
  - Updated **Figure 3** (see **page 24**) to reflect revised SHAP analyses on the balanced dataset.
  - Updated **Supplementary Fig. 11 (**see **pages S22-S26)** to highlight population-specific attribution patterns across all MUSE-derived brain regions.

Reviewer#1 Point#3: Methodologically, the framework is technically sound but lacks transparency in key areas. The RegAlign loss introduces multiple hyperparameters, yet the tuning process is under-specified and no sensitivity analysis is provided. This raises concerns about reproducibility and overfitting, particularly in the smaller cohorts.

- **Response to Point#3**: Thank you for pointing out the need for hyperparameter tuning and sensitivity analysis. During the revision, a systematic hyperparameter tuning was conducted for all RegAlign components using a 100-run Monte Carlo cross-validation. All RegAlign-specific parameters were tuned during the inner cross-validation loop, ensuring that parameter selection was strictly separated from test and external evaluation sets. Moreover, we expanded the sample size for both African American (1,263 subjects) and Hispanic (713 subjects) populations, thus increasing statistical power and improving the stability of the optimization process. A dedicated hyperparameter sensitivity analysis was conducted following the Top-K robustness framework of Adkins et al. (2024). For each method, including RegAlign, we evaluated performance across a wide grid of hyperparameter settings and quantified how often near-optimal performance is achieved across this space. And lastly, we evaluated the best-performing configurations on fully external datasets and across ethnicity groups, including the unseen "Other" group, to directly connect hyperparameter robustness to generalization and fairness. As detailed in the **Supplementary Performance on External Datasets** section (see **pages S10–S11**), RegAlign maintains or improves overall performance on ADNI and STAC while reducing performance gaps among NHW, NHA, HISP, and the unseen "Other" ethnicity group.

- **Action Taken to Point#3**:
    - Re-implemented all experiments for all methods using 100-run Monte-Carlo cross-validation, where RegAlign and other comparative methods' hyperparameters were tuned within the inner 10-fold cross-validation loop for each run.
    - Applied the best-performing parameter configuration to the held-out test folds and external validation cohorts only after tuning was complete.
    - Updated **Supplementary Table 1** (see **pages S1-S2**) to report the full hyperparameter search ranges for all methods.
    - Performed a sensitivity analysis based on Top-K robustness approach (**Adkins et al., 2024**) to evaluate performance across hyperparameter ranges for all methods and added the results as **Supplementary Table 4** and **Supplementary Figure 9 (**see **pages S11, S20 and S21)**.
      "Adkins, J., Bowling, M. and White, A., (2024). A method for evaluating hyperparameter sensitivity in reinforcement learning. Advances in Neural Information Processing Systems, 37, pp.124820-124842."

<u>Reviewer#1 Point#4</u>: Additionally, while the data processing and model design are described at a high level, more detailed implementation steps (e.g., data leakage prevention, SHAP background configuration) would improve reproducibility.

- **Response to Point#4**: Thank you for this comment. To prevent data leakage, we included only the latest available visit per subject, ensuring that everyone appears in the dataset exactly once and that no subject contributes data to both training and test sets. The **Experimental Settings** section in the Supplementary Information (see **pages S1–S4**) was also updated to provide detailed descriptions of hyperparameter tuning for classifier models and discrepancy mitigation methods (**Supplementary Table 1**), the training and evaluation procedure (**Supplementary Fig. 1**), and data acquisition strategies for domain adaptation. Lastly, the custom GPU-accelerated SHAP approximation was replaced with a TreeSHAP implemented via the public

shap.TreeExplainer toolbox (https://shap.readthedocs.io/en/latest/generated/shap.TreeExplainer.html), using default configurations.

- **Action Taken to Point#4**:
  - Avoid data leakage by selecting one latest visit per subject.
  - Updated the Supplementary Experimental Settings **(pages S1–S4)** with detailed descriptions of hyperparameter tuning (**Supplementary Table 1**), training procedure (**Supplementary Fig. 1**), and domain-adaptation data acquisition.
  - Replaced custom SHAP computation with **TreeSHAP (shap.TreeExplainer)** using default settings (Main Manuscript, section of **Model Interpretation via Tree-based SHAP,** see **page 7**).
  - Updated SHAP-based brain heatmaps in **Figure 3** (see **page 24**)

Reviewer#1 Point#5 on code availability: I reviewed the structure and contents of the GitHub repository but did not attempt to install or run the code to reproduce the results. The repository appears to be reasonably organized and includes key components relevant to the manuscript. A README file is provided, offering a basic overview of the project and instructions for usage. While the codebase seems to be a usable starting point for the community, a more detailed step-by-step guide, including environment setup, example data, and instructions for reproducing the main experiments, would strengthen its utility and reproducibility.

- **Response to Point#5**: We thank the reviewer for carefully reviewing our GitHub repository. We updated the source codes at: https://github.com/UTHSCSA-NAL/explainable_and_fair_ML_for_ADRD

- **Action Taken to Point#5**:
  - Updated the README to include a step-by-step installation guide, covering environment setup and required dependencies.
  - Added explicit instructions and example commands for reproducing the main training, evaluation, and fairness analyses.
  - Included a small example dataset (non-identifiable, derived features) to demonstrate the workflow.
  - Clarified the expected directory structure and configuration files to facilitate end-to-end reproducibility.

Reviewer #2

Reviewer#2 Point#1: Clarify Domain-Adaptation Setup

The roles of "source" and "target" domains in KMM and SSDA are not fully specified, especially in single-group vs. ALL training. Code inspection suggests KMM was applied coherently only in the ALL setting, while in the other proposing setting it performs a self realignment. As a suggestions, authors are invited, for each experiment, to precisely describe which samples are treated as source/target, how target samples (if any) are used, and provide a summary table of splits and sample counts to ensure reproducibility.

- **Response to Point#1**: Thank you for the comment. To address this concern, we now explicitly define domain-adaptation splits for each method and scenario (section of **Supplementary Data Acquisition for Domain Adaptation**, see **pages S2-S3**). In our framework, instance-based (KMM) and objective-based (SSDA/RegAlign) approaches differ in how source and target domains are constructed, while downstream training and evaluation protocols remain consistent across methods. Details are provided as follows:
    - **KMM** was implemented as an instance-based reweighting strategy. For each ordered source–target ethnicity pair, we fit a KMM model using training samples from the source group as the source domain and samples from the target group as the target domain, yielding nine pre-computed weight mappings, $\omega_{source \rightarrow target}$: $\omega_{NHW \rightarrow NHA}$, $\omega_{NHW \rightarrow HISP}$, $\omega_{NHA \rightarrow NHW}$, $\omega_{NHA \rightarrow HISP}$, $\omega_{HISP \rightarrow NHW}$, $\omega_{HISP \rightarrow NHA}$, $\omega_{NHW \rightarrow Other}$, $\omega_{NHA \rightarrow Other}$, $\omega_{HISP \rightarrow Other}$

      These weights were computed prior to model training and held fixed during classifier fitting. They were then mapped to each training scenario as in section of **Supplementary Data Acquisition for Domain Adaptation** (see **page S2-S3**)

    - **SSDA** and **RegAlign** were implemented as objective-based alignment approaches with source and target domains jointly optimized during training.
        - *ALL training*: the source domain consisted of the training split from all ethnic groups, while the target domain was defined as the held-out test split. This formulation aligns the learned representation with the evaluation distribution without leaking supervised information from the test set.
        - *Two-group training*: the source domain comprised the included groups, and the target domain was the held-out group.
        - *Single-group training*: the source domain was the training set of the selected group, and the target domain pooled the remaining groups.

- **Action Taken to Point#1**:
    - Added a dedicated section "**Supplementary Data Acquisition for Domain Adaptation**" (see **pages S2-S3**) clarifying source and target domain definitions for KMM, SSDA, and RegAlign.
    - Added **Supplementary Table 2** (see **page S6**) for detailing source/target definitions and sample counts for each experiment.

Reviewer#2 Point#2: Isolate RegAlign Components

RegAlign merges three loss terms, but their individual effects are not quantified. Authors should perform ablations (e.g. focal loss alone, alignment term alone, pairwise combinations) and report performance differences (with significance tests over cross-validation folds). This will reveal whether the composite loss yields synergistic gains or if a single term drives most improvements.

- **Response to Point#2**: Thank you for your insightful suggestion. We conducted a comprehensive ablation study that decomposes RegAlign into its constituent loss components for evaluation. RegAlign consists of three objective terms: a **source-domain classification loss**, **a target-domain classification loss**, and a **class-conditional alignment loss**. The source-domain classification loss serves as the primary objective and provides a baseline when used alone. We evaluated three configurations: source-domain classification loss combined with target-domain classification loss, source-domain classification loss combined with class-conditional alignment loss, and the full RegAlign objective that integrates all three losses. The results are presented in dedicated sections of the revised supplementary materials.

- **Action Taken to Point#2**:
  - Added a new "**Supplementary Ablation of RegAlign Loss Components**" section in the Supplementary Information (see **page S10**), detailing the evaluation of individual and pairwise loss terms.
  - Added **Supplementary Figure S5** (see **page S16)** summarizing performance and fairness outcomes across ablation settings.
  - Added a "**Supplementary Statistical Evidence of Cross-Ethnic Discrepancies**" section in the Supplementary Information (see **page S7**).
  - Included Supplementary Tables 7 and 8, reporting statistical significance tests for performance and discrepancy metrics relative to the baseline method.

Reviewer#2 Point#3: Evaluate Realistic Few-Shot Adaptation

Reviewers argue that authors are not really testing domain-adaptation. In the two proposed cases (ALL/SINGLE), the adaptation techniques are either applied with strong leakage of ethnic groups (in the ALL case), or no adaptation is performed. In the case of RegAlign test, technique adopted risk to be effective due to class-imbalance handling techniques rather than proper domain adaptation. A more realistic protocol is to train on two groups and test on an external group, or rather testing on one group and using a little sample size with domain adaptation applied on this small subset for later validation on the remainder. Comparing RegAlign, KMM, and SSDA under the same few-shot budget will better demonstrate generalization benefits.

- **Response to Point#3**: Thank you for the comment. We would like to clarify that the three domain-adaptation methods considered in this study differ fundamentally in their adaptation mechanisms and intended use cases. **KMM** is an instance-based reweighting approach. In our implementation, KMM weights are pre-fitted using all cohorts to model pairwise distribution shifts between ethnic groups and are then held fixed during

downstream classifier training. As such, KMM does not operate in a few-shot setting but instead addresses distribution mismatch through global sample reweighting. **SSDA** is an unsupervised adaptation approach that incorporates target-domain cohort via pseudo-labeling based on K-means clustering. This strategy uses all available target-domain samples without relying on labeled few-shot data. In contrast, **RegAlign** is the only method in our framework designed to support few-shot domain adaptation, which incorporates a small labeled or weakly supervised target stream during training. Importantly, while the adaptation mechanisms differ across methods, the downstream training and evaluation protocols are kept consistent, ensuring fair comparison.

We emphasize that the primary goal of this study is to investigate how model behavior and performance disparities differ across ethnic groups, particularly in settings where models are trained on one population and evaluated on others, as well as in heterogeneous-population training (ALL setting). Nevertheless, to address the reviewer's suggestion, our experiments were expanded to include settings in which training is carried out using two ethnic groups, and testing is conducted exclusively on a third, held-out ethnic group.

- **Action Taken to Point#3**:
  - Clarified the distinct roles of KMM (instance-based), SSDA (unsupervised pseudo-labeling), and RegAlign (few-shot adaptation) in the **Supplementary Experimental Settings** (see **pages S2–S3**).
  - Added Supplementary Data 3 and 4 results for training on two ethnic groups and testing on a held-out group, reported in Supplementary Table 9 and 10..

Reviewer#2 Point#4: Address SHAP Reference Baseline

Using the feature mean as the SHAP reference can bias attributions, especially for skewed MRI features. Acknowledge this limitation explicitly and, if possible, compare with an alternative (e.g. full TreeSHAP/LIME) on a subset to validate attribution consistency.

- **Response to Point#4**: Thank you for this comment.

  We have recomputed all SHAP values using TreeSHAP, which provides exact, model-consistent attributions for XGBoost models (https://shap.readthedocs.io/en/latest/generated/shap.TreeExplainer.html).

- **Action Taken to Point#4**:
  - Recomputed SHAP values using TreeSHAP via shap.TreeExplainer for all XGBoost models.
  - Updated the **Model Interpretation via Tree-based SHAP** section to describe the revised attribution method (see **page 7**).
  - Updated SHAP-based brain heatmap visualization in **Figure 3** (see **page 24**)

Reviewer #3

Reviewer#3 Point#1: In the introduction part: briefly elaborate on how epigenetic variability introduces unique methodological and interpretive challenges for machine learning prediction.

- **Response to Point#1**:

  We thank the reviewer for this thoughtful suggestion. We agree that epigenetic variability plays an important role in dementia risk and progression, and we understand that your comment builds on our statement: "For instance, while the presence of the APOE-ε4 allele is a recognized risk factor among individuals of White descent [15, 26, 27], studies have found that African American and Hispanic populations exhibit elevated Alzheimer's rates regardless of APOE genotype [28, 29]… As a result, model explanations may reflect group-specific biological signatures rather than universal disease markers, which can reduce interpretability and complicate generalization" (**line 23-35**). Therefore, we have expanded the **Introduction** to briefly discuss these methodological and interpretive challenges for machine learning prediction.

- **Action Taken to Point#1**:
  - After "…studies have found that African American and Hispanic populations exhibit elevated Alzheimer's rates regardless of APOE genotype [28, 29]." (**line 24-25**), we added a short paragraph in the third paragraph of the Introduction explaining epigenetic variability as follows (**line 25-35**): "In addition to genetic variation, epigenetic variability, encompassing DNA methylation, histone changes, and many regulatory mechanisms, introduces a significant layer of complexity in the risk and progression of Alzheimer's disease. Epigenetic patterns are influenced by ancestry, social and environmental exposures, and lifestyle factors, potentially affecting both brain morphology and dementia pathology in varying ways across populations [17, 26-28]. For machine learning models, this variability creates unique methodological and interpretive challenges: population-specific epigenetic effects can alter the meaning of imaging-derived features, introduce hidden confounding, and yield attribution patterns that are not transferable across groups [18, 19]. As a result, model explanations may reflect group-specific biological signatures rather than universal disease markers, which can reduce interpretability and complicate generalization."

Reviewer#3 Point#2: SHAP dependence plots from underrepresented groups show erratic trends. This might reflect overfitting rather than population-specific biomarkers, please check this.

- **Response to Point#2**: To address this concern, we have updated the analysis using a strictly group-balanced, harmonized cohort, in which cognitively normal (CN) and dementia (DA) sample counts are matched across all ethnic groups (**Table 2**, see **page 21**). This design reduces variance driven by sample-size imbalance and mitigates overfitting artifacts. The revised results show that, while variability is reduced, distinct response patterns across ethnic groups persist, indicating that ethnicity-associated differences in model performance

and attribution patterns reflect population-specific biological factors rather than instability or overfitting in the interpretability analysis.

- **Action Taken to Point#2**:
  - Constructed a group-balanced cohort with equal CN/DA counts across ethnic groups (**Table 2**, see **page 21**).
  - Updated SHAP dependence plots in **Figure 3** (see **page 24**) and **Supplementary Fig. 11** (see **pages S22–S25**).
  - Revised the Results section to clarify that the observed SHAP patterns persist under harmonization and strict balancing (section of **Supplementary Discrepancy Interpretation via TreeSHAP**, see **pages 10-11**).

<u>Reviewer#3 Point#3:</u> Statistical significance testing is missing for discrepancy improvements across groups.

- **Response to Point#3**: Thank you for pointing this out. To address this concern, we have augmented our analysis with formal statistical testing to assess whether observed performance and disparity reductions are statistically meaningful across cross-validation folds and external cohorts.

- **Action Taken to Point#3**:
  - Added statistical significance testing results in the Supplementary Materials, reported in the following files:
    - *Supplementary Data 1 (supplementary_performance_ttests.csv*): statistical tests for performance metrics.
    - *Supplementary Data 2 (supplementary_gap_ttests.csv)*: statistical tests for cross-ethnic fairness metrics.
    - *Supplementary Data 3 (supplementary_external_adnistac_results.csv)* and Supplementary Data 4 (*supplementary_external_naccscan_results.csv)*: significance testing results for external validation cohorts.
  - Updated the Results sections "**Presence of Discrepancy across Multiple Ethnicity Population**" (see **page 9**) and "**Performance on Discrepancy Mitigation Techniques**" (see **pages 11-12**) to incorporate and reference these statistical analyses.

<u>Reviewer#3 Point#4:</u> The efficacy of RegAlign under truly limited sample conditions is unclear, as the NHA and HISP cohorts remain relatively small (n=337 and n=249, respectively) and Some methods may not have been tuned with equal care as RegAlign, potentially biasing comparisons.

- **Response to Point#4**: To address the limited sample size in underrepresented groups, we expanded the study to include a larger, more diverse cohort of 9,007 unique subjects. In the revised dataset, the number of NHA participants increased to 1,263, and the number of HISP participants increased to 713. In addition, we evaluated RegAlign on a strictly group-balanced dataset, where class counts were matched across ethnic groups (**Table 2**, see **page 21,** and **Figures 2-4**, see **pages 23-25**), as well as on independent external cohorts

to assess generalization (**Supplementary Fig. 6–8**, see **pages S17–S19**). These complementary evaluations demonstrate that RegAlign's effectiveness is not limited to a single data configuration and persists across balanced and external settings.

Furthermore, we included hyper-parameter tuning based on GridSearch for all methods, as described in **Supplementary Table 1** (see **pages S1-S2**), which provides default settings and the search ranges used for optimization.

- **Action Taken to Point#4**:
  - Expanded the study to a large multi-cohort dataset (n = 9,007), substantially increasing representation of NHA and HISP populations.
  - Evaluated RegAlign on both group-balanced cohorts (**Figures 2–4,** see **pages 23-25**) and external validation cohorts (**Supplementary Fig. 6–8,** see **pages S17-S19**).
  - Updated the Results section to reflect these expanded analyses and ensure fair comparison across methods.
  - Updated **Supplementary Table 1** (see **pages S1-S2**) details the default settings and the search ranges used for optimization in GridSearch approach for all classifiers and methods.

<u>Reviewer#3 Point#5</u>: Please make sure consistency in terminology and Briefly described the limitations and future work.

- **Response to Point#5**: Thank you for the comment. We have carefully reviewed the manuscript to ensure consistent terminology is used throughout, particularly for population group definitions, model names, and performance and discrepancy metrics. In addition, we discuss the limitations and future directions of this study in the Discussion section.

- **Action Taken to Point#5**:
  - Standardized terminology across the manuscript for population groups, model components, and evaluation metrics.
  - Added and clarified a Limitations and Future Work subsection in the Discussion section (see **page 14**).

We are grateful for the thoughtful, constructive, and careful feedback from the reviewers and editors. We sincerely appreciate the time and effort they devoted to evaluating our manuscript. We carefully considered all comments, including those raised as minor points, and revised the manuscript accordingly to improve its clarity, presentation, and overall quality. In this final revision, we improved figure readability, corrected typographical and editorial issues, streamlined overly verbose sections, and clarified the novelty of RegAlign relative to existing domain adaptation methods. Below, we provide a point-by-point response describing how each comment was addressed in the revised manuscript.

Reviewer #1

No questions or concerns

Reviewer #2

Reviewer#2 Point#1: My only remaining minor suggestion concerns the figures. Some of them are relatively small and difficult to read in their current form. I would encourage the authors to improve figure readability in the final version (e.g., by increasing font sizes, simplifying panels, or enlarging key elements).

Response to Point#1:

We thank the reviewer for this helpful suggestion. We carefully revised the figures to improve their legibility and overall presentation quality in the final version.

Action Taken to Point #1:

We increased font sizes and enlarged key elements in Figures 1, 2, 3, and 4 to improve readability.

Reviewer #3

Reviewer#3 Point#1: There are clear typos and AI generated artifacts e.g., “Perfect point…” and typos “TThe” (page144 and 141) that interrupt the academic tone and should be removed. Some sections remain overly verbose and inconsistent.

Response to Point#1:

We thank the reviewer for carefully identifying these issues. We conducted a thorough proofreading and editing pass across the entire paper to remove such issues and improve clarity, consistency, and concision throughout.

Action Taken to Point#1:

- We removed the artifact beginning with “Perfect point...”
- We corrected the following typographical errors:
  - “TThe” to “The”
  - “calculated bu comparing” to “calculated by comparing”
  - “washingfaton” to “washington” in the NACC URL
- We revised broader or less precise wording to improve scientific clarity and consistency:
  - Replaced “diverse cultural groups” with “racial and ethnic groups”

    - Replaced "diagnosis discrepancy" with "inter-group performance discrepancies"
    - Replaced "ethnic group discrepancies" with "inter-group performance discrepancies"
    - Replaced "Totally" with "In total"
    - Replaced "most optimal" with "optimal"
- We revised verbose or repetitive passages for concision and consistency:
    - Replaced the paragraph beginning with "Nevertheless, AI-driven diagnostic systems..." and ending with "...offering insight into how risk varies across diverse backgrounds."
    - Shortened the paragraph beginning with "Emerging research has highlighted significant variations..." to reduce over-explanation
    - Removed the repeated sentence "AI-based diagnosis tools can sometimes amplify these discrepancies."
    - Replaced the sentence "These interventions aimed to maintain diagnostic accuracy while enhancing consistent and balanced diagnostic outcomes across groups." with "These approaches were evaluated for their ability to improve diagnostic accuracy while reducing inter-group performance disparities."
- We revised specific sections to improve readability:
    - Shortened the Image Processing subsection
    - Revised the sentence beginning "XGB was selected not only..." to:
      "XGBoost was selected because it performs well on structured, potentially imbalanced data, captures non-linear interactions among brain regions, and supports feature-level interpretation."
    - Revised the sentence beginning "The estimated sample weights are subsequently normalized..." to:
      "The estimated sample weights were normalized to sum to 1 and then incorporated into classifier training. In this way, higher-weighted source samples contributed more strongly to the optimization objective, whereas lower-weighted samples had less influence."
    - Shortened the ALE paragraph in the subsection Model Interpretation via Tree-based SHAP
- We also revised section headings and opening text in the Results section for greater clarity:
    - Replaced the subheading "Presence of Discrepancy across Multiple Ethnicity Population" with "Performance Discrepancies across Racial and Ethnic Groups"
    - Replaced "Potential Discrepancy" with "Baseline Evidence of Inter-group Performance Discrepancy"
    - Shortened the opening paragraph in the Results section to:
      "We first evaluated whether performance discrepancies persisted after controlling for major confounders. To this end, we constructed a strictly group-balanced subset from the NACC and SCAN cohorts, matched by diagnosis, age, and sex across NHW, NHA, and HISP participants. This design allowed us to isolate ethnicity-associated differences in model behavior while minimizing the effects of sample imbalance, demographic mismatch, and site-related variability."

Reviewer#3 Point#2: Finally, the novelty of RegAlign is still not clearly differentiated from existing domain adaptation methods. Addressing these points will improve clarity and strengthen the manuscript's contribution.

Response to Point#2:

We thank the reviewer for this important comment. In response, we revised the Introduction, the Proposed RegAlign subsection, and the Discussion to more explicitly distinguish RegAlign from the other methods and to better define its specific methodological contribution.

Action Taken to Point#2:

- In the Introduction, the fourth paragraph, we revised the statement

"To address these diagnostic issues, we employed data harmonization..., and few-shot learning, to improve generalization."

to:

"To address these issues, we evaluated several discrepancy mitigation strategies, including data harmonization, correlation removal, kernel mean matching, semi-supervised domain adaptation, and the proposed RegAlign. Unlike the comparator methods, RegAlign was designed for few-shot labeled target adaptation in clinically imbalanced settings by jointly combining source-side focal learning, target-side class-weighted supervision, and class-conditional alignment."

- In the Proposed RegAlign subsection, we added the following statements at the end of the section:

"RegAlign differs from the comparator methods in both supervision setting and optimization target. Correlation remover operates at the feature level by reducing linear dependence on sensitive attributes, while kernel mean matching reweights source samples to better match the target marginal distribution. The SSDA baseline instead relies on entropy minimization and pseudo-label-based alignment on unlabeled target data. In contrast, RegAlign is designed for few-shot labeled target adaptation and explicitly combines source-side focal learning, target-side class-weighted supervision, and class-conditional alignment within a single objective. This formulation is intended to improve adaptation to underrepresented populations in clinically imbalanced settings."

- In the Discussion section, we revised the paragraph describing the contribution of RegAlign to further emphasize its novelty relative to existing methods as follows:

"To mitigate these disparities, we benchmarked correlation removal, kernel mean matching, semi-supervised domain adaptation, and the proposed RegAlign. RegAlign is a regularized few-shot adaptation objective designed for clinically imbalanced settings. Its contribution is not simply that it aligns source and target data, but that it jointly addresses three challenges common in underrepresented populations: source-domain imbalance, sparse labeled target samples, and cross-group prediction mismatch. In this respect, RegAlign differs from feature-level correction methods such as correlation remover, reweighting-based approaches such as kernel mean matching, and entropy-based semi-supervised adaptation on unlabeled target data. Empirically, it provided the most consistent improvement in the balance between predictive performance and inter-group parity across training configurations (Results - Trade-off Between Accuracy and Fairness)."
