# Supplementary material for: Advancing fair and explainable machine learning for neuroimaging dementia pattern classification in multi-racial and multi-ethnic populations
Source: Nat Commun. 2026 Jun 26;17:8026. doi: 10.1038/s41467-026-74515-w (PMC13454470; doi:10.1038/s41467-026-74515-w)
Supplement: Supplementary file 2 [file 41467_2026_74515_MOESM1_ESM.pdf]

Supplementary Information for

Advancing Fair and Explainable Machine Learning for Neuroimaging Dementia Pattern Classification in Multi-Racial and Multi-Ethnic Populations

Contents

Experimental settings **S1**

Ablation Studies **S3**

Unharmonized vs. Harmonized Performance S3

Other Machine Learning Classifier Performance S5

Additional Results S5

Statistical evidence of cross-ethnic discrepancies S5

Performance on Discrepancy Mitigation Techniques S6

Ablation of RegAlign loss components S7

Performance on External Datasets S8

Sensitivity Analysis via Top-K Robustness S9

SHAP-based Heatmap Visualization Guided by ALE Meta-analysis S9

SHAP-Based Partial Dependence Analysis of 144 Brain Regions S11

List of Figures

1 Data splitting across seven training scenarios S12

2 Comparison of unharmonized and harmonized baseline results across training scenarios . S13

3 Performance and pairwise ethnic gaps across training scenarios using alternative classifiers (SVM and MLP) S14

4 Method comparison for three scenarios originally trained on NHW / NHA / HISP S16

5 Performance and gaps of RegAlign loss components S17

6 External validation on ADNI+STAC (1/2) S18

7 External validation on ADNI+STAC (2/2) S19

8 Pareto-front analysis of the trade-off between accuracy and fairness S20

9 Top-K robustness sensitivity (normalized) S22

10 ALE-guided SHAP brain-map visualization across training scenarios S23

11 Partial dependence plots for all 144 brain regions S28

List of Tables

1 Default and tuning parameters for hyperparameter optimization S1

2 Sample sizes used in domain adaptation settings S4

3 Statistical tests for pairwise ethnic gaps S15

4 Top-K robustness sensitivity analysis across methods and training scenarios S21

Experimental Settings

Hyperparameter tuning was performed using GridSearchCV with 10-fold cross-validation loop to optimize the performance of the XGBoost classifier. This approach systematically evaluated all combinations of specified hyperparameter values to identify the configuration that achieved the best classification results. Supplementary Table 1 details the default settings and the search ranges used for optimization in this study.

Supplementary Table 1: Default and tuning parameters for hyperparameter optimization. Default values serve as the initial settings, while the tuning grid defines the range of values explored during GridSearchCV with 10-fold cross-validation.

**Classifier Models**				
**Model**	**Parameter**	**Default**	**Tuning values**	**Description**
XGBoost	`max_depth`	2	[2, 3, 4, 5, 6]	Maximum depth of a tree; controls model complexity and overfitting.
	`learning_rate`	1	[0.1, 0.3, 0.5, 0.7, 1]	Step size shrinkage to prevent overfitting and improve convergence.
	`lambda`	1	[1, 3, 5, 7]	L2 regularization term on weights to reduce overfitting.
	`alpha`	0	[0, 1, 3, 5]	L1 regularization term on weights to encourage sparsity.
SVM	`C`	1	[0.001, 0.01, 0.1, 1, 10]	Regularization parameter; controls the trade-off between margin width and classification error.
	`kernel`	`rbf`	{`rbf`, `linear`}	Specifies the kernel type used in the algorithm.
MLP	`hidden_layer_sizes`	64	[64, 128, 256]	Number of hidden layer nodes.
	`alpha`	`1e-5`	[`1e-5`, `1e-4`, `1e-3`]	Regularization parameter.
	`learning_rate_init`	`1e-4`	[`1e-4`, `5e-4`, `1e-3`]	Initial learning rate.
**Discrepancy Mitigation Methods**				
CR	`cr_alpha`	0.01	[0.01, 0.025, 0.05, 0.075, 0.1, 0.25, 0.5, 0.75, 1.0]	Correlation remover strength; controls the extent of feature–covariate decorrelation.
KMM	`kmm_B`	512	[512.0, 1000.0, 2000.0]	Upper bound on the reweighting coefficients (controls weight magnitude).
	`kmm_eps`	0.05	[`None`, 0.05, 0.10]	Slack for sum-of-weights constraint (stability/regularization).
	`kmm_max_size`	500	[500, 1000, 2000]	Maximum subsample size used to estimate KMM weights (computational control).
	`kmm_gamma`	0.25	[`median`, 0.25]	RBF kernel bandwidth parameter (fixed, median heuristic, or numeric).

(continued on next page)

Method	Parameter	Default	Tuning values	Description
	kmm_scale	0.5	[0.5, 1.0]	Global scaling applied to KMM weights (controls reweighting intensity).
SSDA RegAlign	tau	0.25	[0.25, 0.5, 0.75, 1.0]	Scale factor for target-domain contribution.
	source_focal_gamma	1.0	[1.0, 2.0, 4.0]	Focal-loss focusing parameter for the source domain.
	target_focal_gamma	1.0	[0.0, 2.0, 4.0]	Focal-loss focusing parameter for the target domain.
	target_weight	1.0	[1.0, 1.25, 1.5]	Positive-class weight for target-domain loss.
	align_lambda	0.1	[0.1, 0.2, 0.3, 0.4, 0.5]	Weight of the domain-alignment component.

Training procedure followed three complementary strategies to evaluate model performance and fairness across populations, as illustrated in Supplementary Supplementary Figure 1. In the first strategy, the full dataset, including all racial and ethnic groups, was evaluated using a Monte Carlo cross-validation framework with 100 independent runs. In each run, data were stratified by diagnostic group and ethnicity and randomly split into 80% training/validation and 20% held-out test sets to estimate generalization performance. Hyperparameter optimization was conducted exclusively within the 80% training/validation subset using inner 10-fold cross-validation (GridSearchCV), exploring 400 parameter combinations for XGBoost, 10 for SVM, and 27 for MLP. Following selection of the optimal configuration in each run, the model was refitted on the full 80% subset and evaluated on the corresponding 20% held-out test set as well as the remaining external set. Repeating this process across 100 runs obtained robust performance estimates while accounting for sampling variability and population heterogeneity.

In the second strategy, training was restricted to a single population group, after which the same Monte Carlo cross-validation procedure was applied. Specifically, data from the selected population were stratified by diagnosis and repeatedly split into 80% training/validation and 20% test sets across 100 runs, with inner 10-fold cross-validation used for hyperparameter tuning within each run. Evaluation was performed not only on the held-out test subset from the same population but also on data from all other populations that were excluded during training. This design enabled systematic assessment of cross-population generalization and out-of-distribution performance. Similar data splitting strategy is applied that training was restricted to two population groups and tested on the remaining group. In general, these three strategies provide a comprehensive characterization of both overall model behavior and population-specific fairness under repeated resampling.

Data Acquisition for Domain Adaptation. We defined domain adaptation splits differently for the instance-based KMM approach and the objective-based SSDA/RegAlign approaches, while keeping the downstream training scenarios consistent across methods. All domain adaptation components were constructed using the full cohorts ($n = 9,007$ subjects), with ethnic composition 73.1% NHW, 14.0% NHA, 7.9% HISP, and 5.0% Other. The number for source and target samples for each method is shown in Supplementary Table 2.

Kernel Mean Matching (KMM) was implemented as an instance-based reweighting strategy, where

sample weights were computed prior to model training and then held fixed during classifier fitting. For each ordered source–target ethnicity pair, we fit a KMM model using the source-group training samples as the source domain and the target-group samples as the target domain, yielding nine pre-computed weight sets, denoted as $w_{\text{source}\to\text{target}}$: $w_{\text{NHW}\to\text{NHA}}$, $w_{\text{NHW}\to\text{HISP}}$, $w_{\text{NHA}\to\text{NHW}}$, $w_{\text{NHA}\to\text{HISP}}$, $w_{\text{HISP}\to\text{NHW}}$, $w_{\text{HISP}\to\text{NHA}}$, $w_{\text{NHW}\to\text{Other}}$, $w_{\text{NHA}\to\text{Other}}$, and $w_{\text{HISP}\to\text{Other}}$. These weight sets were then mapped to each training scenario as follows. When training on ALL groups, we applied weights only to NHW, using $w_{\text{NHW}} = \max(w_{\text{NHW}\to\text{NHA}}, w_{\text{NHW}\to\text{HISP}})$, reflecting that NHW constituted the largest fraction of the cohort and thus served as the primary source distribution; for NHA and HISP, weights were set to the median of NHW weights. When training on two groups (e.g., NHW+NHA), we weighted each included source group toward the held-out group (e.g., HISP) using $w_{\text{NHW}} = w_{\text{NHW}\to\text{HISP}}$ and $w_{\text{NHA}} = w_{\text{NHA}\to\text{HISP}}$; similar assignments were used for NHW+HISP (target NHA) and NHA+HISP (target NHW). When training on a single group (e.g., NHW only), we weighted the training samples toward the remaining groups via $w_{\text{NHW}} = \max(w_{\text{NHW}\to\text{NHA}}, w_{\text{NHW}\to\text{HISP}})$, and similarly for NHA and HISP. For experiments involving the Other group, we used the corresponding pre-computed mappings ($w_{\text{NHW}\to\text{Other}}$, $w_{\text{NHA}\to\text{Other}}$, and $w_{\text{HISP}\to\text{Other}}$) to refit KMM and generate scenario-matched weights.

SSDA and RegAlign were implemented as objective-based domain alignment approaches, where source and target domains were explicitly defined and jointly optimized during training. For the ALL-training scenario, the source domain consisted of the training split from all ethnic groups, while the target domain was defined as the held-out test split. This setup operationalizes domain alignment with respect to the evaluation distribution, such that the alignment objective is optimized against samples that are not used to update the classifier parameters in the supervised loss, while still reflecting the deployment/evaluation distribution. For two-group training scenarios (e.g., NHW+NHA), the source domain comprised the training samples from the included groups (NHW and NHA), and the target domain comprised the remaining group (HISP); analogous definitions were used for NHW+HISP (target NHA) and NHA+HISP (target NHW). For single-group training scenarios (e.g., NHW only), the source domain was the training set of the chosen group (NHW), and the target domain was formed by pooling the remaining groups (NHA and HISP); similarly, for NHA-only and HISP-only training, the target included the other two groups. For external testing and analyses involving the Other ethnic group, the source domain definition remained unchanged for each scenario, and the target domain was expanded to include the corresponding external samples and/or the Other group, ensuring that the alignment objective explicitly incorporated the distributional shift relevant to the evaluation setting.

Ablation Studies

Unharmonized vs. Harmonized Baseline Results

Supplementary Figure 2 compares baseline performance and pairwise racial/ethnic gaps between unharmonized (U) and harmonized (H) features across training scenarios. For balanced accuracy, shown in Supplementary Figure 2a, harmonization increased NHW BA under ALL training from 79.62 ± 5.77

Supplementary Table 2: Sample sizes used in domain adaptation settings for KMM, SSDA, and RegAlign. n_{per} is the number of randomly picked samples per ethnic group per diagnosis.

(a) KMM

**KMM**					
Source→Target	N_{source}:N_{target}	Source→Target	N_{source}:N_{target}	Source→Target	N_{source}:N_{target}
NHW→NHA	6584:1263	NHA→HISP	1263:713	NHW→Other	6584:447
NHW→HISP	6584:713	HISP→NHW	713:6584	NHA→Other	1263:447
NHA→NHW	1263:6584	HISP→NHA	713:1263	HISP→Other	713:447

(b) SSDA

<table>
<tr><th colspan="9">SSDA</th></tr>
<tr><td colspan="2">Scenario</td><td>ALL</td><td>NHW+NHA</td><td>NHW+HISP</td><td>NHA+HISP</td><td>NHW</td><td>NHA</td><td>HISP</td></tr>
<tr><td colspan="2">N_{source}</td><td>681</td><td colspan="3">454</td><td colspan="3">227</td></tr>
<tr><td rowspan="2">N_{target}</td><td>test</td><td>57 NHW
57 NHA
57 HISP</td><td>284 HISP</td><td>284 NHA</td><td>284 NHW</td><td>284 NHA
284 HISP</td><td>284 NHW
284 HISP</td><td>284 NHW
284 NHA</td></tr>
<tr><td>external (n_{per}=50)</td><td>200 NHW
151 NHA
150 HISP</td><td>150 HISP</td><td>151 NHA</td><td>200 NHW</td><td>151 NHA
150 HISP</td><td>200 NHW
150 HISP</td><td>200 NHW
151 NHA</td></tr>
</table>

(c) RegAlign

<table>
<tr><th colspan="9">RegAlign</th></tr>
<tr><td colspan="2">Scenario</td><td>ALL</td><td>NHW+NHA</td><td>NHW+HISP</td><td>NHA+HISP</td><td>NHW</td><td>NHA</td><td>HISP</td></tr>
<tr><td colspan="2">N_{source}</td><td>681</td><td colspan="3">454</td><td colspan="3">227</td></tr>
<tr><td rowspan="2">N_{target}</td><td>test (n_{per}=5)</td><td>10 NHW
10 NHA
10 HISP</td><td>10 HISP</td><td>10 NHA</td><td>10 NHW</td><td>10 NHA
10 HISP</td><td>10 NHW
10 HISP</td><td>10 NHW
10 NHA</td></tr>
<tr><td>external (n_{per}=5)</td><td>20 NHW
20 NHA
15 HISP</td><td>15 HISP</td><td>20 NHA</td><td>20 NHW</td><td>20 NHA
15 HISP</td><td>20 NHW
15 HISP</td><td>20 NHW
20 NHA</td></tr>
</table>

(U) to 84.51 ± 4.69 (H), while BA was similar for NHA (80.49 ± 6.27 vs. 80.27 ± 4.74) and decreased slightly for HISP (80.39 ± 5.78 vs. 79.36 ± 4.32). Correspondingly, the NHW–NHA BA gap shifted from -0.87 ± 9.05 (U) to 4.24 ± 5.85 (H), and the NHW–HISP BA gap shifted from -0.77 ± 9.09 (U) to 5.14 ± 5.25 (H). For false negative rate, shown in Supplementary Figure 2b, harmonization reduced NHW FNR under ALL training from 16.82 ± 8.15 to 10.39 ± 6.04, but increased FNR for NHA (23.94 ± 9.46 to 26.62 ± 8.98) and HISP (21.85 ± 7.79 to 23.34 ± 8.23). As a result, the NHW–NHA FNR gap increased in magnitude from -7.12 ± 13.28 to -16.23 ± 8.35, and the NHW–HISP FNR gap increased from -5.03 ± 11.10 to -12.96 ± 9.29. For false positive rate, shown in Supplementary Figure 2c, harmonization reduced FPR for NHW (23.95 ± 8.24 to 20.60 ± 6.93) and NHA (15.08 ± 7.68 to 12.84 ± 5.62), with a smaller change for HISP (17.37 ± 7.08 to 17.93 ± 7.02). This decreased the NHW–HISP FPR gap from 6.57 ± 11.82 to 2.67 ± 8.66, while the NHW–NHA FPR gap remained similar

(8.86 ± 11.00 vs. 7.76 ± 8.15).

Presence of Discrepancy among Multi-Ethnic Dementia Classification using Other Machine Learning Classifiers

Supplementary Figure 3 summarizes dementia classification performance and pairwise racial/ethnic gaps using two additional classifiers, SVM and MLP, across multiple training scenarios. For balanced accuracy, shown in Supplementary Figure 3a, performance under ALL training remained broadly comparable across groups for both SVM (NHW 81.65 ± 4.93, NHA 80.11 ± 4.70, HISP 81.12 ± 4.91) and MLP (NHW 80.79 ± 4.63, NHA 79.67 ± 4.78, HISP 79.81 ± 4.67). These results indicate that overall classification accuracy was relatively similar across groups when all populations were included during training. For false negative rate, shown in Supplementary Figure 3b, error-rate disparities were still evident. Under ALL training, NHW participants had lower FNR than NHA participants for both SVM (NHW 16.43 ± 6.82 vs. NHA 23.70 ± 7.45; NHW–NHA FNR gap -7.28 ± 9.60) and MLP (NHW 17.66 ± 6.77 vs. NHA 25.26 ± 7.83; NHW–NHA FNR gap -7.60 ± 9.67). Under HISP-only training, NHW–NHA FNR gaps also remained negative for both SVM (-6.45 ± 2.06) and MLP (-5.99 ± 2.56), indicating persistent differences in missed-diagnosis rates across groups. For false positive rate, shown in Supplementary Figure 3c, ALL training produced positive NHW–NHA FPR gaps for both classifiers (SVM: 4.20 ± 9.13; MLP: 5.38 ± 8.58). Discrepancies were more pronounced under NHA-only training, where both classifiers showed higher FPR for NHW than for NHA participants (SVM: NHW 36.66 ± 3.82 vs. NHA 19.35 ± 7.65, NHW–NHA FPR gap 17.31 ± 7.21; MLP: NHW 33.91 ± 4.06 vs. NHA 17.40 ± 7.12, NHW–NHA FPR gap 16.51 ± 7.06).

Additional Discrepancy Mitigation Results

Statistical evidence of cross-ethnic discrepancies

To formally test whether performance discrepancies differed from zero across racial/ethnic groups, we conducted two-sided t-tests on the distribution of pairwise gaps for BA, FNR, and FPR, as shown in Supplementary Table 3. Under the NHW-trained setting, significant gaps were observed for NHW–NHA comparisons in BA (3.76, 95% CI [2.93, 4.64], $p = 1.41 \times 10^{-13}$), FNR (-14.65, 95% CI [-15.77, -13.54], $p = 3.02 \times 10^{-45}$), and FPR (7.13, 95% CI [5.75, 8.49], $p = 6.81 \times 10^{-18}$). Similar significant gaps were observed for NHW–HISP comparisons in BA (4.16, 95% CI [3.35, 4.99], $p = 3.38 \times 10^{-16}$), FNR (-14.91, 95% CI [-15.98, -13.79], $p = 4.79 \times 10^{-46}$), and FPR (6.58, 95% CI [5.30, 7.78], $p = 5.49 \times 10^{-18}$).

Under the NHA-trained setting, the largest discrepancy was observed for the NHW–NHA FPR gap (20.26, 95% CI [19.13, 21.33], $p = 5.66 \times 10^{-58}$), with additional significant gaps for NHW–NHA BA (-5.59, 95% CI [-6.45, -4.70], $p = 5.10 \times 10^{-22}$) and FNR (-9.07, 95% CI [-10.60, -7.48], $p = 7.90 \times 10^{-20}$). Under the HISP-trained setting, significant discrepancies were also observed, including NHW–HISP BA (-4.57, 95% CI [-5.50, -3.63], $p = 1.09 \times 10^{-15}$), FNR (-1.76, 95% CI [-3.31, -0.24], $p = 2.92 \times 10^{-2}$), and FPR (10.89, 95% CI [9.76, 12.06], $p = 2.84 \times 10^{-34}$).

In the pooled ALL-trained setting, NHW–NHA and NHW–HISP comparisons remained significant for BA, FNR, and FPR. For example, NHW–NHA gaps were significant for BA (4.24, 95% CI [3.10, 5.36], $p = 9.86 \times 10^{-11}$), FNR (-16.23, 95% CI [-17.78, -14.58], $p = 1.47 \times 10^{-35}$), and FPR (7.76, 95% CI [6.18, 9.31], $p = 1.19 \times 10^{-15}$). NHW–HISP gaps were also significant for BA (5.14, 95% CI [4.12, 6.17], $p = 2.96 \times 10^{-16}$), FNR (-12.96, 95% CI [-14.85, -11.14], $p = 4.19 \times 10^{-25}$), and FPR (2.67, 95% CI [1.01, 4.39], $p = 2.63 \times 10^{-3}$). Some comparisons were not statistically significant, including NHA–HISP FNR in the NHW-trained setting (-0.25, 95% CI [-0.86, 0.44], $p = 0.44$) and NHA–HISP BA in the ALL-trained setting (0.90, 95% CI [-0.24, 2.09], $p = 0.14$). Overall, these results indicate that performance and error-rate discrepancies persist across multiple training scenarios, with the largest gaps often observed in FNR and FPR.

Performance on Discrepancy Mitigation Techniques

To contextualize the benefit of *RegAlign*, we compared it against three representative discrepancy-mitigation baselines: cost re-weighting (*CR*), kernel mean matching (*KMM*), and semi-supervised domain adaptation (*SSDA*). Results are reported for three single-population training scenarios (NHW-, NHA-, and HISP-only training), evaluated on all ethnic groups using (i) group-wise performance (BA, FNR, FPR) and (ii) pairwise discrepancy gaps (NHW–NHA, NHW–HISP, NHA–HISP), as shown in Supplementary Figure 4. Complete comparison are provised in Supplementary Data 1 and 2. Statistical significance for improvements over *Baseline* was assessed using two-sided t-tests across repeated runs (see Supplementary Data 3 and 4), where we report the corresponding p-values for key comparisons.

Originally training on NHW. When models were trained on NHW only, *RegAlign* delivered the strongest overall accuracy and sensitivity improvements across ethnicities, increasing the mean BA (averaged over NHW/NHA/HISP) from 79.14% (*Baseline*) to 82.67% and reducing the mean FNR from 29.24% to 16.12%. Most notably, *RegAlign* substantially improved BA for underrepresented groups: NHA BA increased from 77.56% $\pm$ 3.22% to 81.12% $\pm$ 1.89% ($p < 10^{-99}$), and HISP BA increased from 76.75%$\pm$3.76% to 82.20%$\pm$1.92% ($p < 10^{-99}$). These accuracy gains were accompanied by large reductions in missed-diagnosis rates: NHA FNR decreased from 34.13% $\pm$ 10.66% to 20.19% $\pm$ 5.33% ($p < 10^{-99}$) and HISP FNR decreased from 35.72% $\pm$ 12.58% to 17.61% $\pm$ 5.03% ($p < 10^{-99}$). In terms of discrepancy mitigation, *RegAlign* meaningfully reduced error-rate gaps, shrinking the mean absolute FNR gap across pairs from 17.09% to 8.35% and the mean absolute FPR gap from 9.58% to 7.41%. At the pair level, the magnitude of the NHW–NHA FNR gap decreased from 16.24% $\pm$ 3.89% to 9.64% $\pm$ 6.09% ($p < 10^{-99}$), and the NHW–HISP FNR gap decreased from 17.84% $\pm$ 5.11% to 7.06% $\pm$ 5.30% ($p < 10^{-99}$). Overall, among the compared methods, *RegAlign* achieved the best joint outcome of higher BA on underrepresented groups and lower (FNR/FPR) disparity, whereas alternative techniques (CR/KMM/SSDA) tended to improve only one aspect (either BA or gaps) without matching the combined gains.

Originally training on NHA. Under NHA-only training, *RegAlign* again produced the best average discrimination, increasing mean BA from 75.86% (*Baseline*) to 80.62% and reducing mean FNR from 25.46% to 12.83%. The largest performance gains were observed for HISP, where BA improved from 73.55% ± 4.42% to 78.89% ± 2.02% ($p < 10^{-59}$), and FNR dropped from 28.71% ± 7.57% to 13.77% ± 4.07% ($p < 10^{-78}$). For fairness, *RegAlign* substantially improved FPR parity in this setting: the mean absolute FPR gap decreased from 13.79% to 9.01% (the lowest among methods), and the mean absolute FNR gap decreased from 11.80% to 7.19%. For example, the NHW–NHA FPR gap magnitude decreased from 20.25% ± 7.25% to 16.18% ± 5.12% ($p < 10^{-8}$), and the NHA–HISP FPR gap magnitude decreased from 10.99% ± 5.69% to 6.43% ± 4.62% ($p < 10^{-9}$). While *KMM* achieved slightly smaller BA-gap averages in this scenario, *RegAlign* provided the most consistent reductions in clinically relevant error-rate disparities (FNR/FPR) while simultaneously improving BA.

Originally training on HISP. With HISP-only training, *RegAlign* maintained the best overall BA while improving error-rate parity. Mean BA increased from 79.82% (*Baseline*) to 82.57% and mean FNR decreased from 20.05% to 13.01%. The strongest accuracy improvement was for NHA: BA increased from 76.69% ± 3.45% to 79.40% ± 2.91% ($p < 10^{-9}$), with a corresponding FNR reduction from 18.95% ± 5.38% to 11.32% ± 4.67% ($p < 10^{-20}$). Regarding discrepancies, *RegAlign* yielded the lowest mean absolute FPR gap in this setting (from 6.19% under *Baseline* to 5.50%), while keeping FNR disparity comparable to *Baseline* (mean absolute FNR gap 4.92% vs. 5.10%). At the pair level, the NHW–NHA FPR gap magnitude decreased from 7.17% ± 4.54% to 6.27% ± 3.97% ($p = 0.006$), and the NHA–HISP FPR gap magnitude decreased from 5.39% ± 3.54% to 4.37% ± 2.93% ($p = 0.003$). Thus, even when trained on the smallest single-population cohort, *RegAlign* improved cross-group generalization (especially for NHA) and reduced false-positive disparities more consistently than competing discrepancy-mitigation baselines.

Summary. Across NHW-, NHA-, and HISP-only training scenarios, *RegAlign* provided the most reliable joint improvement in (i) accuracy on underrepresented groups and (ii) reduction of the most consequential error-rate gaps (particularly FNR and FPR), compared with *CR*, *KMM*, and *SSDA*. Complete results are provided in Supplementary Data 1 – 4.

Ablation of RegAlign loss components

We next investigated the contribution of the loss components used in RegAlign. In all settings, the **source-domain classification loss** $\mathcal{L}_{\text{cls}}^{S}$ is the essential objective to preserve predictive performance on the labeled training distribution. We then progressively add two **optional adaptation terms**: (i) a **target-domain classification loss** $\mathcal{L}_{\text{cls}}^{T}$ (applied on the target stream used during adaptation) and (ii) a **class-conditional alignment loss** $\mathcal{L}_{\text{align}}^{S\leftrightarrow T}$, to quantify how each addition affects both accuracy and cross-ethnic disparities. This results three ablation configurations: (i) **Focal loss**: $\mathcal{L}_{\text{cls}}^{S} + \mathcal{L}_{\text{cls}}^{T}$; (ii) **Alignment**: $\mathcal{L}_{\text{cls}}^{S}+\mathcal{L}_{\text{align}}^{S\leftrightarrow T}$; and (iii) **Focal loss + Alignment**: $\mathcal{L}_{\text{cls}}^{S}+\mathcal{L}_{\text{cls}}^{T}+\mathcal{L}_{\text{align}}^{S\leftrightarrow T}$. Supplementary Figure 5 summarizes

group-stratified performance (top row) and mean pairwise gaps (bottom row) across training scenarios (ALL, NHW, NHA, HISP).

Across scenarios, **Focal loss + Alignment provides the most favorable accuracy–fairness trade-off**. Under ALL-training, it yields the strongest discrimination, increasing mean BA from approximately $\sim 82.6\%$ (Focal loss) and $\sim 83.9\%$ (Alignment) to $\sim 85.0\%$ (Focal loss + Alignment), while simultaneously reducing FNR (from $\sim 17.6\%$ and $\sim 16.1\%$ to $\sim 13.9\%$). It also achieves the **lowest average disparity** for missed dementia cases: the mean pairwise FNR gap decreases from $\sim 12\%$ (Focal loss) to $\sim 10\%$ (Alignment) and reaches $\sim 8\%$ with Focal loss + Alignment. A similar reduction is observed for BA gaps (e.g., under ALL-training, from $\sim 5.5\%$ to $\sim 2.7\%$).

For false positives, the improvement is smaller and more scenario-dependent. Under ALL-training, Focal loss + Alignment slightly lowers mean FPR (from $\sim 17.2\%$ to $\sim 16.1\%$) and reduces the mean pairwise FPR gap (from $\sim 9\%$ to $\sim 6\%$). However, under NHA-only training, the FPR gap increases for the combined objective (green curve in the bottom-right panel of Supplementary Figure 5), highlighting residual challenges in controlling false-positive disparities in the most data-limited regime. Nonetheless, the combined objective remains the most consistent choice for jointly improving discrimination and reducing the largest FNR disparities across scenarios.

Overall, these ablations motivate our final design: **starting from the essential source classification objective and augmenting it with target-aware classification and class-conditional alignment produces the best-performing and most effective configuration**. In particular, **Focal loss + Alignment** consistently improves balanced accuracy and obtains the smallest cross-ethnic FNR gaps, aligning with the clinical priority of reducing missed dementia diagnoses while remaining equitable error rates across populations.

Performance on External Datasets

To assess generalization beyond the group-balanced evaluation set, we performed external validation on two independent test sets. (i) **NACC+SCAN** consisted of the remaining subjects not used for the group-balanced subsets, including NHW (3345 CN / 1348 DA), NHA (697 CN), HISP (169 CN), and Other (137 CN / 63 DA). (ii) **ADNI+STAC** served as a second external set with 1389 CN / 942 DA. Sample counts are summarized in Table 1 and Table 2 (Main Manuscript). Supplementary Figure 6 and Supplementary Figure 7 provide representative examples for ADNI+STAC when models are trained on NHW, NHA, or HISP and evaluated across all ethnic groups.

NACC+SCAN (external). On NACC+SCAN, RegAlign yielded the optimal performance gains for the Other group and reduced selected pairwise gaps relative to Baseline. Under NHW training, Other BA increased from 81.40±4.77 to 87.80±2.50 ($p < 0.001$), and under NHA training, from 80.69±4.88 to 88.49±1.74 ($p < 0.001$). In addition, RegAlign reduced the NHW–NHA FPR gap under NHA training from 11.87±4.08 to 9.04±2.53 ($p < 0.001$) and under NHW training from 3.68±3.11 to 1.64±2.68 ($p < 0.001$). For two-group training (NHW+NHA), the NHW–Other FNR gap was also closer to zero

with RegAlign (Baseline -2.72 ± 3.36 vs. RegAlign -0.27 ± 3.11; $p < 0.001$). Full NACC+SCAN external results are provided in Supplementary Data 5.

ADNI+STAC (external). Across single-group training scenarios, *RegAlign* showed consistent improvements over Baseline for non-source populations on ADNI+STAC, with statistically significant gains in balanced accuracy (BA) and reduced cross-group error-rate gaps (all $p < 0.001$). When trained on NHW, RegAlign increased BA for NHA from 73.56 ± 4.29 to 81.48 ± 2.64 and for HISP from 70.62 ± 3.36 to 78.14 ± 2.46 (both $p < 0.001$), while reducing the magnitude of the FNR gaps for NHW–NHA (Baseline -20.84 ± 6.09 vs. RegAlign -11.98 ± 4.44) and NHW–HISP (Baseline -25.04 ± 6.14 vs. RegAlign -18.22 ± 4.36) (both $p < 0.001$). When trained on NHA, RegAlign increased BA for HISP from 70.23 ± 3.57 to 77.93 ± 1.95 ($p < 0.001$) and reduced the NHW–NHA FPR gap from 19.28 ± 5.37 to 14.64 ± 3.05 ($p < 0.001$). When trained on HISP, RegAlign increased BA for NHA from 72.31 ± 3.34 to 78.70 ± 2.72 ($p < 0.001$). Full ADNI+STAC external results (all methods, metrics, and pairwise gaps) are provided in Supplementary Data 6.

Sensitivity Analysis via Top-K Robustness

To quantify sensitivity of hyperparameter selection, we performed a Top-K robustness analysis [1] that contrasts the selection objective score of the top K (20% of total configurations) against the remaining configurations within each method and training scenario. As summarized in Supplementary Table 4, we report the top-K mean with bootstrap confidence interval (CI; 2000 resamples), the mean of the remaining configurations with CI, and the absolute difference $\Delta = \mu_{\text{top-}K} - \mu_{\text{rest}}$. For comparability across scenarios, Supplementary Figure 9 visualizes the normalized separation $\Delta\% = \frac{\mu_{\text{top-}K} - \mu_{\text{rest}}}{\mu_{\text{rest}}} \times 100\%$, where larger values indicate greater dependence on a small subset of top-ranked configurations.

Across scenarios, **Baseline** exhibited consistently larger normalized separations, ranging from 0.7%–1.1% in absolute improvement of the top-K mean over the rest (ALL: 0.755; NHW: 0.799; NHA: 0.676; HISP: 1.142), corresponding to 0.9%–1.5% when expressed relative to the rest mean (Supplementary Figure 9). **KMM** likewise showed elevated sensitivity, with $\Delta\%$ between 0.6% and 1.3% and the largest separation under NHA (Supplementary Table 4; Supplementary Figure 9). In contrast, **CR** and **RegAlign** remained uniformly low across scenarios ($\Delta\%$ approximately 0.0%–0.3%), indicating that the top-ranked configurations were close to the bulk of the hyperparameter space. Especially, **SSDA** showed larger normalized separations (0.8%–1.5%) across scenarios in Supplementary Figure 9, indicating greater top-K versus rest divergence under this analysis setting.

SHAP-based Heatmap Visualization Guided by ALE Meta-analysis

To interpret model decision patterns and probe whether neuroanatomical signatures of dementia vary across racial/ethnic populations, we performed SHAP-based brain-map visualization using TreeSHAP on gray-matter (GM) regional features. To provide a neurobiologically grounded reference, we anchored the interpretation to an Activation Likelihood Estimation (ALE) meta-analysis of dementia-related struc-

tural alterations (top row in Supplementary Figure 10). We then extracted SHAP contributions for the ALE-guided regions and visualized (i) voxel-level projection of regional SHAP aggregates on axial slices and (ii) the mean SHAP magnitude (with 95% CI) for the most influential regions under four training scenarios: ALL, NHW, NHA, and HISP.

Across scenarios, the most salient contributions consistently involved medial temporal structures (hippocampus, parahippocampal gyrus, and amygdala), broadly aligning with the ALE-derived dementia signature. However, the relative ranking and concentration of SHAP contributions differed by training population, indicating that the model relies on distinct anatomical cues depending on the source cohort.

Trained on ALL. When trained on the pooled cohort, the SHAP map highlighted a canonical AD-related pattern, with dominant contributions in medial temporal regions and additional involvement of temporal/parietal cortices. The summary plot shows strong effects in the amygdala and hippocampal/parahippocampal regions, suggesting that aggregating populations yields a mixed but largely ALE-consistent signature.

Trained on NHW. The NHW-trained model exhibited the closest correspondence to the ALE reference, with comparatively balanced contributions across hippocampal and parahippocampal regions and moderate amygdala effects. This is expected given that much of the historical neuroimaging literature contributing to ALE meta-analyses has been dominated by NHW cohorts; consequently, ALE-guided regions may better reflect the dominant NHW-derived structural dementia signature.

Trained on NHA. In the NHA-trained model, the interpretation shifted toward a more concentrated medial temporal signature, with a pronounced emphasis on hippocampal and amygdala contributions relative to other ALE-guided regions. The corresponding heatmap also appears more focal, suggesting that the classifier leverages a narrower set of regional cues in this cohort. This discrepancy relative to NHW/ALL supports the presence of population-specific decision patterns rather than purely shared, literature-derived signatures.

Trained on HISP. The HISP-trained model showed the strongest dominance of the parahippocampal region among the ALE-guided features, with secondary contributions from the hippocampus and amygdala. Compared with NHW/ALL, this scenario yielded a more skewed attribution profile, indicating that the model's decision boundary relies heavily on a subset of medial temporal structures. Together with NHA, these results reinforce that interpretability can be cohort-dependent: models trained on underrepresented groups may emphasize different anatomical markers (or different weighting of the same markers), motivating domain-adaptation and bias-mitigation strategies that explicitly address cross-population shifts.

Overall, ALE-guided SHAP visualization provides two complementary insights: (i) a shared neuroanatomical backbone consistent with dementia-related medial temporal atrophy, and (ii) systematic

shifts in attribution strength and ranking across training populations, which helps explain why predictive performance and error-rate disparities can persist across ethnic groups even under harmonization.

SHAP-Based Partial Dependence Analysis of 144 Brain Regions

This section presents the full set of partial dependence plots for 144 brain regions, illustrating the relationship between regional brain volume (x-axis) and SHAP contribution (y-axis) across the classification models. Positive SHAP values indicate contributions toward dementia prediction, whereas negative values support normal cognition classification. Panels show partial dependence plots generated from baseline models trained under four scenarios: ALL training (Supplementary Table 1a), NHW training (Supplementary Table 1b), NHA training (Supplementary Table 1c), and HISP training (Supplementary Table 1d). These plots provide insight into how variations in regional brain volume influence model predictions and help identify region-specific volume thresholds associated with increased dementia risk across training populations. The definitional table of the 144 brain regions, ordered from left to right and top to bottom as displayed in the partial dependence plots, is provided in Supplementary Table 1e.

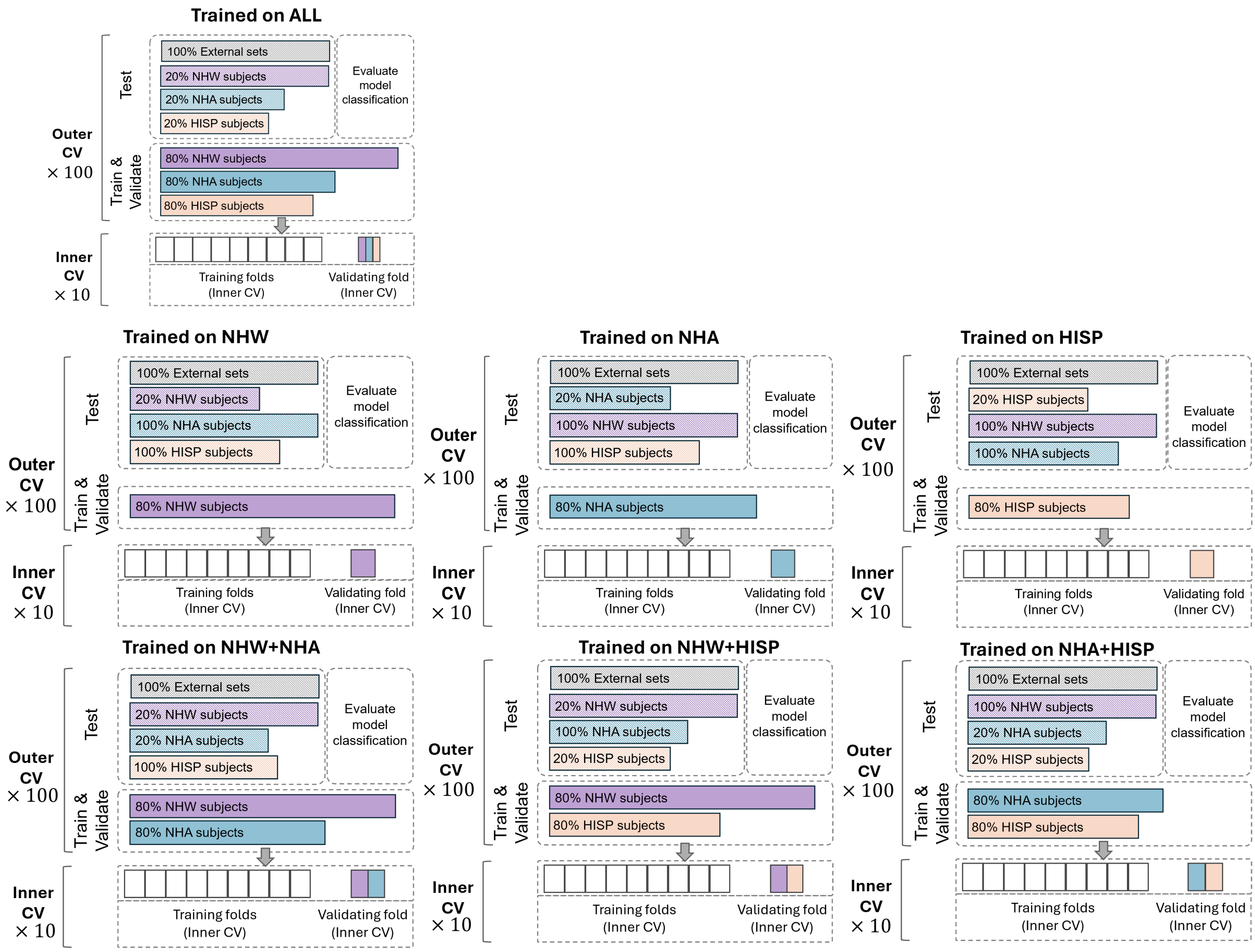

Supplementary Figure 1: Data splitting strategy across seven training scenarios. The schematic illustrates the repeated cross-validation design used to evaluate model performance and inter-group generalization across seven training scenarios: training on all groups (ALL), each single group (NHW, NHA, or HISP), and each two-group combination (NHW+NHA, NHW+HISP, or NHA+HISP). In each scenario, an outer Monte Carlo cross-validation loop was repeated 100 times. Within each outer run, the training population was split into 80% training/validation data and 20% held-out test data where applicable. For models trained on ALL, 80% of NHW, NHA, and HISP participants were used for training/validation, while 20% of each group, together with the external datasets, was used for testing. For single-group training, 80% of the selected group was used for training/validation, while the held-out 20% of that group, the full non-training groups, and the external datasets were used for evaluation. For two-group training, 80% of the selected groups was used for training/validation, while the held-out 20% of the training groups, the full excluded group, and the external datasets were used for testing. Within each outer training/validation set, an inner 10-fold cross-validation loop was used for hyperparameter tuning, with nine folds used for training and one fold used for validation. NHW: non-Hispanic White; NHA: non-Hispanic African; HISP: Hispanic White; CV: cross-validation.

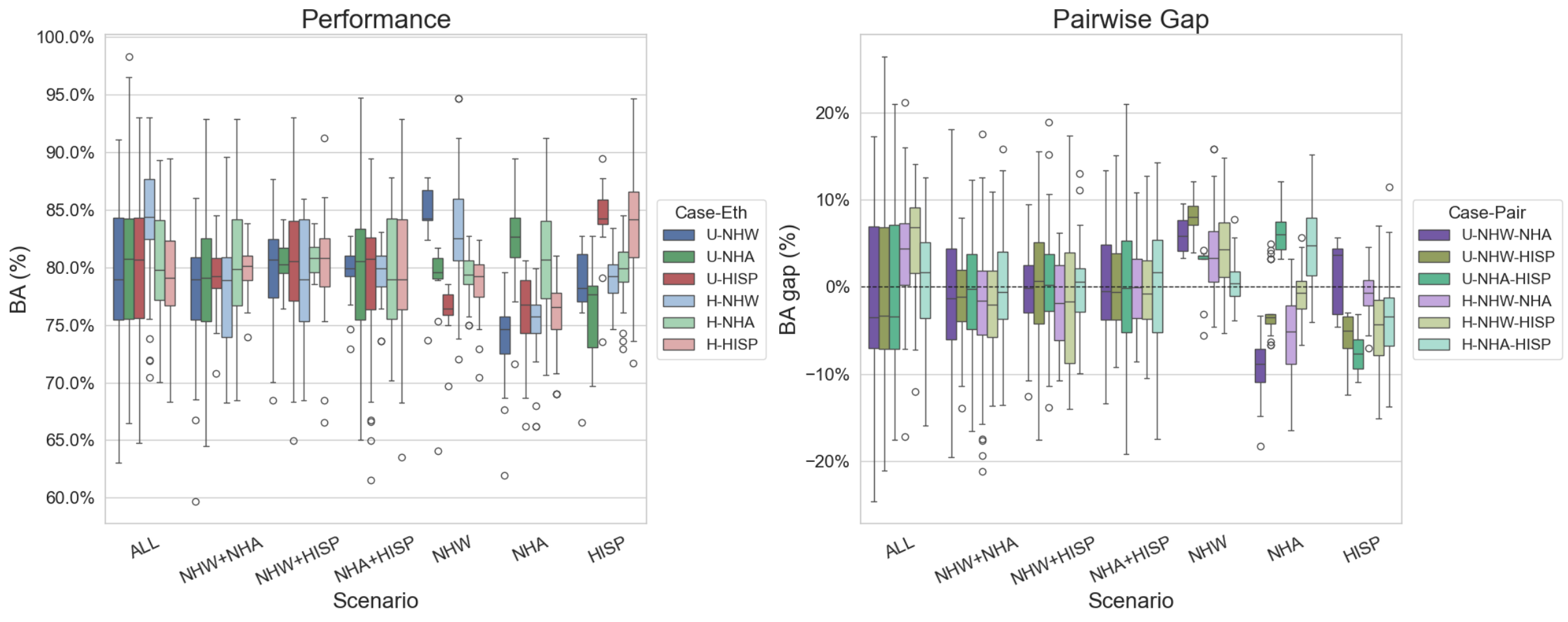

(a) BA. Left: ethnicity-stratified balanced accuracy. Right: pairwise BA gaps.

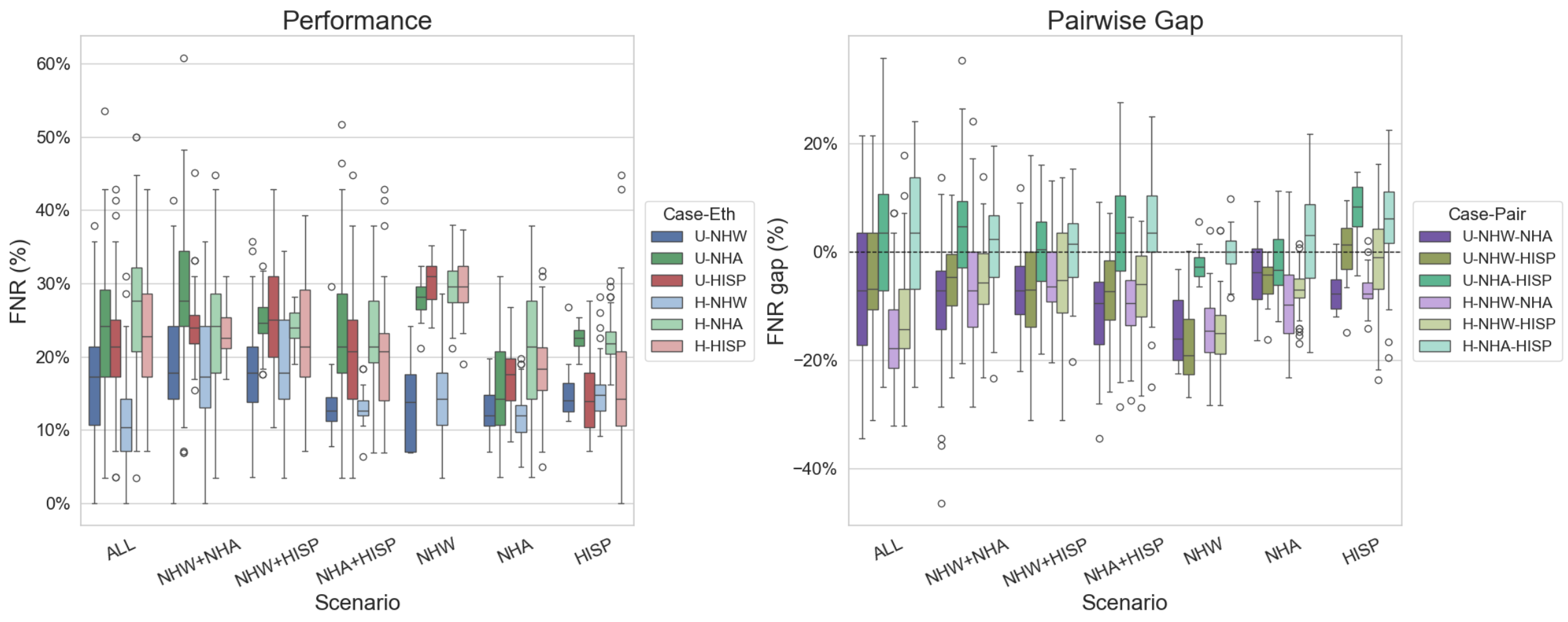

(b) FNR. Left: ethnicity-stratified false negative rate. Right: pairwise FNR gaps.

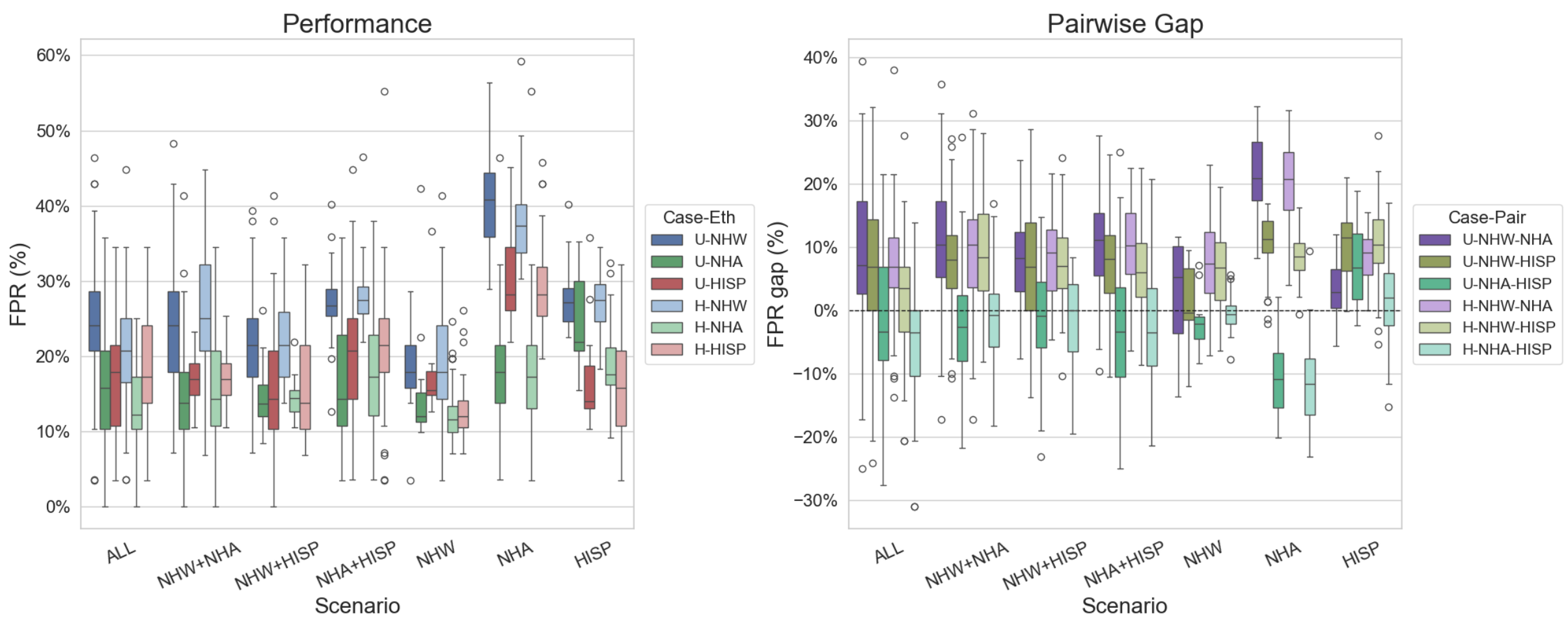

(c) FPR. Left: ethnicity-stratified false positive rate. Right: pairwise FPR gaps.

Supplementary Figure 2: Unharmonized and harmonized baseline performance across training scenarios. Box plots compare unharmonized (U) and harmonized (H) baseline results for seven training scenarios. Panels show (a) balanced accuracy (BA), (b) false negative rate (FNR), and (c) false positive rate (FPR); left plots show ethnicity-stratified performance and right plots show pairwise gaps. Box centers indicate medians, bounds indicate the 25th and 75th percentiles, whiskers extend to non-outlier values within 1.5 times the interquartile range, and points indicate outliers. Pairwise gaps are computed as differences between groups for each metric. Sample sizes are provided in Supplementary Table 2. NHW, non-Hispanic White; NHA, non-Hispanic African American; HISP, Hispanic White. Source data are provided as Source Data 3.

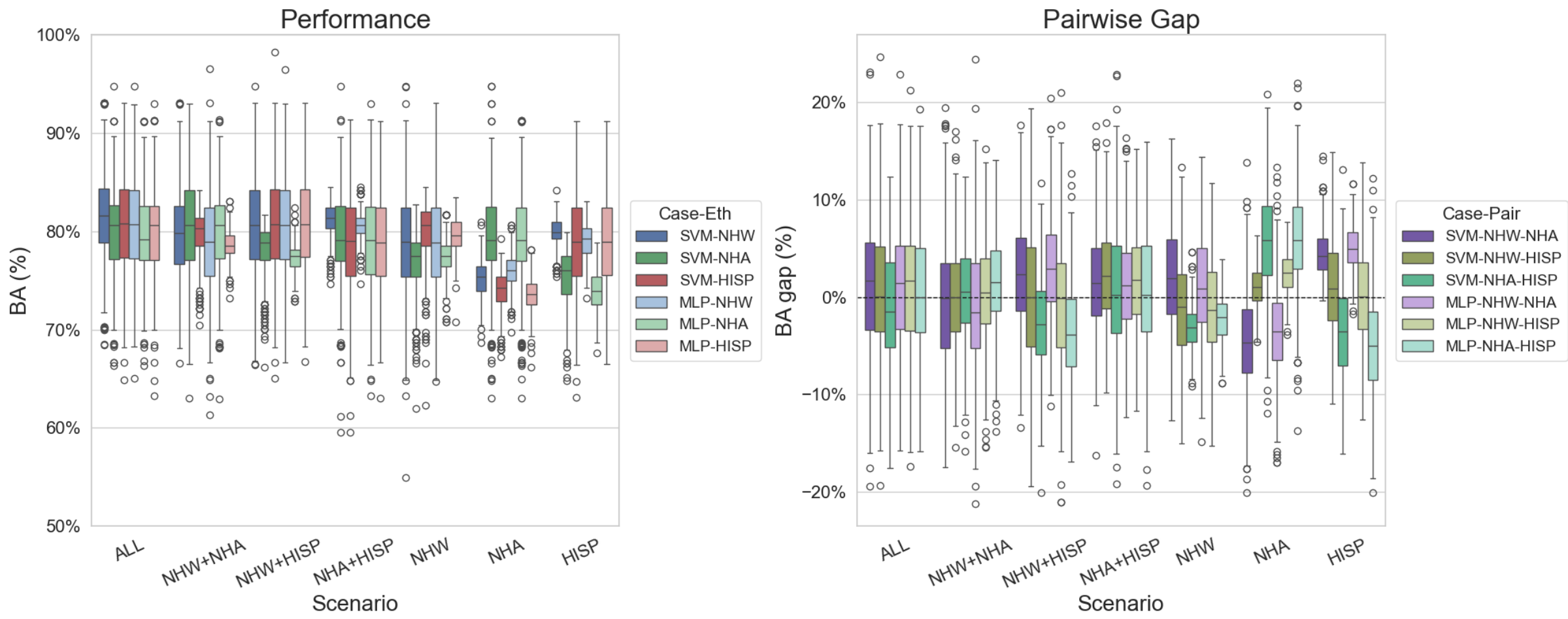

(a) Balanced accuracy (BA) and BA gaps.

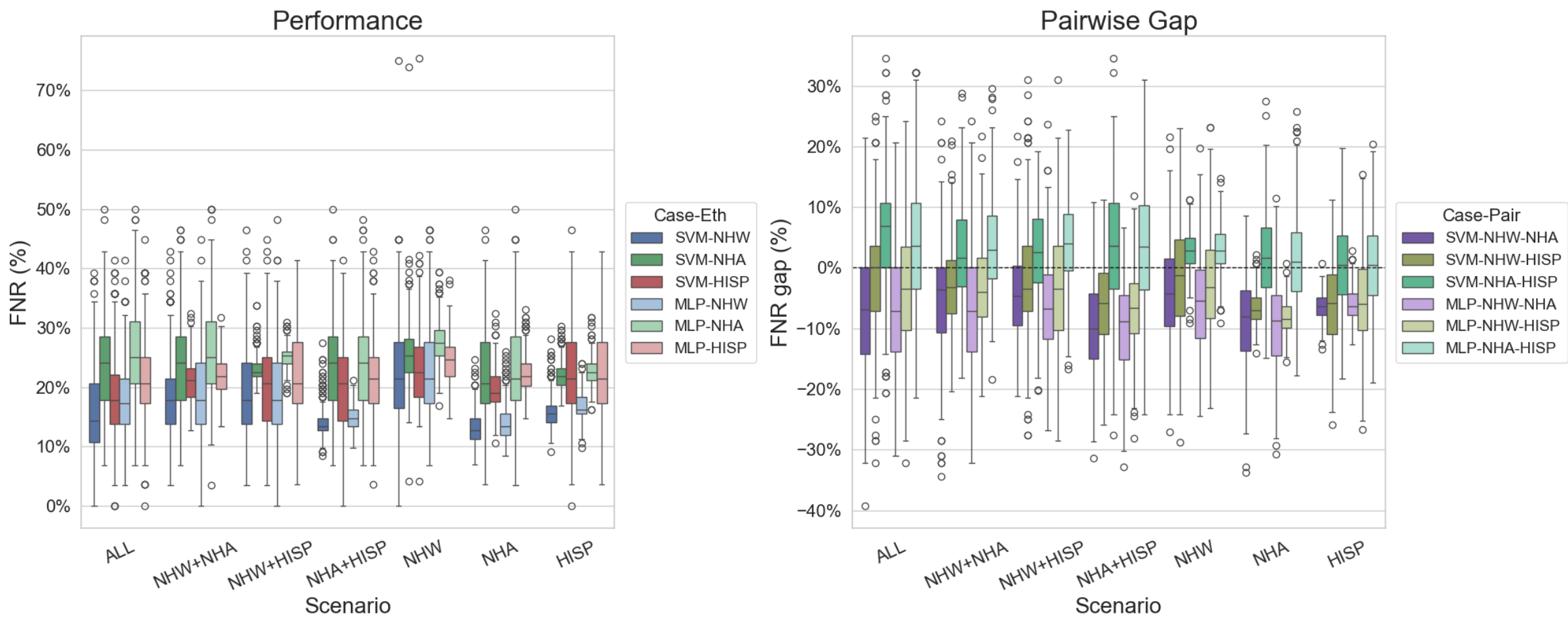

(b) False negative rate (FNR) and FNR gaps.

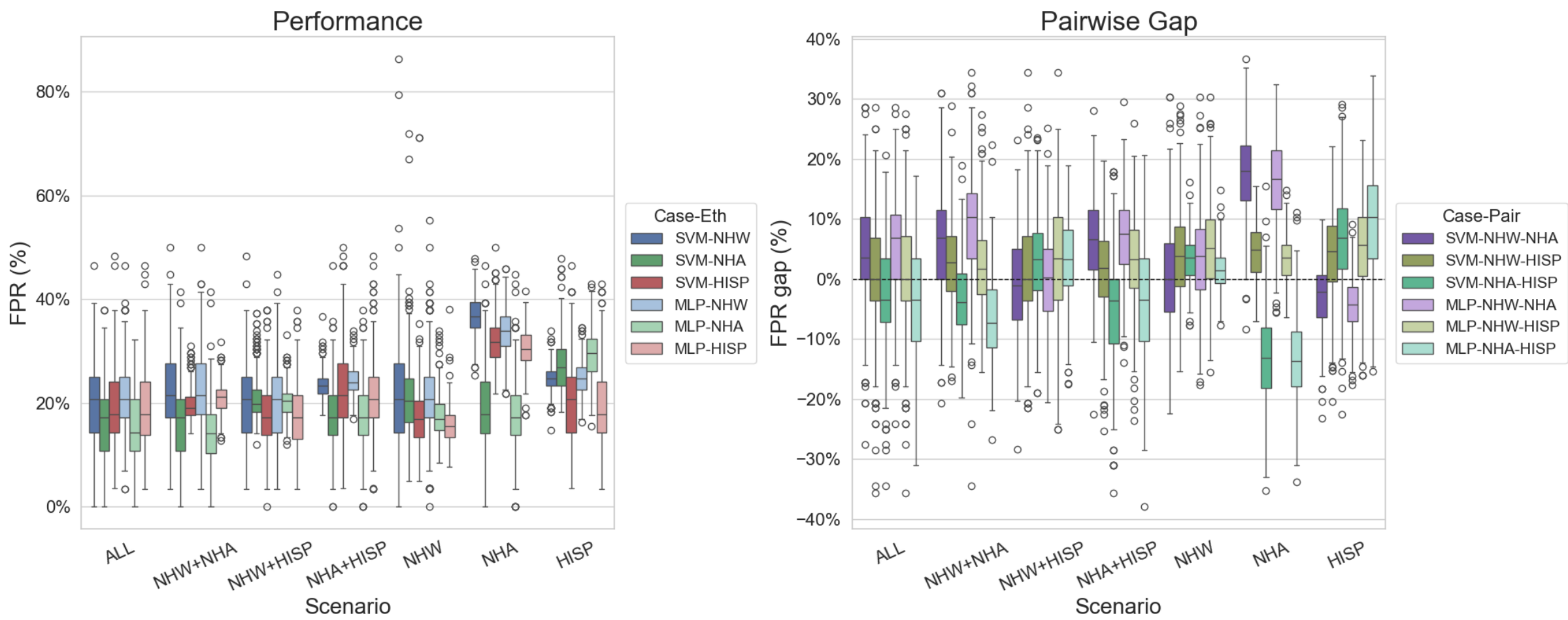

(c) False positive rate (FPR) and FPR gaps.

Supplementary Figure 3: Alternative classifier performance and pairwise gaps across training scenarios. Box plots compare dementia classification performance and pairwise racial/ethnic gaps using support vector machine (SVM) and multi-layer perceptron (MLP) across seven training scenarios. Panels show (a) balanced accuracy (BA), (b) false negative rate (FNR), and (c) false positive rate (FPR); left plots show ethnicity-stratified performance and right plots show pairwise gaps. Box centers indicate medians, bounds indicate the 25th and 75th percentiles, whiskers extend to non-outlier values within 1.5 times the interquartile range, and points indicate outliers. Pairwise gaps are computed as differences between groups for each metric; the horizontal dashed line indicates zero gap. Sample sizes are provided in Supplementary Table 2. NHW: non-Hispanic White; NHA: non-Hispanic African; HISP: Hispanic White. Source data are provided as Source Data 4.

NHW

Pair	Metric	Gap (95% CI)	t-test
NHW–NHA	BA	3.76 ($[2.93,\ 4.64]$)	$p = 1.41e^{-13}$ *
	FNR	-14.65 ($[-15.77,\ -13.54]$)	$p = 3.02e^{-45}$ *
	FPR	7.13 ($[5.75,\ 8.49]$)	$p = 6.81e^{-18}$ *
NHW–HISP	BA	4.16 ($[3.35,\ 4.99]$)	$p = 3.38e^{-16}$ *
	FNR	-14.91 ($[-15.98,\ -13.79]$)	$p = 4.79e^{-46}$ *
	FPR	6.58 ($[5.30,\ 7.78]$)	$p = 5.49e^{-18}$ *
NHA–HISP	BA	0.40 ($[0.01,\ 0.80]$)	$p = 4.85e^{-2}$ *
	FNR	-0.25 ($[-0.86,\ 0.44]$)	$p = 0.44$
	FPR	-0.55 ($[-1.01,\ -0.05]$)	$p = 2.64e^{-2}$ *

NHA

Pair	Metric	Gap (95% CI)	t-test
NHW–NHA	BA	-5.59 ($[-6.45,\ -4.70]$)	$p = 5.10e^{-22}$ *
	FNR	-9.07 ($[-10.60,\ -7.48]$)	$p = 7.90e^{-20}$ *
	FPR	20.26 ($[19.13,\ 21.33]$)	$p = 5.66e^{-58}$ *
NHW–HISP	BA	-0.82 ($[-1.23,\ -0.38]$)	$p = 3.53e^{-4}$ *
	FNR	-6.87 ($[-7.53,\ -6.19]$)	$p = 1.31e^{-36}$ *
	FPR	8.51 ($[7.83,\ 9.14]$)	$p = 1.93e^{-45}$ *
NHA–HISP	BA	4.77 ($[3.86,\ 5.65]$)	$p = 1.54e^{-17}$ *
	FNR	2.20 ($[0.56,\ 3.90]$)	$p = 1.18e^{-2}$ *
	FPR	-11.75 ($[-12.82,\ -10.59]$)	$p = 2.32e^{-36}$ *

HISP

Pair	Metric	Gap (95% CI)	t-test
NHW–NHA	BA	-0.73 ($[-1.16,\ -0.27]$)	$p = 1.97e^{-3}$ *
	FNR	-7.21 ($[-7.79,\ -6.68]$)	$p = 2.78e^{-45}$ *
	FPR	8.67 ($[7.95,\ 9.36]$)	$p = 2.31e^{-43}$ *
NHW–HISP	BA	-4.57 ($[-5.50,\ -3.63]$)	$p = 1.09e^{-15}$ *
	FNR	-1.76 ($[-3.31,\ -0.24]$)	$p = 2.92e^{-2}$ *
	FPR	10.89 ($[9.76,\ 12.06]$)	$p = 2.84e^{-34}$ *
NHA–HISP	BA	-3.84 ($[-4.79,\ -2.92]$)	$p = 3.97e^{-12}$ *
	FNR	5.45 ($[3.99,\ 6.95]$)	$p = 5.07e^{-10}$ *
	FPR	2.22 ($[0.99,\ 3.50]$)	$p = 7.81e^{-4}$ *

ALL

Pair	Metric	Gap (95% CI)	t-test
NHW–NHA	BA	4.24 ($[3.10,\ 5.36]$)	$p = 9.86e^{-11}$ *
	FNR	-16.23 ($[-17.78,\ -14.58]$)	$p = 1.47e^{-35}$ *
	FPR	7.76 ($[6.18,\ 9.31]$)	$p = 1.19e^{-15}$ *
NHW–HISP	BA	5.14 ($[4.12,\ 6.17]$)	$p = 2.96e^{-16}$ *
	FNR	-12.96 ($[-14.85,\ -11.14]$)	$p = 4.19e^{-25}$ *
	FPR	2.67 ($[1.01,\ 4.39]$)	$p = 2.63e^{-3}$ *
NHA–HISP	BA	0.90 ($[-0.24,\ 2.09]$)	$p = 0.14$
	FNR	3.28 ($[0.91,\ 5.45]$)	$p = 5.94e^{-3}$ *
	FPR	-5.09 ($[-6.73,\ -3.49]$)	$p = 2.41e^{-8}$ *

Supplementary Table 3: Statistical tests for pairwise ethnic gaps. Each panel reports the mean pairwise gap with its 95% confidence interval in percentage, formatted as gap $([\mathrm{CI}_{\mathrm{lo}}, \mathrm{CI}_{\mathrm{hi}}])$, and the associated two-sided t-test p-value. * indicates statistical significance at $p < 0.05$. Panel titles denote the training scenario (NHW, NHA, HISP, and ALL).

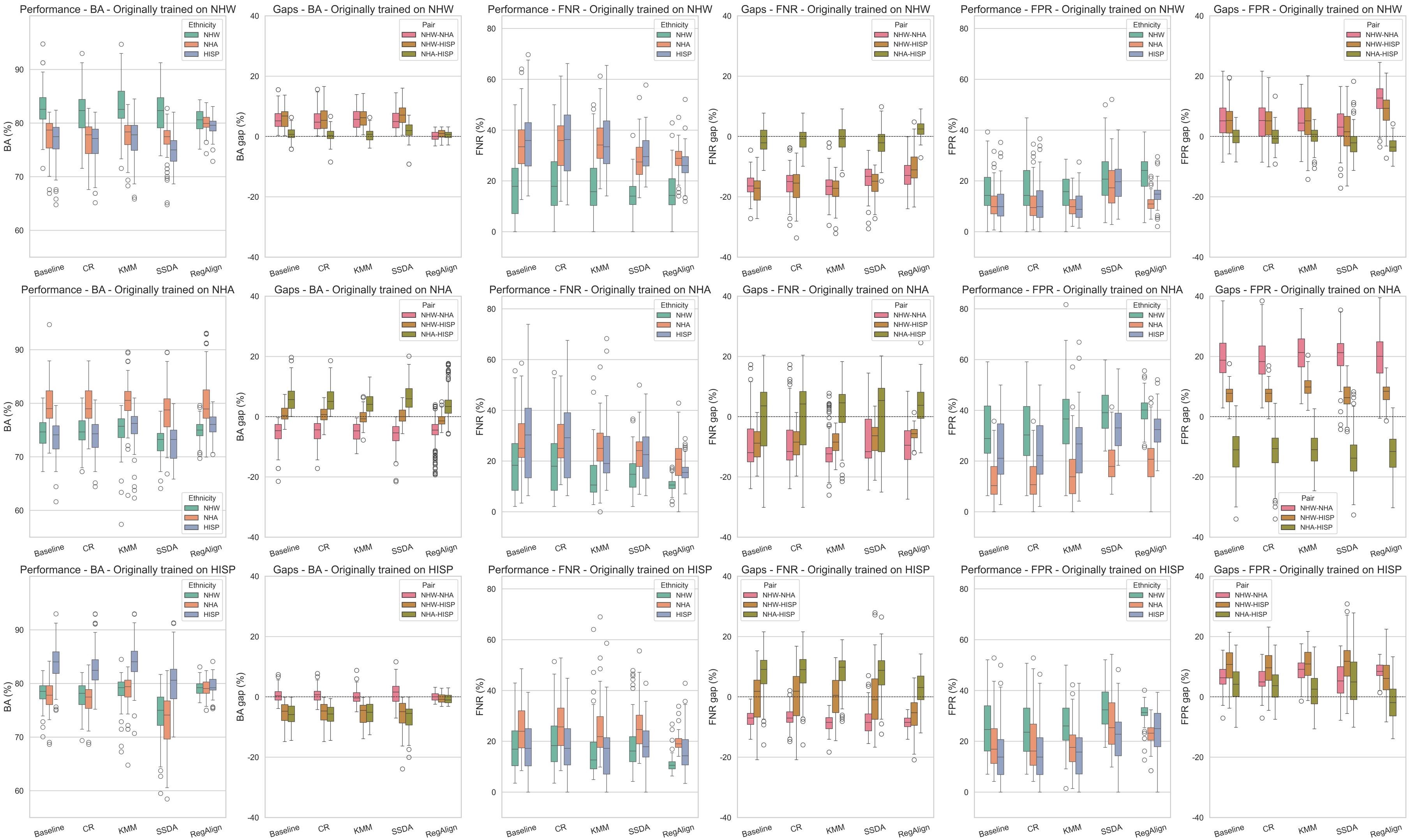

Supplementary Figure 4: Method comparison for discrepancy mitigation under single-group training scenarios. Box plots compare Baseline, CR, KMM, SSDA, and RegAlign for models originally trained on NHW, NHA, or HISP participants. Panels show ethnicity-stratified performance and pairwise racial/ethnic gaps for balanced accuracy (BA), false negative rate (FNR), and false positive rate (FPR). For each metric, performance panels show results within NHW, NHA, and HISP evaluation groups, while gap panels show pairwise differences between groups. Box centers indicate medians, bounds indicate the 25th and 75th percentiles, whiskers extend to non-outlier values within 1.5 times the interquartile range, and points indicate outliers. Sample sizes for each experimental setting are provided in Supplementary Table 2. NHW: non-Hispanic White; NHA: non-Hispanic African; HISP: Hispanic White; CR: correlation remover; KMM: kernel mean matching; SSDA: semi-supervised domain adaptation. Source data are provided as Source Data 2a.

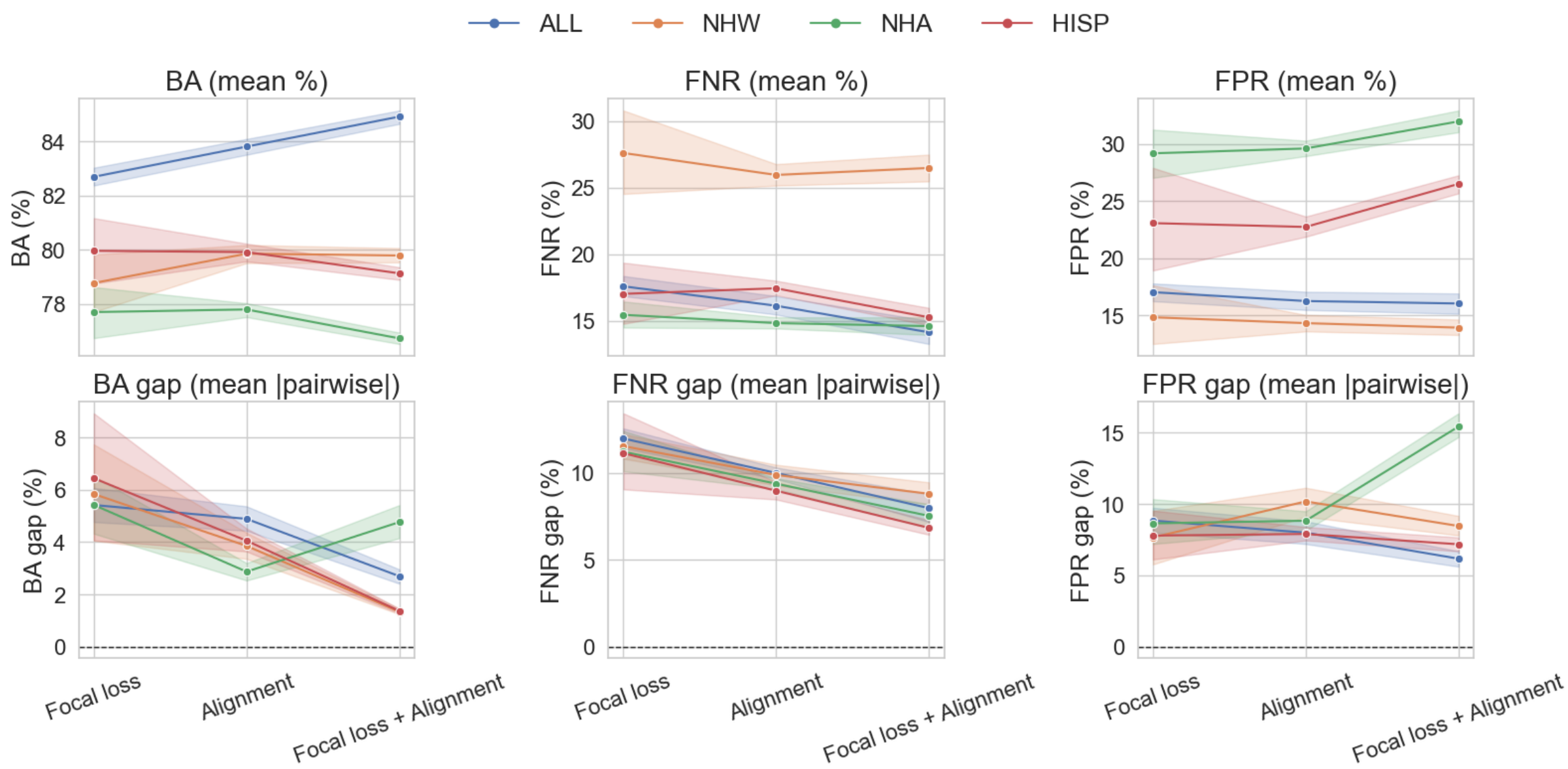

Supplementary Figure 5: Performance and inter-group gaps across RegAlign loss-component ablations. Line plots compare three RegAlign objective variants across four training scenarios: ALL, NHW, NHA, and HISP. The top row shows mean balanced accuracy (BA), false negative rate (FNR), and false positive rate (FPR). The bottom row shows the corresponding mean absolute pairwise gaps, averaged across racial/ethnic group pairs within each scenario. The three objectives all include the source classification loss $\mathcal{L}^S_{\text{cls}}$ and compare **Focal loss** ($\mathcal{L}^S_{\text{cls}} + \mathcal{L}^T_{\text{cls}}$), **Alignment** ($\mathcal{L}^S_{\text{cls}} + \mathcal{L}^{S\leftrightarrow T}_{\text{align}}$), and **Focal loss + Alignment** ($\mathcal{L}^S_{\text{cls}} + \mathcal{L}^T_{\text{cls}} + \mathcal{L}^{S\leftrightarrow T}_{\text{align}}$). Shaded bands indicate 95% confidence intervals across 100 experimental runs. NHW: non-Hispanic White; NHA: non-Hispanic African; HISP: Hispanic White. Source data are provided as Source Data 5.

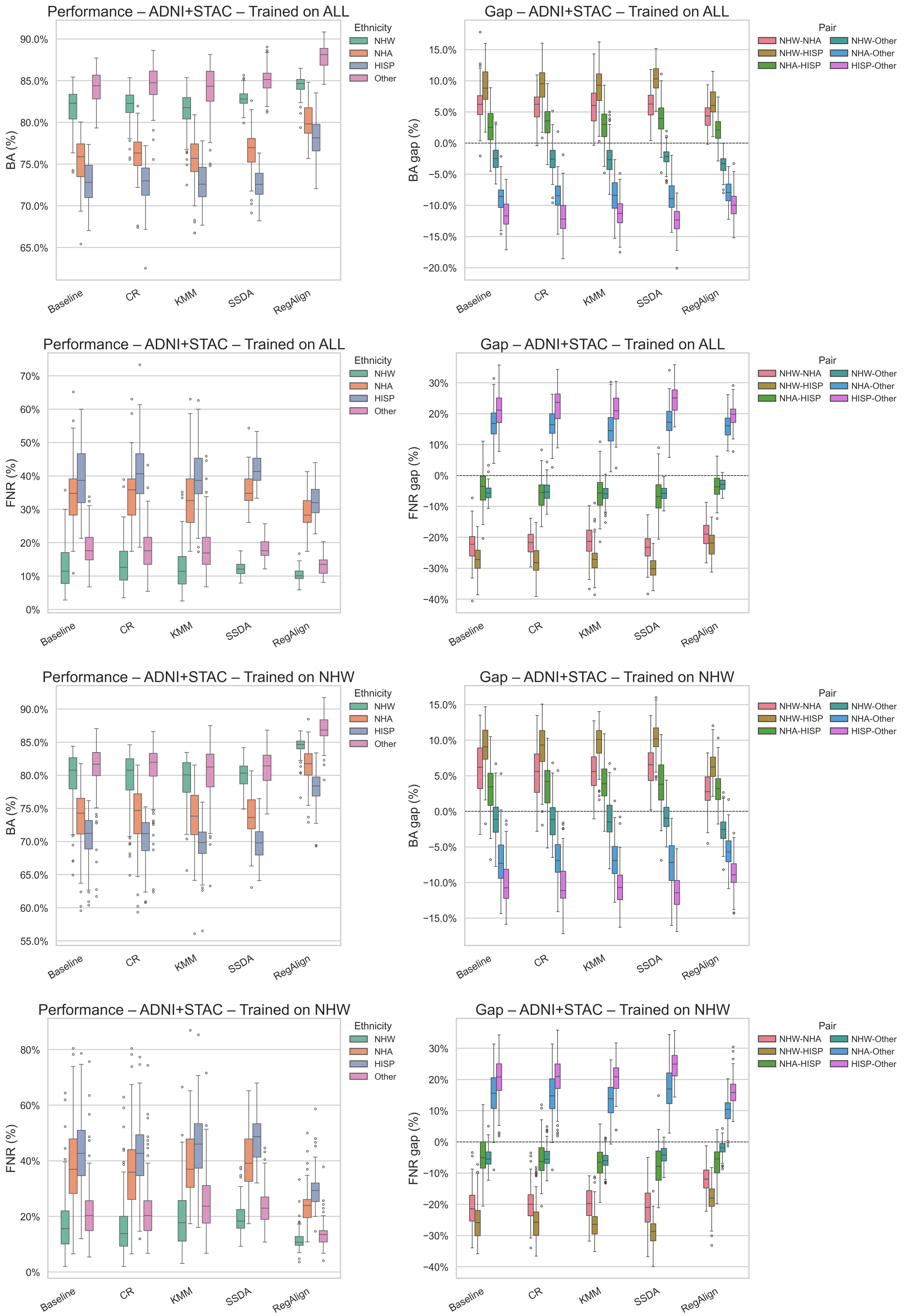

Supplementary Figure 6: External validation on ADNI+STAC for models trained on ALL and NHW. Box plots show BA and FNR performance and pairwise racial/ethnic gaps on ADNI+STAC for models trained on ALL or NHW participants. Left panels show group-stratified performance; right panels show pairwise gaps. Methods include Baseline, CR, KMM, SSDA, and RegAlign. NHW, non-Hispanic White; NHA, non-Hispanic African American; HISP, Hispanic White; BA, balanced accuracy; FNR, false negative rate. Source data are provided as Supplementary Data 6.

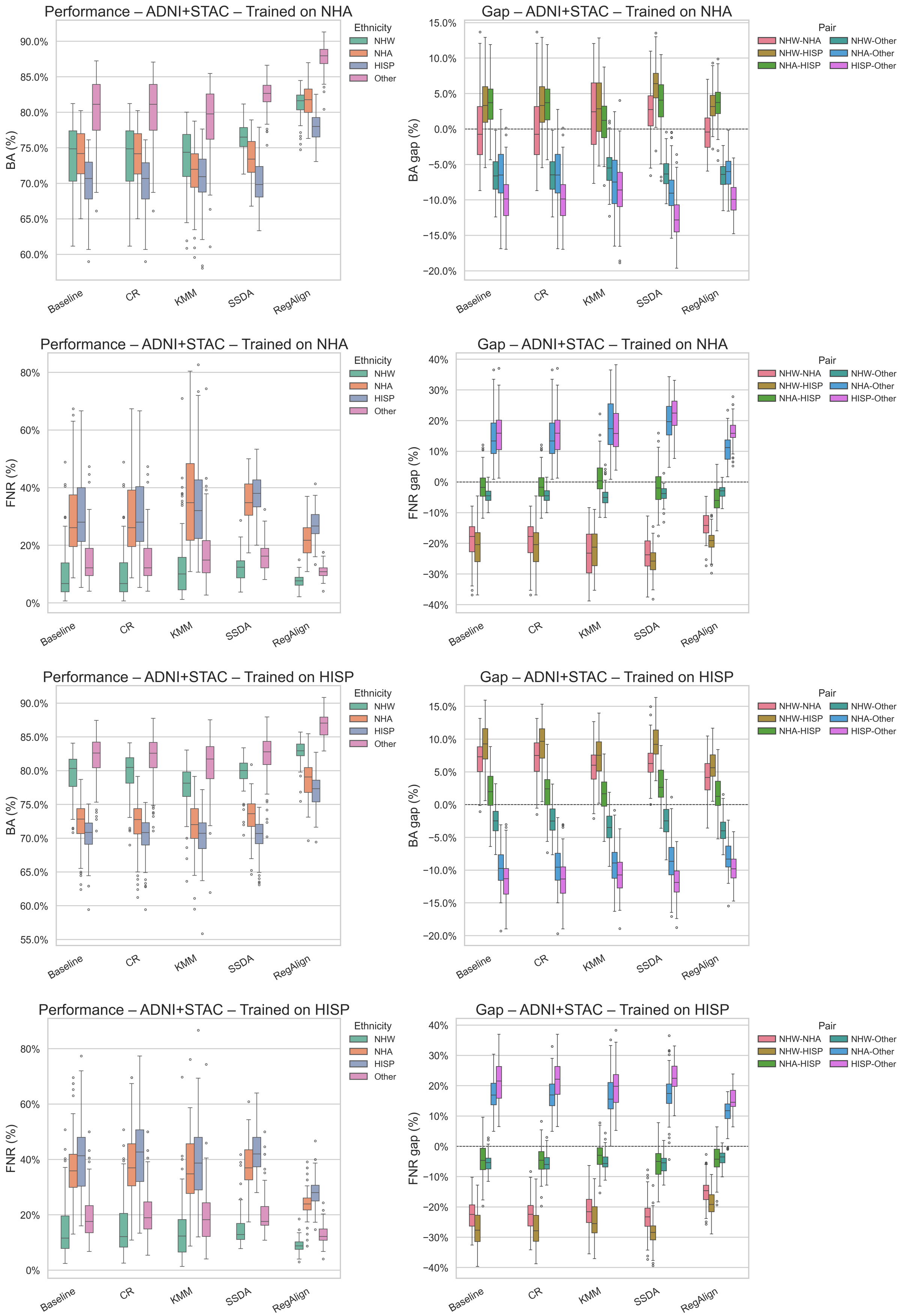

Supplementary Figure 7: External validation on ADNI+STAC for models trained on NHA and HISP. Box plots show BA and FNR performance and pairwise racial/ethnic gaps on ADNI+STAC for models trained on NHA or HISP participants. Left panels show group-stratified performance; right panels show pairwise gaps. Methods include Baseline, CR, KMM, SSDA, and RegAlign. NHW, non-Hispanic White; NHA, non-Hispanic African American; HISP, Hispanic White; BA, balanced accuracy; FNR, false negative rate. Source data are provided as Supplementary Data 6.

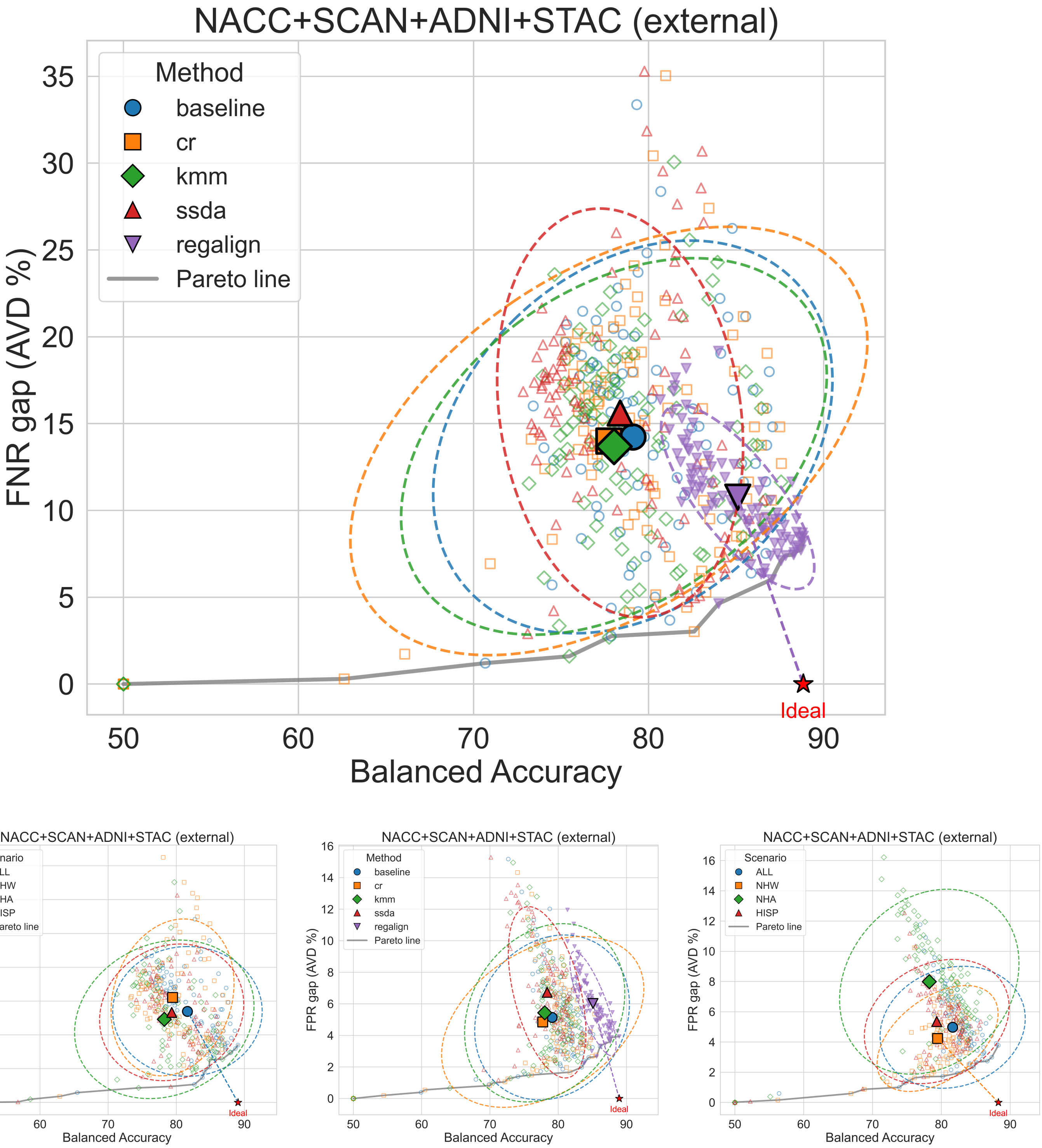

Supplementary Figure 8: Pareto-front analysis of accuracy–fairness trade-offs in external validation. Scatter plots show balanced accuracy (BA; x-axis) versus average discrepancy (AvD; y-axis) in the combined external evaluation set (NACC+SCAN+ADNI+STAC), with lower AvD indicating smaller inter-group discrepancy. The enlarged top panel shows AvD for false negative rate (FNR) across discrepancy mitigation methods. The bottom row shows three complementary analyses: AvD for FNR across training scenarios (left), AvD for false positive rate (FPR) across discrepancy mitigation methods (middle), and AvD for FPR across training scenarios (right). Each point represents a four-run bin, large filled markers indicate centroids, dashed ellipses summarize point distributions, the gray curve marks the Pareto frontier, and the red star denotes the ideal point with high BA and low discrepancy. CR, correlation remover; KMM, kernel mean matching; SSDA, semi-supervised domain adaptation. Source data are provided as Source Data 8.

Supplementary Table 4: Top-K robustness sensitivity analysis across methods and training scenarios. For each method and scenario, configurations were ranked by the selection objective score. We report the top-K mean with bootstrap CI (2000 resamples), the mean of the remaining configurations with CI, and $\Delta = \mu_{\text{top-}K} - \mu_{\text{rest}}$. Values are reported without percentage symbols. Scenarios are ordered as ALL, NHW, NHA, and HISP.

Method	Scenario	n_{total}	K	Top-K mean (CI)	Rest mean (CI)	Δ
Baseline	ALL	400	80	81.198 (81.183–81.214)	80.443 (80.393–80.492)	0.755
	NHW	400	80	78.330 (78.296–78.363)	77.531 (77.472–77.586)	0.799
	NHA	400	80	78.227 (78.199–78.256)	77.551 (77.502–77.595)	0.676
	HISP	400	80	79.108 (79.076–79.141)	77.966 (77.897–78.029)	1.142
CR	ALL	100	20	80.744 (80.727–80.762)	80.573 (80.555–80.590)	0.171
	NHW	100	20	78.804 (78.795–78.815)	78.760 (78.754–78.764)	0.045
	NHA	100	20	78.716 (78.716–78.716)	78.706 (78.704–78.707)	0.010
	HISP	100	20	79.190 (79.183–79.197)	79.143 (79.139–79.146)	0.047
KMM	ALL	108	22	79.439 (79.399–79.485)	78.986 (78.936–79.037)	0.453
	NHW	108	22	77.277 (77.232–77.326)	76.773 (76.721–76.826)	0.504
	NHA	108	22	74.320 (74.212–74.441)	73.346 (73.244–73.440)	0.974
	HISP	108	22	76.921 (76.843–77.003)	76.277 (76.216–76.337)	0.643
SSDA	ALL	540	108	80.868 (80.798–80.949)	80.193 (80.132–80.255)	0.675
	NHW	540	108	77.226 (77.226–77.226)	76.051 (75.972–76.134)	1.175
	NHA	540	108	74.265 (74.118–74.412)	73.386 (73.334–73.436)	0.879
	HISP	540	108	77.075 (76.890–77.255)	76.147 (76.091–76.198)	0.927
RegAlign	ALL	540	108	80.315 (80.312–80.318)	80.155 (80.149–80.160)	0.160
	NHW	540	108	78.474 (78.474–78.474)	78.222 (78.212–78.232)	0.252
	NHA	540	108	78.483 (78.483–78.483)	78.355 (78.346–78.363)	0.128
	HISP	540	108	79.013 (79.013–79.013)	78.824 (78.819–78.830)	0.189

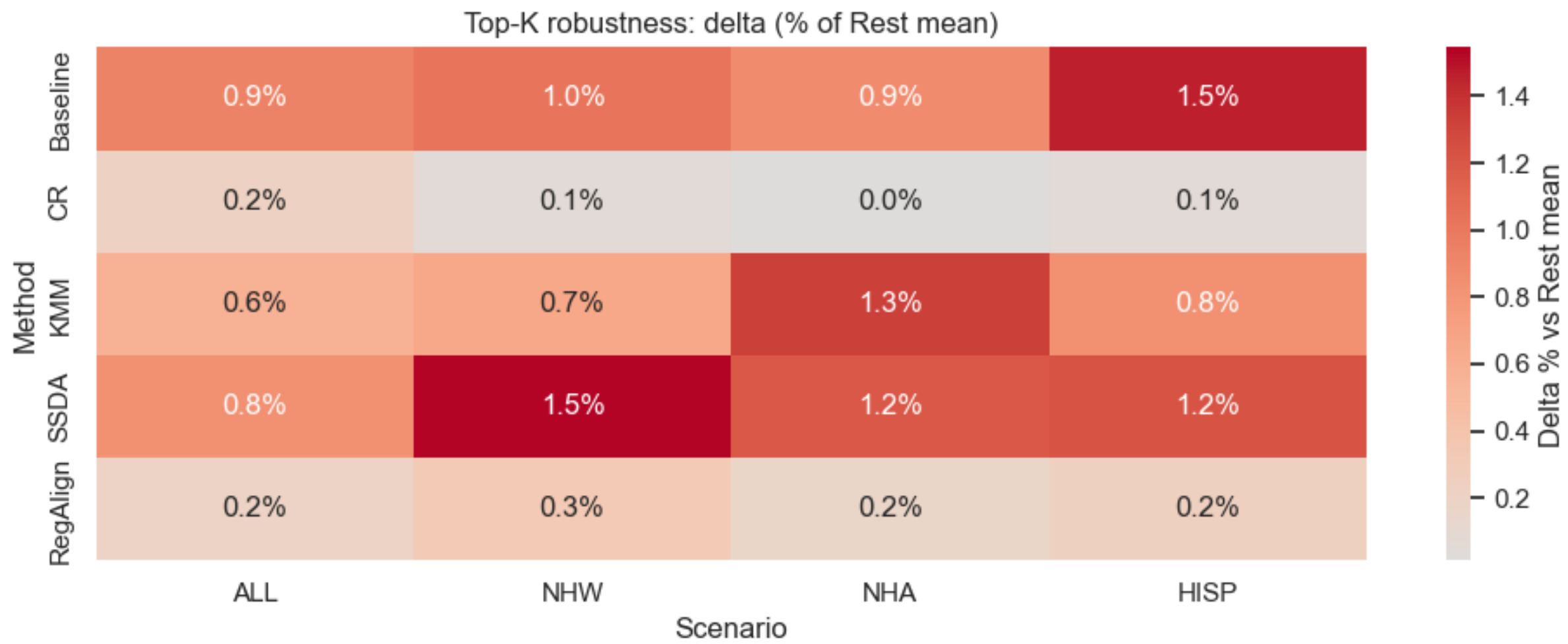

Supplementary Figure 9: Top-K robustness sensitivity (normalized). Heatmap of $\Delta\% = \frac{\mu_{\text{top-}K} - \mu_{\text{rest}}}{\mu_{\text{rest}}} \times 100\%$ for each method and training scenario. Larger values indicate greater separation between the top-ranked configurations and the remaining configurations. Source data are provided as a Source Data 6.

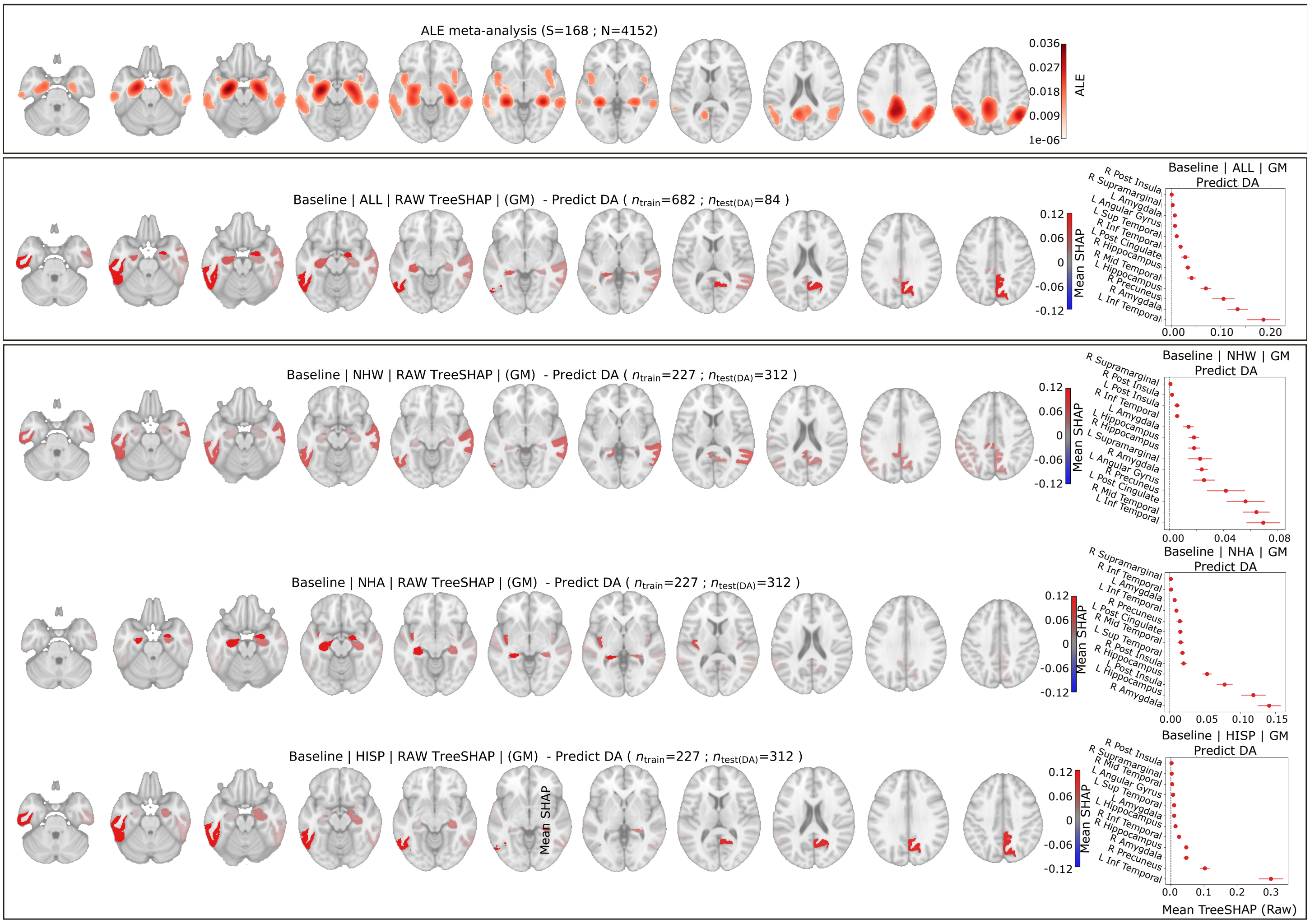

Supplementary Figure 10: ALE-guided SHAP brain-map visualization across training scenarios. Top row: ALE meta-analysis reference pattern for dementia-related structural alterations. Rows $2nd$–$5th$: TreeSHAP-based attribution maps (GM features) for baseline models trained on ALL, NHW, NHA, and HISP, respectively. For each scenario, left panels show the spatial projection of SHAP contributions on representative axial slices, and the right panel summarizes the mean SHAP (with 95% CI) for the most influential ALE-guided regions when predicting dementia. Differences in attribution ranking and concentration across scenarios highlight cohort-dependent decision patterns.

(a) Trained on ALL

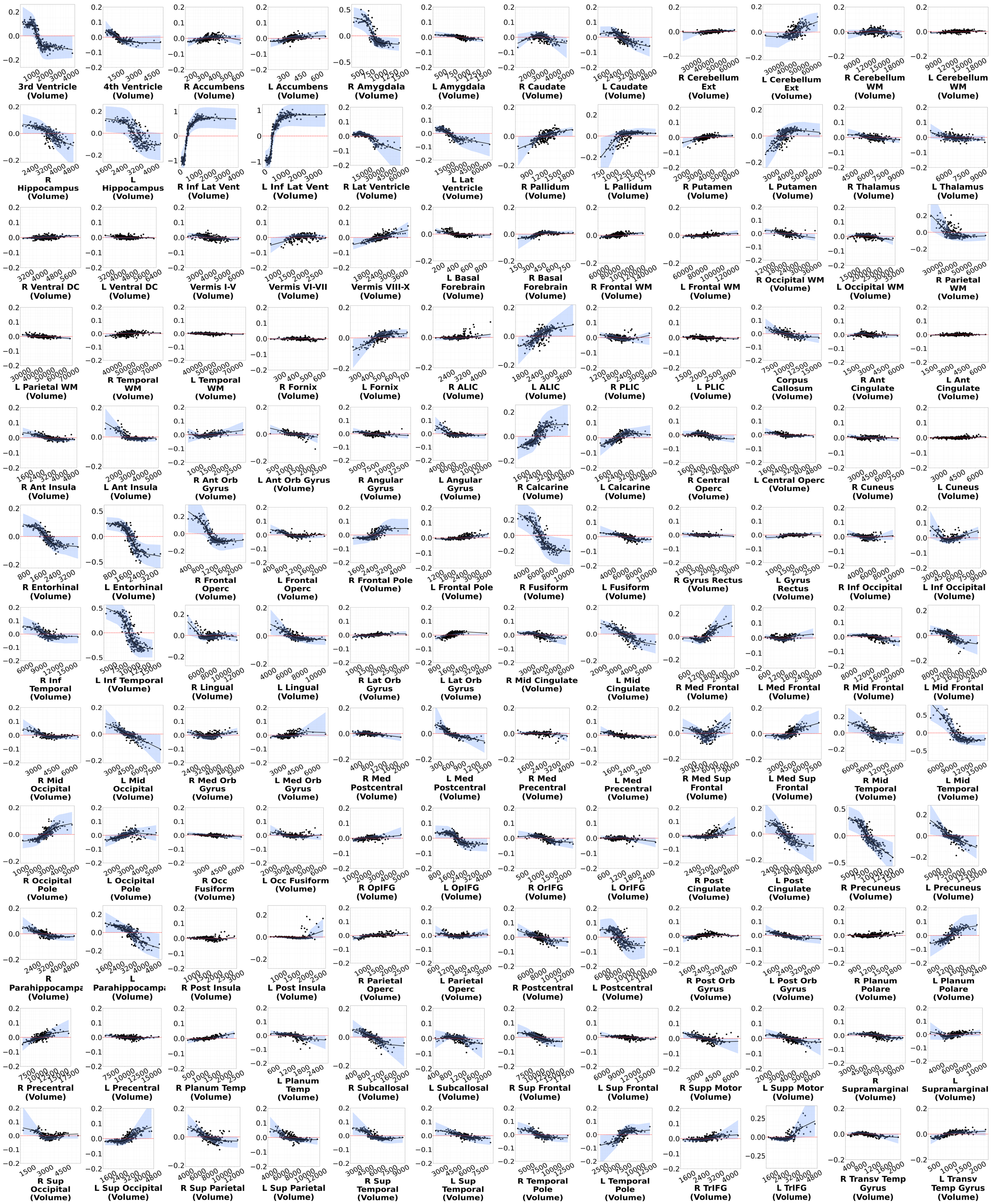

(b) Trained on NHW

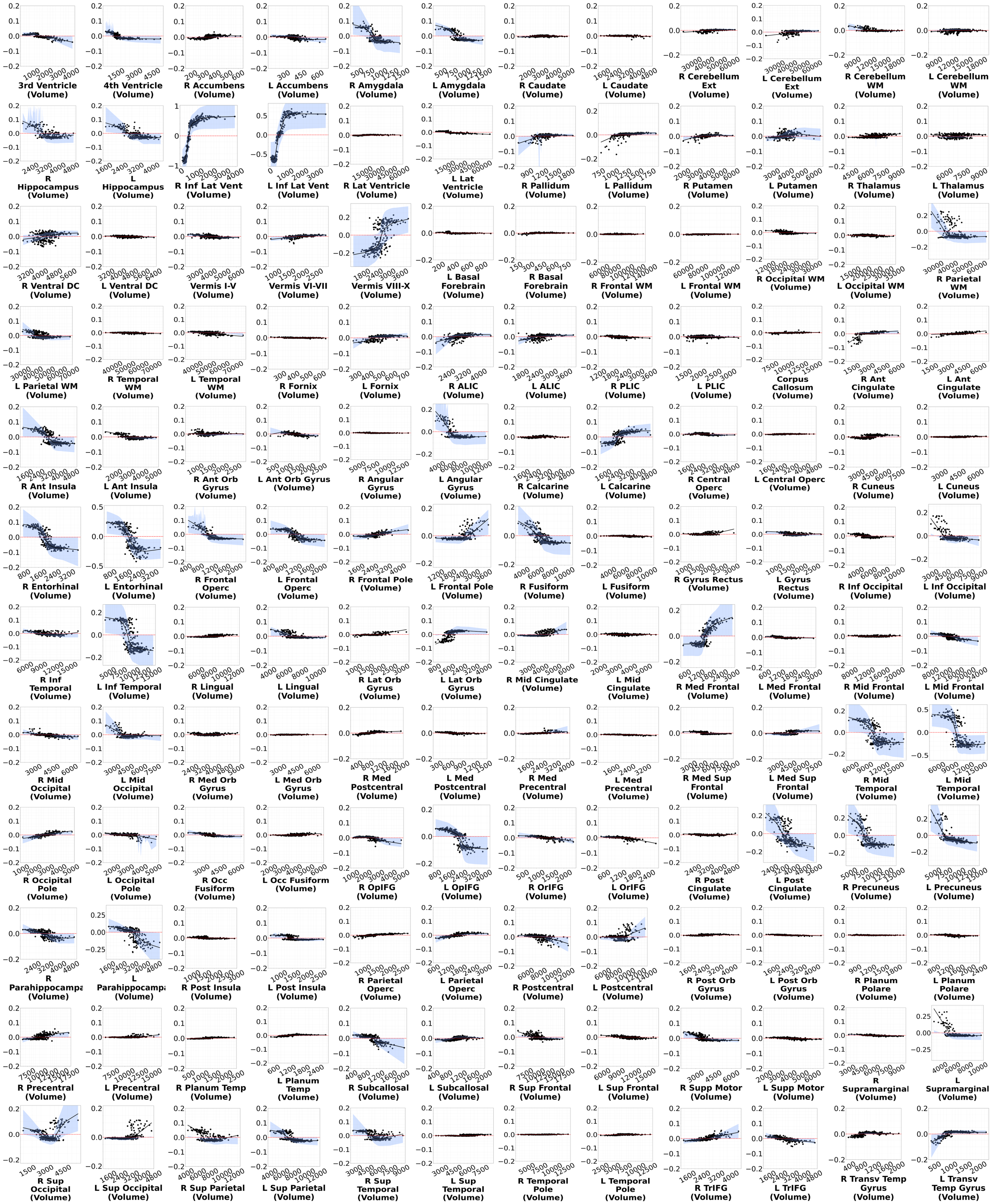

(c) Trained on NHA

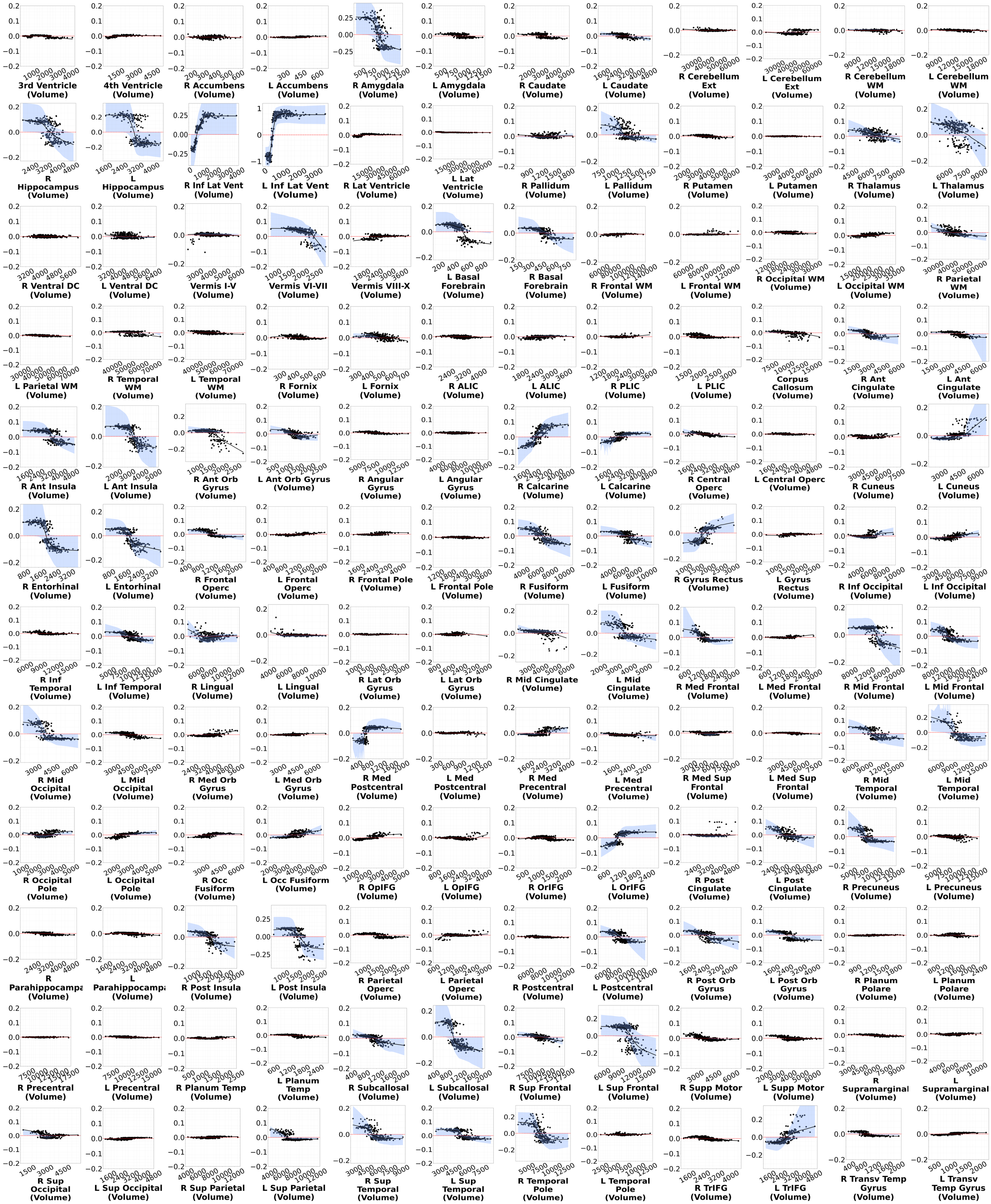

(d) Trained on HISP

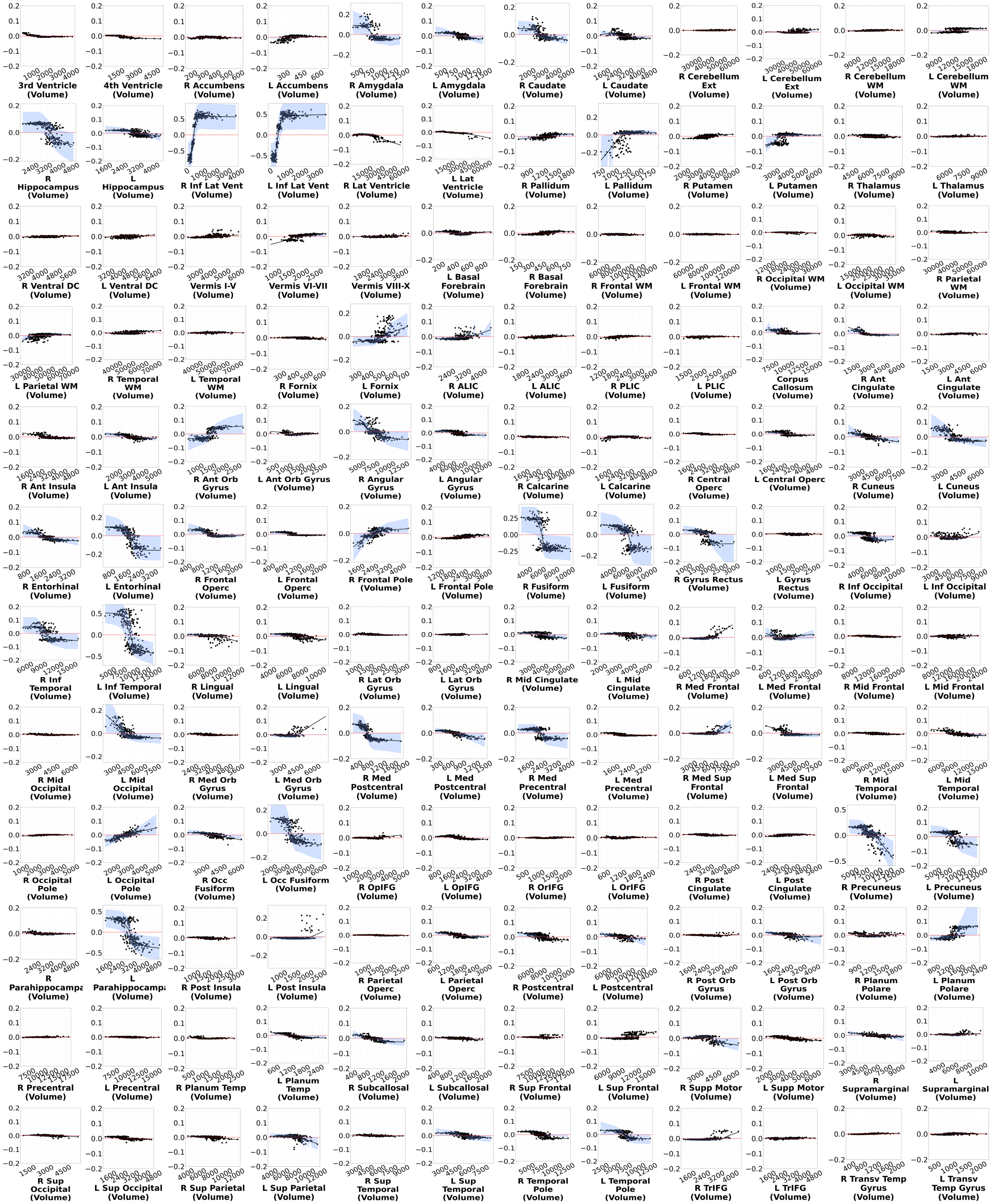

(e) Dictionary of 144 brain regions

3rd Ventricle-VN	4th Ventricle-VN	R Accumbens-GM	L Accumbens-GM	R Amygdala-GM	L Amygdala-GM	R Caudate-GM	L Caudate-GM	R Cerebellum Ext-GM	L Cerebellum Ext-GM	R Cerebellum WM-WM	L Cerebellum WM-WM
R Hippocampus-GM	L Hippocampus-GM	R Inf Lat Vent-VN	L Inf Lat Vent-VN	R Lat Ventricle-VN	L Lat Ventricle-VN	R Pallidum-GM	L Pallidum-GM	R Putamen-GM	L Putamen-GM	R Thalamus-GM	L Thalamus-GM
R Ventral DC-WM	L Ventral DC-WM	Vermis I-V-GM	Vermis VI-VII-GM	Vermis VIII-X-GM	L Basal Forebrain-GM	R Basal Forebrain-GM	R Frontal WM-WM	L Frontal WM-WM	R Occipital WM-WM	L Occipital WM-WM	R Parietal WM-WM
L Parietal WM-WM	R Temporal WM-WM	L Temporal WM-WM	R Fornix-WM	L Fornix-WM	R ALIC-WM	L ALIC-WM	R PLIC-WM	L PLIC-WM	Corpus Callosum-WM	R Ant Cingulate-GM	L Ant Cingulate-GM
R Ant Insula-GM	L Ant Insula-GM	R Ant Orb Gyrus-GM	L Ant Orb Gyrus-GM	R Angular Gyrus-GM	L Angular Gyrus-GM	R Calcarine-GM	L Calcarine-GM	R Central Operc-GM	L Central Operc-GM	R Cuneus-GM	L Cuneus-GM
R Entorhinal-GM	L Entorhinal-GM	R Frontal Operc-GM	L Frontal Operc-GM	R Frontal Pole-GM	L Frontal Pole-GM	R Fusiform-GM	L Fusiform-GM	R Gyrus Rectus-GM	L Gyrus Rectus-GM	R Inf Occipital-GM	L Inf Occipital-GM
R Inf Temporal-GM	L Inf Temporal-GM	R Lingual-GM	L Lingual-GM	R Lat Orb Gyrus-GM	L Lat Orb Gyrus-GM	R Mid Cingulate-GM	L Mid Cingulate-GM	R Med Frontal-GM	L Med Frontal-GM	R Mid Frontal-GM	L Mid Frontal-GM
R Mid Occipital-GM	L Mid Occipital-GM	R Med Orb Gyrus-GM	L Med Orb Gyrus-GM	R Med Postcentral-GM	L Med Postcentral-GM	R Med Precentral-GM	L Med Precentral-GM	R Med Sup Frontal-GM	L Med Sup Frontal-GM	R Mid Temporal-GM	L Mid Temporal-GM
R Occipital Pole-GM	L Occipital Pole-GM	R Occ Fusiform-GM	L Occ Fusiform-GM	R OpIFG-GM	L OpIFG-GM	R OrIFG-GM	L OrIFG-GM	R Post Cingulate-GM	L Post Cingulate-GM	R Precuneus-GM	L Precuneus-GM
R Parahippocampal-GM	L Parahippocampal-GM	R Post Insula-GM	L Post Insula-GM	R Parietal Operc-GM	L Parietal Operc-GM	R Postcentral-GM	L Postcentral-GM	R Post Orb Gyrus-GM	L Post Orb Gyrus-GM	R Planum Polare-GM	L Planum Polare-GM
R Precentral-GM	L Precentral-GM	R Planum Temp-GM	L Planum Temp-GM	R Subcallosal-GM	L Subcallosal-GM	R Sup Frontal-GM	L Sup Frontal-GM	R Supp Motor-GM	L Supp Motor-GM	R Supramarginal-GM	L Supramarginal-GM
R Sup Occipital-GM	L Sup Occipital-GM	R Sup Parietal-GM	L Sup Parietal-GM	R Sup Temporal-GM	L Sup Temporal-GM	R Temporal Pole-GM	L Temporal Pole-GM	R TrIFG-GM	L TrIFG-GM	R Transv Temp Gyrus-GM	L Transv Temp Gyrus-GM

Supplementary Figure 11: Partial dependence plots for all 144 brain regions across training scenarios. Partial dependence plots show the relationship between regional brain volume and SHAP contribution for dementia classification. Panels show models trained on **(a)** ALL, **(b)** NHW, **(c)** NHA, and **(d)** HISP. Panel **(e)** provides the definitional table of the 144 MUSE-derived brain regions, ordered from left to right and top to bottom as shown in panels **(a–d)**. Positive SHAP values indicate contribution toward dementia prediction, while negative values support normal cognition classification. NHW, non-Hispanic White; NHA, non-Hispanic African American; HISP, Hispanic White. Source data are provided as Source Data 7.

References

[1] Adkins, J., Bowling, M., and White, A. (2024). A method for evaluating hyperparameter sensitivity in reinforcement learning. *Advances in Neural Information Processing Systems*, 37:124820–124842.
