# Supplementary material for: Advancing fair and explainable machine learning for neuroimaging dementia pattern classification in multi-racial and multi-ethnic populations
Source: Nat Commun. 2026 Jun 26;17:8026. doi: 10.1038/s41467-026-74515-w (PMC13454470; doi:10.1038/s41467-026-74515-w)
Supplement: Supplementary file 3 [file 41467_2026_74515_MOESM2_ESM.pdf]

Description of Additional Supplementary Files

File name: Supplementary Data 1

Description: Supplementary Data 1 is complete comparison on performance between baseline and bias mitigation methods across three racial-ethnic groups, non-Hispanic White (NHW), non-Hispanic African (NHA), and Hispanic (HISP), in term of Balanced Accuracy (BA), False Positive Rate (FPR), and False Negative Rate (FNR).

File name: Supplementary Data 2

Description: Supplementary Data 2 is complete comparison on racial/ethnic pair-wise gaps between baseline and bias mitigation methods across three racial-ethnic groups, non-Hispanic White (NHW), non-Hispanic African (NHA), and Hispanic (HISP), in term of Balanced Accuracy (BA), False Positive Rate (FPR), and False Negative Rate (FNR).

File name: Supplementary Data 3

Description: Supplementary Data 3 is two-sided t-test for performance comparison of baseline versus bias mitigation methods over 100-run replicates.

File name: Supplementary Data 4

Description: Supplementary Data 4 is two-sided t-test for pai-wise racial/ethnic gap comparison of baseline versus bias mitigation methods over 100-run replicates.

File name: Supplementary Data 5

Description: Supplementary Data 5 is comparison on performance and gap in external test set NACC+SCAN. Two-sided t-test was applied to compute statical difference between bias mitigation methods and baseline. No adjustment was applied for multiple comparisons.

File name: Supplementary Data 6

Description: Supplementary Data 6 is comparison on performance and gap in external test set ADNI+STAC. Two-sided t-test was applied to compute statical difference between bias mitigation methods and baseline. No adjustment was applied for multiple comparisons.
