# Supplementary material for: Advancing fair and explainable machine learning for neuroimaging dementia pattern classification in multi-racial and multi-ethnic populations
Source: Nat Commun. 2026 Jun 26;17:8026. doi: 10.1038/s41467-026-74515-w (PMC13454470; doi:10.1038/s41467-026-74515-w)
Supplement: Supplementary file 4 [file 41467_2026_74515_MOESM9_ESM.pdf]

nature portfolio

Corresponding author(s): Mohamad Habes

Last updated by author(s): Apr 29, 2026

Reporting Summary

Nature Portfolio wishes to improve the reproducibility of the work that we publish. This form provides structure for consistency and transparency in reporting. For further information on Nature Portfolio policies, see our Editorial Policies and the Editorial Policy Checklist.

Statistics

For all statistical analyses, confirm that the following items are present in the figure legend, table legend, main text, or Methods section.

n/a	Confirmed	
☐	☒	The exact sample size (*n*) for each experimental group/condition, given as a discrete number and unit of measurement
☒	☐	A statement on whether measurements were taken from distinct samples or whether the same sample was measured repeatedly
☐	☒	The statistical test(s) used AND whether they are one- or two-sided<br>*Only common tests should be described solely by name; describe more complex techniques in the Methods section.*
☐	☒	A description of all covariates tested
☒	☐	A description of any assumptions or corrections, such as tests of normality and adjustment for multiple comparisons
☐	☒	A full description of the statistical parameters including central tendency (e.g. means) or other basic estimates (e.g. regression coefficient) AND variation (e.g. standard deviation) or associated estimates of uncertainty (e.g. confidence intervals)
☐	☒	For null hypothesis testing, the test statistic (e.g. *F*, *t*, *r*) with confidence intervals, effect sizes, degrees of freedom and *P* value noted<br>*Give P values as exact values whenever suitable.*
☒	☐	For Bayesian analysis, information on the choice of priors and Markov chain Monte Carlo settings
☒	☐	For hierarchical and complex designs, identification of the appropriate level for tests and full reporting of outcomes
☒	☐	Estimates of effect sizes (e.g. Cohen's *d*, Pearson's *r*), indicating how they were calculated

Our web collection on statistics for biologists contains articles on many of the points above.

Software and code

Policy information about availability of computer code

Data collection	- NACC clinical and related research data are available to qualified researchers worldwide through https://www.naccdata.org/data-request-process<br>- SCAN images are available through NACC's data request system https://scan.naccdata.org/<br>- ADNI data are available to qualified investigators through the Laboratory of Neuro Imaging (LONI) Image and Data Archive (IDA) after acceptance of the ADNI Data Use Agreement at https://adni.loni.usc.edu/data-samples/adni-data/<br>- STAC data is requested through its Population Neuroscience request process at https://stxadrc.org/for-scientists/
Data analysis	Python 3.11, numpy 1.26.4, pandas 2.3.1, scikit-learn 1.7.1, seaborn 0.13.2, matplotlib 3.10.0, xgboost 3.0.0, nibabel 5.3.2, nilearn 0.13.1

For manuscripts utilizing custom algorithms or software that are central to the research but not yet described in published literature, software must be made available to editors and reviewers. We strongly encourage code deposition in a community repository (e.g. GitHub). See the Nature Portfolio guidelines for submitting code & software for further information.

Data

Policy information about availability of data

All manuscripts must include a data availability statement. This statement should provide the following information, where applicable:

- Accession codes, unique identifiers, or web links for publicly available datasets
- A description of any restrictions on data availability
- For clinical datasets or third party data, please ensure that the statement adheres to our policy

The minimum dataset used in this study was obtained from four third-party clinical research resources: the National Alzheimer's Coordinating Center (NACC), the Standardized Centralized Alzheimer's & Related Dementias Neuroimaging initiative (SCAN), the Alzheimer's Disease Neuroimaging Initiative (ADNI), and the South Texas Alzheimer's Disease Research Center (STAC). Because these are human-participant datasets, access is restricted to protect participant privacy and is governed by each resource's data-use procedures and agreements.

NACC clinical and related research data are available to qualified researchers worldwide through the NACC Data Request Process (https://www.naccdata.org/data-request-process). Investigators must submit a data request describing the proposed project and accept the NACC Data Use Agreement. It will acknowledge requests within three business days and may request clarification if needed. Approved datasets are provided through secure download mechanisms maintained by NACC.

SCAN harmonized neuroimaging-derived data, including summary, quality-control, and analysis variables, and defaced SCAN images are available through NACC's data request system (https://scan.naccdata.org/; see also NACC Data Request Process at https://www.naccdata.org/data-request-process). Access is restricted because the resource contains human neuroimaging data and is subject to de-identification, quality-control, and data-use requirements. All submitted image data may be immediately available because additional processing steps, including defacing and QC, may be required.

ADNI data are available to qualified investigators through the Laboratory of Neuro Imaging (LONI) Image and Data Archive (IDA) after acceptance of the ADNI Data Use Agreement and submission of an online application describing the investigator's institutional affiliation and proposed data use (https://adni.loni.usc.edu/data-samples/adni-data/). Applications are generally reviewed within approximately two weeks. Approved users receive login credentials to access and download the requested data through IDA.

To access STAC data, investigators and research groups may request patients, control subjects, biospecimens, and data, including through its Population Neuroscience request process (https://stxadrc.org/for-scientists/). Access is therefore restricted and managed directly by STAC. Researchers seeking access should submit a request through the STAC scientist portal.

Research involving human participants, their data, or biological material

Policy information about studies with human participants or human data. See also policy information about sex, gender (identity/presentation), and sexual orientation and race, ethnicity and racism.

Reporting on sex and gender	This study is a secondary analysis of de-identified, publicly available datasets. Sex information (e.g., female/male) was used only as reported by the originating cohorts and was not collected or re-assessed by the authors; gender identity data were not available in the datasets used. Because the primary objective of this work is to evaluate performance discrepancies across racial/ethnic populations, we did not plan sex- or gender-based analyses a priori and therefore did not perform sex- or gender-stratified outcome analyses.
Reporting on race, ethnicity, or other socially relevant groupings	This study is a secondary analysis of de-identified, publicly available datasets. We used race/ethnicity categories as defined and provided by the originating cohorts to evaluate whether dementia prediction models exhibit systematic performance discrepancies across socially relevant groups. Race/ethnicity was treated as a social categorization variable (not a proxy for socioeconomic status or other constructs) and was not assigned or reclassified by the authors; it was recorded by the parent studies according to their standard procedures (e.g., participant self-report and/or study administrative records as documented by each cohort). Analyses focused on the major U.S. categories available with sufficient sample size (NonHispanic White, Non-Hispanic Black/African American, and Hispanic).
Population characteristics	This study is a secondary analysis of de-identified, publicly available Alzheimer's disease research cohorts. Participants were older adults spanning the cognitive spectrum from cognitively normal (CN) to dementia/Alzheimer's disease–related dementia (DA/ADRD), as defined by the originating cohorts. Covariate-relevant characteristics considered in this study include age, sex (as reported by the parent cohorts), and race/ethnicity (Non-Hispanic White, Non-Hispanic Black/African American, Hispanic, and an "Other" category used for validation). Cohort composition, diagnostic group counts, and key demographics are summarized in Table 1, and we additionally constructed a strictly group-balanced subset with matching by diagnosis, age, and sex to enable fair cross-ethnic comparisons.
Recruitment	This study is a secondary analysis of de-identified, publicly available datasets. No recruitment was performed.
Ethics oversight	This study is a secondary analysis of de-identified data obtained from publicly available research cohorts (NACC,SCAN, ADNI, and STAC). All participant recruitment, informed consent procedures, and ethical approvals were obtained by the originating studies and their respective institutional review boards. The present analysis used only de-identified data and therefore did not require additional ethics approval by the authors' institution.

Note that full information on the approval of the study protocol must also be provided in the manuscript.

Field-specific reporting

Please select the one below that is the best fit for your research. If you are not sure, read the appropriate sections before making your selection.

[x] Life sciences [ ] Behavioural & social sciences [ ] Ecological, evolutionary & environmental sciences

For a reference copy of the document with all sections, see nature.com/documents/nr-reporting-summary-flat.pdf

Life sciences study design

All studies must disclose on these points even when the disclosure is negative.

Sample size

This is a secondary analysis of de-identified, publicly available cohorts; sample size was determined by data availability after applying consistent inclusion/exclusion and quality-control criteria. No prospective power calculation was performed. The final dataset included 9,007 participants, and a strictly group-balanced subset was additionally constructed for controlled cross-ethnic comparisons.

Data exclusions

Exclusions followed pre-specified criteria applied uniformly across cohorts: missing required variables (diagnosis, race/ethnicity, age/sex) and/or failed preprocessing/quality-control for neuroimaging-derived features (e.g., missing/invalid ROI values). Final counts after filtering are reported in Table 1 and the Methods.

Replication

The findings were evaluated using repeated Monte Carlo cross-validation with 100 independent runs and inner 10-fold cross-validation for hyperparameter tuning. Results were assessed across multiple training scenarios, including training on all groups and training on individual population groups, and were further evaluated on independent external datasets. The main findings, including persistent inter-group performance discrepancies and improvement with RegAlign, were reproducible across repeated runs and validation settings.

Randomization

This was a retrospective secondary analysis of de-identified cohort data, so participants were not prospectively randomized into experimental groups. For model evaluation, samples were randomly split into training/validation and test sets using stratified sampling by diagnosis and race/ethnicity where applicable. In the group-balanced analysis, participants were matched by diagnosis, age, and sex across NHW, NHA, and HISP groups to reduce confounding from sample imbalance and demographic differences. External validation datasets were kept separate from model training.

Blinding

Blinding was not applicable because this study used retrospective de-identified data and computational model training rather than prospective intervention or experimental treatment assignment. Data collection was completed by the original cohort studies before the present analysis. Race/ethnicity and diagnosis labels were required for stratified splitting, subgroup performance evaluation, and fairness analysis; therefore, investigators could not be blinded to these variables during analysis.

Reporting for specific materials, systems and methods

We require information from authors about some types of materials, experimental systems and methods used in many studies. Here, indicate whether each material, system or method listed is relevant to your study. If you are not sure if a list item applies to your research, read the appropriate section before selecting a response.

Materials & experimental systems

n/a	Involved in the study
[ ]	[ ] Antibodies
[ ]	[ ] Eukaryotic cell lines
[ ]	[ ] Palaeontology and archaeology
[ ]	[ ] Animals and other organisms
[ ]	[ ] Clinical data
[ ]	[ ] Dual use research of concern
[ ]	[ ] Plants

Methods

n/a	Involved in the study
[ ]	[ ] ChIP-seq
[ ]	[ ] Flow cytometry
[ ]	[x] MRI-based neuroimaging

Antibodies

Antibodies used: Not applicable

Validation: Not applicable

Eukaryotic cell lines

Policy information about cell lines and Sex and Gender in Research

Cell line source(s): Not applicable

Authentication: Not applicable

Mycoplasma contamination	Not applicable
Commonly misidentified lines (See ICLAC register)	Not applicable

Palaeontology and Archaeology

Specimen provenance	Not applicable
Specimen deposition	Not applicable
Dating methods	Not applicable

☐ Tick this box to confirm that the raw and calibrated dates are available in the paper or in Supplementary Information.

Ethics oversight	Not applicable

Note that full information on the approval of the study protocol must also be provided in the manuscript.

Animals and other research organisms

Policy information about studies involving animals; ARRIVE guidelines recommended for reporting animal research, and Sex and Gender in Research

Laboratory animals	Not applicable
Wild animals	Not applicable
Reporting on sex	Not applicable
Field-collected samples	Not applicable
Ethics oversight	Not applicable

Note that full information on the approval of the study protocol must also be provided in the manuscript.

Clinical data

Policy information about clinical studies

All manuscripts should comply with the ICMJE guidelines for publication of clinical research and a completed CONSORT checklist must be included with all submissions.

Clinical trial registration	Not applicable
Study protocol	Not applicable
Data collection	Not applicable
Outcomes	Not applicable

Dual use research of concern

Policy information about dual use research of concern

Hazards

Could the accidental, deliberate or reckless misuse of agents or technologies generated in the work, or the application of information presented in the manuscript, pose a threat to:

No	Yes	
☒	☐	Public health
☒	☐	National security
☒	☐	Crops and/or livestock
☒	☐	Ecosystems
☒	☐	Any other significant area

Experiments of concern

Does the work involve any of these experiments of concern:

No	Yes	
☒	☐	Demonstrate how to render a vaccine ineffective
☒	☐	Confer resistance to therapeutically useful antibiotics or antiviral agents
☒	☐	Enhance the virulence of a pathogen or render a nonpathogen virulent
☒	☐	Increase transmissibility of a pathogen
☒	☐	Alter the host range of a pathogen
☒	☐	Enable evasion of diagnostic/detection modalities
☒	☐	Enable the weaponization of a biological agent or toxin
☒	☐	Any other potentially harmful combination of experiments and agents

Plants

Seed stocks	Not applicable
Novel plant genotypes	Not applicable
Authentication	Not applicable

ChIP-seq

Data deposition

☐ Confirm that both raw and final processed data have been deposited in a public database such as GEO.

☐ Confirm that you have deposited or provided access to graph files (e.g. BED files) for the called peaks.

Data access links<br>*May remain private before publication.*	Not applicable
Files in database submission	Not applicable
Genome browser session<br>(e.g. UCSC)	Not applicable

Methodology

Replicates	Not applicable
Sequencing depth	Not applicable
Antibodies	Not applicable
Peak calling parameters	Not applicable
Data quality	Not applicable
Software	Not applicable

Flow Cytometry

Plots

Confirm that:

- [ ] The axis labels state the marker and fluorochrome used (e.g. CD4-FITC).
- [ ] The axis scales are clearly visible. Include numbers along axes only for bottom left plot of group (a 'group' is an analysis of identical markers).
- [ ] All plots are contour plots with outliers or pseudocolor plots.
- [ ] A numerical value for number of cells or percentage (with statistics) is provided.

Methodology

Sample preparation	Not applicable
Instrument	Not applicable
Software	Not applicable
Cell population abundance	Not applicable
Gating strategy	Not applicable

- [ ] Tick this box to confirm that a figure exemplifying the gating strategy is provided in the Supplementary Information.

Magnetic resonance imaging

Experimental design

Design type	Resting-state / anatomical (structural) MRI
Design specifications	Not applicable
Behavioral performance measures	Not applicable

Acquisition

Imaging type(s)	Structural MRI (T1-weighted)
Field strength	Mixed (multi-site cohorts; primarily 1.5T and 3T as provided by the originating studies)
Sequence & imaging parameters	Multi-site cohort acquisitions; specific pulse sequences and parameters (e.g., TR/TE/flip angle, voxel size) vary by site and scanner and were taken as provided by the originating cohorts.
Area of acquisition	Whole-brain structural MRI

Diffusion MRI [ ] Used [x] Not used

Preprocessing

Preprocessing software	- LPS orientation: AFNI (25.0.07), 3dresample (1.10), nifti_tool (2.13)<br>- N4 bias correction: ANTs (2.5.4-g8d891c2)<br>- Skull-stripping and MUSE extraction: CBICA-MUSE (3.1.0)
Normalization	Not applicable
Normalization template	Not applicable
Noise and artifact removal	Not applicable
Volume censoring	Not applicable

Statistical modeling & inference

Model type and settings	Predictive modeling was multivariate supervised classification (CN vs DA/ADRD) using XGBoost on MUSE-derived ROI features, with nested cross-validation and fairness-aware training/evaluation across race/ethnicity groups.
Effect(s) tested	Group-wise model performance differences (e.g., balanced accuracy, FNR, FPR) and cross-ethnic performance gaps (NHW vs NHA vs HISP; and generalization to "Other") across training scenarios/methods.

Specify type of analysis: ☐ Whole brain ☒ ROI-based ☐ Both

Anatomical location(s): Anatomical regions were defined using MUSE atlas–based automated parcellation applied to T1-weighted structural MRI. Regional measures (e.g., ROI volumes) were extracted for all MUSE-derived brain regions and used as model inputs.

Statistic type for inference

(See Eklund et al. 2016)

Two-sided statistical tests comparing performance and gap metrics across folds/runs (paired t-tests), as reported in the Supplementary Data.

Correction

No multiple-comparison correction was applied; p-values are reported with interpretation focused on consistent patterns across metrics and external cohorts.

Models & analysis

n/a	Involved in the study
☒	☐ Functional and/or effective connectivity
☒	☐ Graph analysis
☐	☒ Multivariate modeling or predictive analysis

Multivariate modeling and predictive analysis

Multivariate supervised classification was performed to predict dementia status (CN vs DA/ADRD) using MUSE-derived structural MRI ROI measures (e.g., regional volumes) as independent variables. Models were trained using XGBoost, with feature harmonization and group-balanced evaluation to control for confounding by sample imbalance and demographic differences across race/ethnicity groups. Model performance was assessed with balanced accuracy and error rates (FNR/FPR), and fairness was evaluated via cross-ethnic performance gaps and external validation on independent cohorts (ADNI and STAC) held out from training.
